# RitR is an archetype for a novel family of redox sensors in the streptococci that has evolved from two-component response regulators and is required for pneumococcal colonization

**DOI:** 10.1371/journal.ppat.1007052

**Published:** 2018-05-11

**Authors:** David G. Glanville, Lanlan Han, Andrew F. Maule, Alexandra Woodacre, Devsaagar Thanki, Iman Tajer Abdullah, Julie A. Morrissey, Thomas B. Clarke, Hasan Yesilkaya, Nicholas R. Silvaggi, Andrew T. Ulijasz

**Affiliations:** 1 Department of Microbiology and Immunology, Loyola University Chicago; Maywood, IL, United States of America; 2 MRC Centre for Molecular Bacteriology and Infection (CMBI), Imperial College London, London, United Kingdom; 3 Department of Chemistry and Biochemistry, University of Wisconsin-Milwaukee, Milwaukee, Wisconsin, United States of America; 4 Department of Horticulture, University of Wisconsin–Madison, Linden Drive, Madison, Wisconsin, United States of America; 5 Department of Genetics, University of Leicester, Leicester, United Kingdom; 6 Department of Infection and Immunity, University of Leicester, Leicester, United Kingdom; The University of Alabama at Birmingham, UNITED STATES

## Abstract

To survive diverse host environments, the human pathogen *Streptococcus pneumoniae* must prevent its self-produced, extremely high levels of peroxide from reacting with intracellular iron. However, the regulatory mechanism(s) by which the pneumococcus accomplishes this balance remains largely enigmatic, as this pathogen and other related streptococci lack all known redox-sensing transcription factors. Here we describe a two-component-derived response regulator, RitR, as the archetype for a novel family of redox sensors in a subset of streptococcal species. We show that RitR works to both repress iron transport and enable nasopharyngeal colonization through a mechanism that exploits a single cysteine (Cys128) redox switch located within its linker domain. Biochemical experiments and phylogenetics reveal that RitR has diverged from the canonical two-component virulence regulator CovR to instead dimerize and bind DNA only upon Cys128 oxidation in air-rich environments. Atomic structures show that Cys128 oxidation initiates a “helical unravelling” of the RitR linker region, suggesting a mechanism by which the DNA-binding domain is then released to interact with its cognate regulatory DNA. Expanded computational studies indicate this mechanism could be shared by many microbial species outside the streptococcus genus.

## Author summary

Most life requires elemental iron for survival. Bacterial pathogens are no exception, where within the host environment iron availability can “make or break” a successful infection. However, with healthy intracellular iron levels come great risks, as microbes must then strive to keep the needed iron from reacting with hydrogen peroxide and other oxidants, which can ultimately result in cellular damage and death. The bacterial pathogen *Streptococcus pneumoniae* is unusual in that it possesses both high intracellular iron and peroxide levels, a perceived recipe for suicide. To date, how *S*. *pneumoniae* and related pathogens contend with regulating this destructive cocktail has remained a mystery, as they lack all typical redox-sensing factors known to alert the cell of such danger. Here we describe the transcription factor RitR as an archetype redox-sensing regulatory protein in *S*. *pneumoniae* and related species. RitR works through alerting the cell of high peroxide levels in the airways through oxidation of its single cysteine residue, initiating DNA-binding to further repress the damaging iron from entering the cell. Importantly, this mechanism enables colonization of the nasopharynx, an event which is required for invasiveness. Analyses of bacterial genomes suggest that this mechanism could be conserved far outside the streptococcal species.

## Introduction

2.5 billion years ago when cyanobacteria introduced oxygenic photosynthesis the atmosphere became dominated by oxygen. In response, life on earth had to drastically change its chemistry to avoid the toxic effects of this new abundant gas and its byproducts from reacting with iron, an element which, up to this point, had extensively evolved to regulate many of life's metabolic processes [1]. Especially potent is the clash between hydrogen peroxide (H_2_O_2_) and ferrous iron (Fe^2+^), a reaction referred to as Fenton chemistry. This reaction produces the hydroxyl radical, a particularly serious reactive oxygen species (ROS) that can damage most biological material and ultimately result in cell death [2]. In present times little has changed; with a few exceptions [3], most bacteria are still reliant on iron to perform crucial metabolic functions. In addition to atmospheric oxygen and associated ROS, pathogens have to contend with phagocytes that use hydrogen peroxide and other ROS as a weapon to kill invading bacteria. Therefore, to help regulate the often coexisting iron content and encountered peroxide levels, bacteria have evolved an array of regulatory proteins to sense changes and elicit a timely response to ROS and ROS-related damage (for review see Ezraty *et al*. [4]). These sensors often involve oxidation of the strong nucleophile cysteine (Cys), an amino acid that can undergo a variety of chemical reactions to enable accurate sensing of the immediate redox environment [5, 6].

In both Gram-positive and Gram-negative bacteria, several families of peroxide-sensing transcription factors have been described in detail, such as the OhrR, OxyR, and PerR families [6–12]. Interestingly, while most bacteria have identifiable homologs of these and/or other peroxide sensory transcription factors and response pathways, none have been identified in the human pathogen *Streptococcus pneumoniae* (the pneumococcus) or related streptococcal species [13]. Even more perplexing is that in addition to having comparatively high intracellular iron levels to most iron-utilizing microbes [14], in air-rich environments the pneumococcus produces millimolar quantities of hydrogen peroxide as a metabolic byproduct of pyruvate oxidase (or SpxB), an activity which has been shown to regulate pneumococcal capsule formation [15–17], and aid this pathogen in both colonization of the upper respiratory tract and infection of the heart [18, 19]. Interestingly, pneumococcal SpxB-produced peroxide carries out these functions in the absence of any canonical catalase [13, 14]. Recent work has shed some light on this subject showing that two surface exposed thioredoxin-family lipoproteins, Etrx1 and Etrx2 (formally TplA), are key to ROS remediation [20, 21]. However, although several reports have linked various regulatory factors such as Rgg [22], NmlR [23, 24], RitR [25–27], PsaR [28] and CiaRH [29] with regulation of the pneumococcal oxidative stress tolerance, a *bona fide* Cys-activated redox transcription factor able to respond to these high peroxide and iron levels has remained largely enigmatic (for review see Yesilkaya *et al*. [13]).

Here we describe the two-component-like orphan response regulator RitR (Repressor of iron transport Regulator) as the archetype of a novel family of peroxide-sensing transcription factors in the pneumococcus and related species. Previous work demonstrated that RitR acts to repress iron uptake via binding the pneumococcal iron uptake (Piu) transporter promoter [25]. RitR contains an Aspartate-less Receiver (ALR) REC-like domain [30], which retains the common (α/β) fold of canonical REC versions, yet lacks “invariant” residues in its catalytic pocket required for typical phosphotransfer, including the phospho-accepting Asp residue crucial for the histidine kinase-response regulator phosphorelay. [31–33]. Based on these observations, we reasoned that RitR is regulated by an atypical mechanism. Here we show that RitR regulates pneumococcal iron homeostasis in response to cellular peroxide concentrations through its single Cys (Cys128) located within the linker domain of the protein. We go on to demonstrate that Cys128 helps to control pneumococcal nasopharyngeal colonization, which is a prerequisite to invasive disease and the communal spread of this pathogen [34, 35]. Atomic structures of RitR in its oxidized and reduced states demonstrate a dramatic mechanism of activation that hinges on the helical unraveling and subsequent formation of an inter-protomer disulfide bridge to enable DNA binding and transcriptional regulation of Piu. Data generated from custom algorithms to identify the frequency and distribution of Cys residues within REC-containing bacterial transcription factor linker sequences suggest that this mechanism of activation could be widespread in nature, reaching far outside the streptococci.

## Results

### Cys128 is required for repression of Piu transcription and translation

Previous work has demonstrated that RitR responds to H_2_O_2_ stress and regulates cellular metal ion homeostasis [13, 25–27]. From these data we suspected that RitR might be responding directly to oxygen, or oxygen-generated ROS, through its single Cys residue (Cys128) located within the linker domain of the protein that connects the Aspartate-Less Receiver (ALR) domain [30] to the DNA-binding domain (DBD). To investigate the contribution of Cys128 to RitR function, we constructed a series of mutants in both R800 (unencapsulated) and D39 (encapsulated) strains: ΔritR full deletion, C128A (oxidation dead), C128S (reduced mimetic), C128D (oxidized mimetic) and *ritR* wild-type chromosomal reconstructions (see Methods section for details). These mimetics have been used to determine the mechanism some of the most classic examples of redox-sensing transcription factors, *e*.*g*. OxyR and SsrB [36, 37].

**Fig 1:**
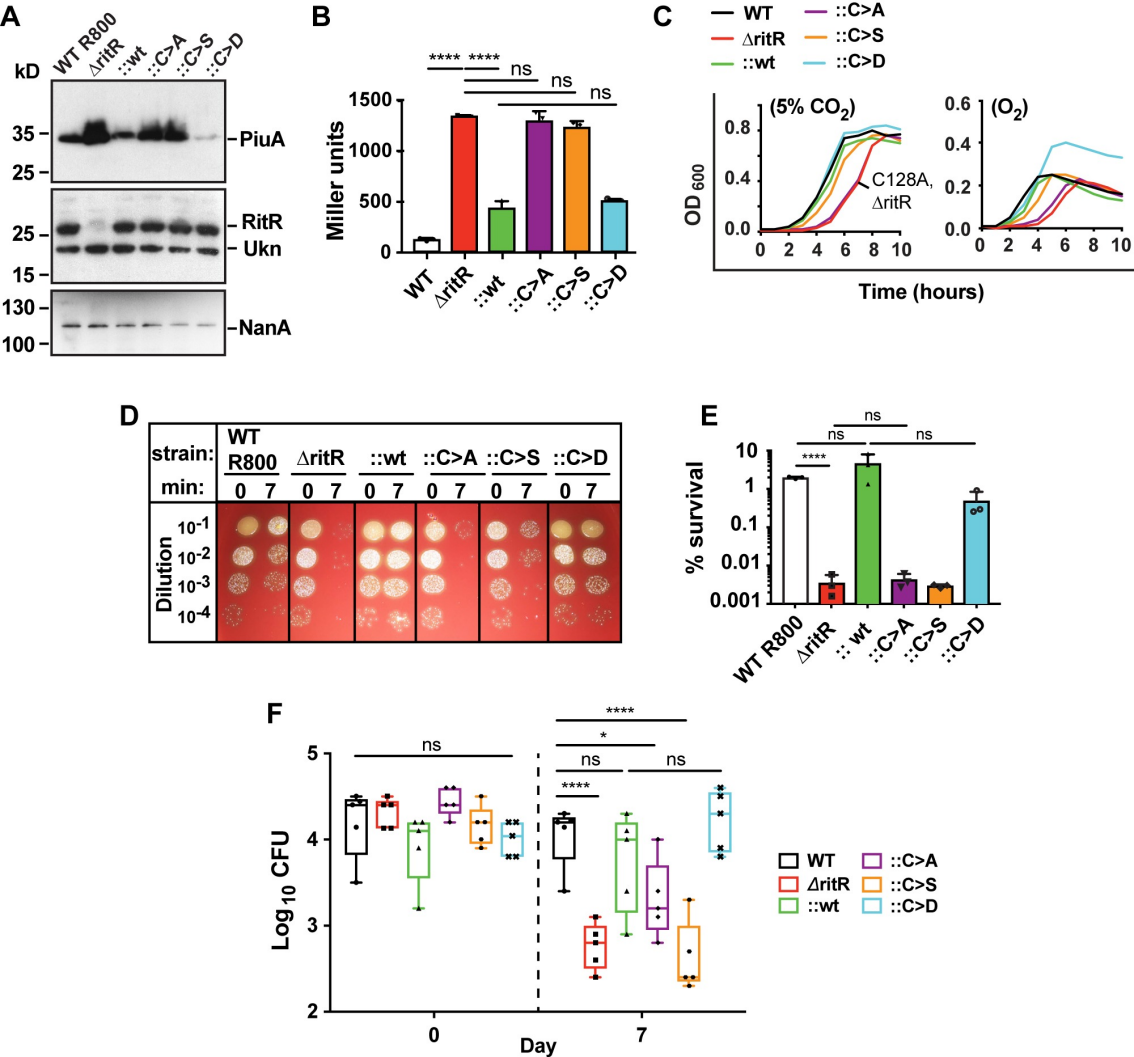
Cys128 is required for growth and regulation of the Piu operon and colonization. (**A**) Western blot analysis representative of three independent experiments of PiuA iron transporter lipoprotein levels in the R800 *ritR* genetic background variants grown in 5% CO_2_. RitR and NanA are used as controls. Ukn, an unknown cross-reacting protein. (**B**) Piu promoter activity in the genetic background of R800 *ritR* variants in 5% CO_2_ as measured by beta-galactosidase activity and expressed in Miller units. (**C**) Growth comparison of wild-type D39 cells plus *ritR* variants under static 5% CO_2_ and aeration (O_2_) conditions. (**D**) Streptonigrin killing assay representative of three biological repeats. (**E**) Bar graph of three independent experiments as shown in *D*. (**F**) Ability of D39 variants to colonize the murine nasopharynx. Mice were inoculated and the colonization let run for 7 days before cells were collected and plated to determine CFUs. *Statistics*. Colonization data were analyzed by analysis of variance followed by Tukey’s multiple comparisons test. Statistical significance was considered to be a p-value of < 0.05. Each point indicates the CFUs retained in a single animal. Graphs in *B* and *E* represent the mean of three independent experiments. Error bars represent +/- standard deviation. One asterisk indicates a p-value of ≤0.05, two of ≤0.01, three of ≤0.001, and four of ≤ 0.0001 as determined by one-way ANOVA followed by Tukey’s multiple comparison test in *B*, and a two-tailed students t-test in *E*. ns, not significant.

The effects of Cys128 on Piu expression were examined by performing qRT-PCR and western blot analysis to assess *piu* operon RNA and PiuA protein levels, respectively. PiuA is an iron/heme solute binding protein and one of the four genes encoded within the Piu iron/heme uptake operon [25, 38]. Results in **Figs 1A** and **S1A** and **S1B** show that both *piu* RNA and PiuA protein levels increased substantially in the ΔritR, C128A and C128S strains. Conversely, the C128D oxidative mimetic strain demonstrated a marked repression of PiuA protein levels and *piu* operon RNA levels, even surpassing wild-type repression in the R800 genetic background. RNA levels of the 6-*phosphogluconate-ritR* operon (*gnd-ritR*) transcript and RitR protein levels remained constant, as did levels of control proteins NanA (the pneumococcal neuraminidase A protein) and the solute binding protein PiaA (**Figs 1A** and **S1A** and **S1B**). PiaA is a component of another iron transporter system (Pia) not known to be controlled by RitR [38]. Overall, these results support the hypothesis that Cys128 facilitates repression of the Piu operon in the oxidized form.

### Cys128 affects Piu promoter activation

To further test if Cys128 was required to modulate Piu iron transport synthesis we constructed Piu promoter β-galactosidase reporters within the context of the aforementioned D39 and R800 genetic backgrounds. Results shown in **Figs 1B** and **S1C** revealed that in the ΔritR, C128A and C128S strains Piu promoter activity was highly elevated compared to wild-type levels, indicating that these RitR variants are attenuated in their ability to repress Piu promoter activation. In contrast, the C128D variant exhibited repression of activity comparable to the wild-type complemented indicator strains, suggesting that Cys128 oxidation initiates repression of Piu iron uptake genes.

### RitR Cys128 affects pneumococcal growth *in vitro*

To observe if Cys128 influenced growth, we cultured and measured the optical density of our wild-type D39 pneumococcal cells and associated mutants in typical conditions (5% CO_2_, static) and also under aeration (O_2_, shaking). Results shown in **Fig 1C** indicate that the ΔritR and C128A mutants exhibited a clear lag in growth as compared to WT cells, whereas the C128S variant produced an intermediate growth defect. As expected from previous studies, when aerated pneumococcal cells generally grew to lower optical densities, with the C128S, C128A and ΔritR mutants paralleling the observed lagging phenotype of the CO_2_ cultured cells. However, the C128D mutant was much less affected by aeration, where no comparative lag was observed and the cells surprisingly attained an optical density of over 0.4, approximately twice that of wild-type (**Fig 1C**). These observations were highly reproducible. Our results suggest that Cys128 and its state of oxidation influences pneumococcal growth in an oxygen-dependent manner.

### Cys128 is responsible for regulation of intracellular ferrous iron content

RitR regulates iron and H_2_O_2_ toxicity by binding the Piu promoter to repress iron uptake [13, 25, 27]. To determine if Cys128 could influence pneumococcal cellular iron content, we challenged our RitR variants with streptonigrin, an antibiotic whose action is ferrous (Fe^2+^) iron-dependent and has been used to compare pneumococcal ferrous iron content in differing strains and conditions [25, 38]. To enable sufficient iron uptake and minimize any differences that might be due to altered regulation of iron uptake systems independent of RitR regulation, pneumococcal cells were first starved in iron depleted medium before the introduction of freshly prepared ferrous iron and streptonigrin treatment. **Fig 1D** and **1E** show the effects on R800 variants after a 7-minute streptonigrin incubation. The ΔritR, C128A and C128S strains exhibited greater than two orders of magnitude higher susceptibility compared to the C128D and wild-type strains. Parallel experiments in D39 cells showed similar trends (**S1D Fig**). These results strongly suggest that the oxidized form of RitR Cys128 represses iron uptake and promotes the quantitative reduction of intracellular free ferrous iron concentrations.

### RitR and Cys128 contribute to pneumococcal colonization

Previous publications have presented conflicting data regarding the role of RitR during infection of the airways [25, 27]. However, the effect of RitR on colonization has not been determined. Interestingly, a *tn*-seq mass sequencing study determined that a *ritR* deletion in the TIGR4 (serotype 4) strain was lethal [39]. This is obviously not the case with D39 (serotype 2) used in the present studies. The colonization model mimics the pneumococcal carrier state, which is its most oxygenated environment (the nasopharynx). Within the upper respiratory tract iron toxicity would be expected to be most prevalent due to increased H_2_O_2_ production from SpxB activity [13], which has actually been shown to aid in pneumococcal colonization of both the nasopharynx [19], and more recently cardiac tissue [18]. Given these obvious ties to RitR function we therefore deemed it important to study the effect of RitR and Cys128 on pneumococcal colonization and performed a well-established murine colonization model [40, 41] with our D39 strains.

Results showed that when compared to wild-type D39 and wild-type complement strains, after 7 days the resultant colony forming units (CFUs) collected from the nasopharynx revealed a marked and statistically-significant decrease with the ΔritR strain (1.4 log decrease), C128A strain (1.0 log decrease) and most of all the C128S reduced mimetic strain (1.8 log decrease) (**Fig 1F**). Conversely, the C128D oxidized mimetic mutant slightly increased in CFU numbers compared to that of wild-type (by a log of 0.1) (**Fig 1F**). These data suggest that RitR Cys128 oxidation is required for optimal colonization of the nasopharynx in D39 serotype 2, and therefore by inference, downstream infections and community spread of pneumococcal disease [34, 35].

### Hydrogen peroxide is the dominant activator of RitR Cys128-mediated dimerization

**Fig 2:**
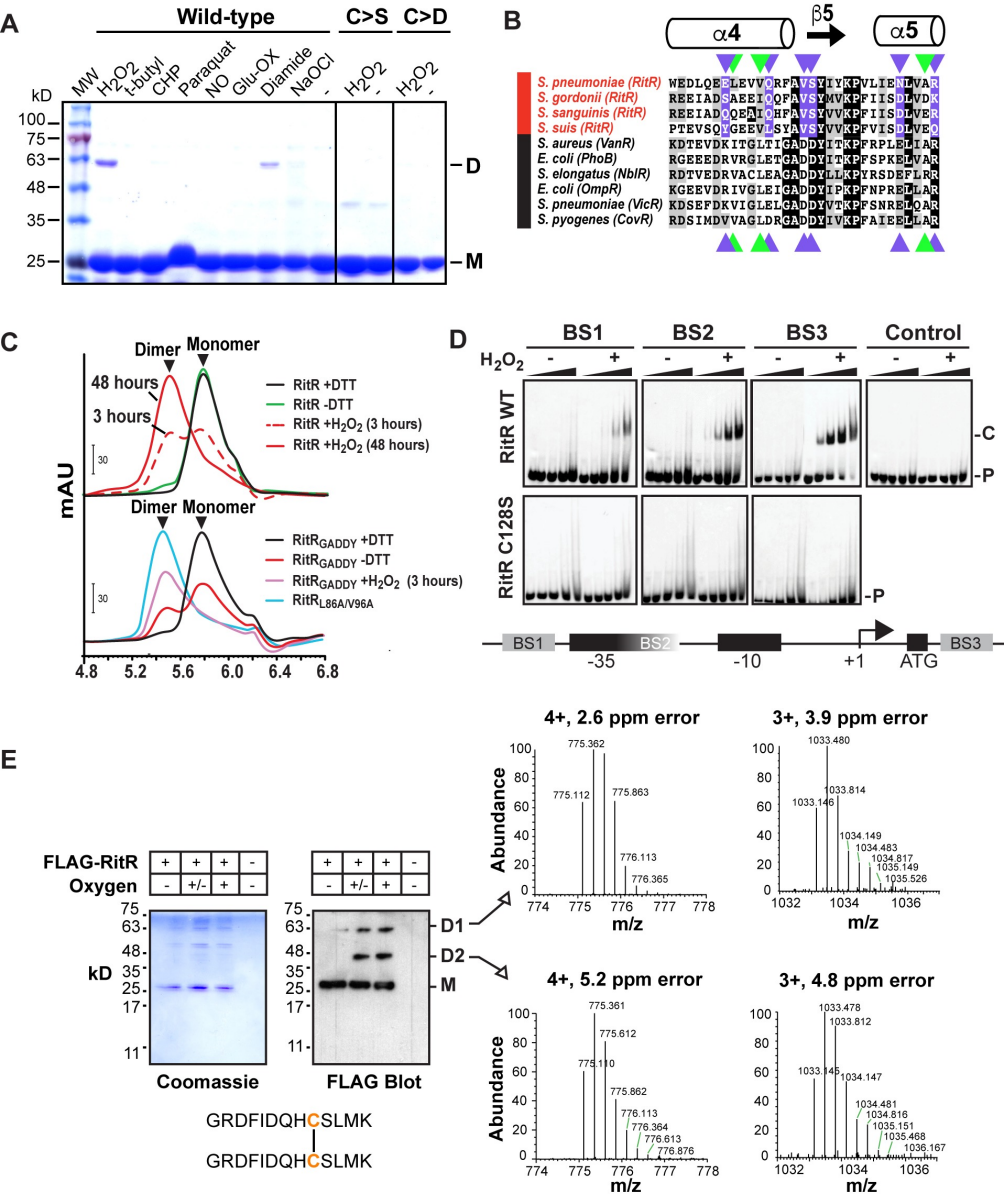
RitR has evolved to dimerize through Cys128-mediated oxidation by H_2_O_2_. (**A**) Non-denaturing SDS-PAGE gels of RitR WT, C>S and C>D mutants plus various oxidants in the presence of 3 mM DTT. D, dimeric RitR; M, monomeric RitR. MW, Molecular Weight ladder; H_2_O_2_, hydrogen peroxide; t-butyl, tert-butyl hydroperoxide; CHP, cumene hydroperoxide; NO, nitrous oxide; Ox-Glu, oxidized glutathione; NaOCl, sodium hypochlorite. (**B**) Alignment of REC/ALR α4-β5-α5 dimerization domains [30] from RitR homologs (red) and canonical REC domain sequences (black). The black boxes are identical residues and the grey boxes are similar residues. Residues colored purple and green represent key charged and hydrophobic residues, respectively, involved in typical REC dimerization. Note that several of these key residues are changed in RitR homologs. (**C**) SEC of wild-type RitR with (+) or without (-) addition of DTT or H_2_O_2_ (top graph), or RitR changed back to the canonical GADDY sequence (RitR_GADDY_; bottom graph). For comparison, the RitR_L86A/V96A_ mutant is shown, which naturally dimerizes without addition of oxidant [30]. mAU; milli Absorbance Units. (**D**) EMSAs of RitR wild-type (WT) and the C128S mutant in the presence (+) and absence (-) of H_2_O_2_. RitR proteins were added at 0, 0.22, 0.66, 2.2 and 6.6 μM concentrations (left to right) in the presence of hexofluorescein (HEX)-labeled BS1-3 double-stranded DNA oligomers. A HEX labeled control oligo was also used. P, Hex DNA probe; C, RitR-DNA shifted complex. Below is a schematic diagram of the Piu promoter and regulatory region showing the location of RitR binding sites 1–3 (BS1-3) as previously described [25]. (**E**) Mass spectrometry (MS) analysis of the Cys128 disulfide bridge formation *in vivo*. Upper (D1), middle (D2) and lower bands (M) of RitR as identified from the anti-FLAG western blot and accompanying Coomassie stain were excised from the gel and determined to contain RitR using MS. (+) oxygen = cells were aerated, (+/-) oxygen = cells were grown statically in 5% CO_2_, and (-) oxygen = cells were grown anaerobically before addition of IAA and RitR immunoprecipitation. The MS identified Cys128 linked peptide is shown with Cys128 colored in orange. Data shown in A-E are representative of at least two independent experiments.

Most redox-sensing transcription factors are responsive to specific cellular oxidants [6]. To identify the precise oxidant(s) to which RitR responds through Cys128, we first dialized RitR wild-type, C128S or C128D proteins into 3 mM dithiothreitol (DTT) in order to mimic the normal reduced environment of the cell, and then proceeded to add an excess of select oxidants. The RitR samples were then resolved by non-reducing SDS-PAGE. Results shown in **Fig 2A** demonstrate that only H_2_O_2_, and to a much lesser extent diamide, produce a marked dimeric form of RitR. These results suggest that at least *in vitro*, H_2_O_2_ is a specific oxidant that acts on RitR Cys128 to initiate and enable dimerization.

### RitR has diverged from a typical two-component response regulator mechanism to instead activate through cysteine-mediated dimerization

To further investigate the oligomeric properties of RitR we exploited Size Exclusion Chromatography (SEC). Upon close inspection of the RitR sequence, we noticed that RitR has lost several highly conserved acidic residues that usually comprise the REC “GADDY motif” (*e*.*g*. Val98 and Ser99; see residues in **Fig 2B** colored purple). This conserved sequence normally contributes to hydrogen bonding and dimer formation within canonical REC domains [30, 42]. From this observation we hypothesized that unlike typical two-component response regulators that rely on the GADDY sequence to dimerize, the absence of a GADDY motif in RitR might explain why it is solely reliant on Cys128 to form its dimer complex. To test this hypothesis, RitR was reverse engineered by replacing the “FAVSY” residues (where based on alignments the GADDY sequence should be) back to the canonical GADDY motif shared by most REC domains (RitR_GADDY_; **Fig 2B;** residues 98–101 **in S4 Fig**). RitR wild-type and the RitR_GADDY_ variant proteins were then assessed for their oligomeric states in solution by SEC in the presence or absence of DTT (reductant) or H_2_O_2_ (oxidant).

Results showed that wild-type RitR is essentially monomeric both in the presence and absence of the reductant DTT, and increasingly becomes dimeric over time in the presence of H_2_O_2_ (**Fig 2C**, top panel). We similarly observed a monomeric peak with the RitR_GADDY_ construct when reduced (+DTT; **Fig 2C**, bottom panel). However, differing from the equivalent wild-type RitR SEC profile, when DTT was removed from the RitR_GADDY_ construct a partial reversion to the dimeric form was observed, which could then be driven to ~100% dimer after addition of H_2_O_2_ oxidant (**Fig 2C**, bottom panel). For reference we included a SEC profile of the RitR_L86A/L96A_ mutant which resides in the dimeric form only [30]. These results lend strong support to the hypothesis that RitR has evolved to form a dimer only through the oxidation of Cys128 by ridding itself of crucial residues within the α4-β5-α5 interface, normally responsible for facilitating the phospho-Asp activated dimeric state (**Fig 2B**) [30, 42].

### H_2_O_2_ induces Cys128-mediated RitR binding to its target DNA

As RitR is known to directly bind and repress Piu operon transcription [25], we wanted to investigate if Cys128 was facilitating its interaction with the Piu operon regulatory region. To test this we performed Electrophoretic Mobility Shift Assays (EMSAs) using RitR wild-type and C128S mutant protein with target *piu* RitR regulatory binding sites (defined as Binding Sites 1–3, or BS1-3) [25]. Again, RitR protein was first dialized into the DTT reductant before incubation with excess H_2_O_2_, addition to BS1-3 double stranded DNA and fractionation by non-reducing native electrophoresis.

Results shown in **Fig 2D** clearly demonstrate that RitR binds BS1-3 target DNA only in the presence of H_2_O_2_ oxidant. In contrast, no appreciable DNA binding was observed in the presence of a scrambled control oligo, or the RitR_C128S_ mutant reactions (**Fig 2D**). These data suggest that RitR binding and repression of the Piu promoter is activated via H_2_O_2_ oxidation through Cys128.

### Aeration induces RitR dimerization *in vivo*

Thus far, our *in vitro* studies have shown RitR dimer-mediated binding to DNA in the presence of the specific oxidant H_2_O_2_, a natural metabolic byproduct of pneumococcal pyruvate oxidase activity (SpxB) in high oxygen environments [13, 43]. From these results we reasoned that if H_2_O_2_ was only known to be significantly produced in the presence of oxygen and/or aeration [13, 14], then by inference, only in such an oxygen-rich environment would RitR dimerize *in vivo*. To explore this possibility, we first measured the relative levels of H_2_O_2_ produced in both D39 and R800 cells under (1) static in 5% CO_2_, (2) anaerobic and (3) aerobic growth conditions. Results paralleled previous studies [14], where only 2.4–3.5 μM H_2_O_2_ concentrations were detected under anaerobic conditions, 0.6–0.7 mM in 5% CO_2_ and as much as 1–3 mM when aerated (**S1E Fig**). To then test whether aeration and the measured intracellular H_2_O_2_ levels affected RitR dimerization *in vivo*, we expressed N-terminal FLAG-tagged RitR in R800 ΔritR cells. Importantly, this tagged RitR strain exhibited the same Piu repressive phenotype as strains expressing the non-tagged RitR versions (**S2A Fig**). Next, FLAG-RitR was immunoprecipitated from cells grown under the three oxidation conditions (-O_2_, CO_2_ and +O_2_), fractionated via non-reducing SDS-PAGE, and finally mass spectrometry was performed on excised bands detected with anti-FLAG antibodies.

Results shown in **Fig 2E** indicate that only in the presence of oxygen (*i*.*e*. in either 5% CO_2_ or when aerated) did RitR form higher order dimeric complexes. In addition to the FLAG-RitR monomer (M) at the predicted mass of 27,874 kD, two distinctly higher molecular weight bands were present in the CO_2_ and aerated samples: one at approximately 57 kD (D1), which is close to the predicted molecular weight of the RitR dimer (54,570 Da precisely), and one at approximately 45 kD (D2), which could represent either a degraded form of the RitR dimer, or could be a heterologous complex between RitR and another unknown protein. As expected, in the C128A samples only the RitR monomer was observed, indicating that the higher molecular weight complexes were mediated specifically through Cys128 oxidation (**S2B Fig**).

Next, we wanted to verify that RitR was indeed contained within the three bands detected in the anti-FLAG western blots (*i*.*e*. **Figs 2E** and **S2B**). To accomplish this, all three bands (M, D1, and D2) were excised and subjected to mass spectrometry analysis. **S2C**–**S2E Fig** show the presence of RitR and good coverage for all three RitR bands, effectively eliminating the possibility that D2 is a degraded D1 RitR product. When standard Mascot searches were performed to detect the presence of RitR peptides, we did not observe any coverage of the peptide encompassing Cys128 in the higher molecular weight (D1-2) samples (residues Gly120 to Arg135). In fact, this peptide was only found in the RitR monomeric run (**S2C Fig**). These findings suggest that a peptide containing the Cys128 disulfide bridge was retained in the mass spectrometry treatments of the D1-2 samples. In further support of these findings, a more detailed search indeed revealed the presence of a set of mass peaks which were consistent with the GRDFIDQHCSLMK disulfide bridged peptide (residues 120–132), which had a calculated molecular mass of 2.6–5.2 ppm that falls within the error of the instrument (**Fig 2E**). We found the peptide corresponding to charged species of +3 and +4 (in both D1 and D2 samples), which would be expected due to its high amount of charged residues (Arg, Lys, His). Indeed, in both the higher (D1) and lower (D2) molecular weight bands we found no evidence of the monomeric form of the peptide, indicating that our Cys128 disulfide bridge was maintained to near 100% during treatment in these samples before the mass spectrometry was performed. In contrast, the monomeric GRDFIDQHCSLMK peptide was readily identified in the monomer sample (**S2C**–**S2E Fig**). Taken together, these results show that RitR dimerizes in response to oxygen *in vivo* through Cys128, and is essentially fully monomeric under anaerobic conditions.

### Atomic structure of the RitR_C128S_ reduced mimetic and RitR_C128D_ oxidized mimetic

The X-ray crystal structure of the full-length reduced state mimic RitR_C128S_ was determined to 1.7 Å resolution with selenomethionine (Se-Met) Single Wavelength Anomalous Diffraction (SAD) method (**Table 1**). As previously discussed, RitR is found predominantly in the monomeric state in the absence of an oxidizing agent. However, under oxidizing conditions, in solution a small proportion of RitR can exist in the dimeric form (**Fig 2C**, upper panel), and can even persist in the presence of reducing agents. This slight but significant heterogeneity is likely the factor that prevented crystallization of the full-length, reduced form of RitR during the course of our studies. Thus, to eliminate any heterogeneity caused by this phenomenon, we mutated the redox-active cysteine residue (Cys128) to serine, which would be unable to oxidize and activate the protein. The resulting RitR_C128S_ exhibited no evidence of dimerization in solution using both SEC and 2-dimensional nuclear magnetic resonance (NMR) techniques (**S9 Fig**). The RitR_C128S_ (Se-Met) mutant crystallized in space group C2 with two molecules in the asymmetric unit. These molecules are nearly identical with root mean square deviations of 0.69 Å for all Cα atoms, are independent, and do not form a dimer with each other, nor with any symmetry-related molecule.

**Table 1:** Crystallographic data collection and refinement statistics.

	RitR_C128S_ (5U8K)	RitR_C128D_ (5VFA)	RitR_ox_ (5U8M)
Space group	C2	C2	P2_1_2_1_2_1_
Cell dimensions			
a, b, c (Å)	141.1, 60.1, 53.3	142.8, 59.7, 52.5	74.3, 74.8, 102.8
α, β, γ (°)	90, 96, 90	90, 96.3, 90	90, 90, 90
Resolution (Å) (last shell)^a^	40.33–1.69 (1.75–1.69)	50–1.45 (1.48–1.45)	50.00–2.10 (2.14–2.10)
Wavelength (Å)	0.97872	0.97857	0.97872
No. of reflections			
Observed	276131 (24543)	396912 (14482)	206898 (8842)
Unique	49527 (4856)	76689 (3384)	33913 (1667)
Completeness (%)^a^	99.6 (97.9)	99.0 (89.1)	99.9 (99.6)
R_merge_^a^^,^^b^	0.090 (0.340)	0.046 (0.498)	0.071 (0.586)
CC1/2 in last shell	0.931	0.901	0.879
Multiplicity	5.6 (5.1)	5.2 (4.3)	6.1 (5.3)
<I/σ(I)>^a^	16.6 (8.1)	28.6 (2.9)	25.2 (2.5)
**Model Refinement Statistics**			
Reflections in work set	91682	76664	64031
Reflections in test set	3718	2000	3805
R_cryst_ (R_free_)	0.144 (0.169)	0.164 (0.185)	0.188 (0.231)
No. of residues	459	443	458
No. of solvent atoms	733	610	185
Number of TLS groups	18	16	17
Average *B*-factor (Å^2^) ^c^			
Protein atoms	12.9	25.9	46.3
Solvent	27.4	36.4	40.7
RMS deviations			
Bond lengths (Å)	0.010	0.008	0.011
Bond angles (°)	1.02	0.98	1.05
Coordinate error (Å)	0.11	0.14	0.26
Ramachandran statistics (favored/allowed/outliers)	98.5/1.5/0.0	97.0/3.0/0.0	98.0/2.0/0.0

^a^ Values in parentheses apply to the high-resolution shell indicated in the resolution row.

^b^ *R* = Σ(||F_obs_|-scale*|F_calc_||) / Σ |F_obs_|.

^c^ Isotropic equivalent B factors, including contribution from TLS refinement.

**Fig 3:**
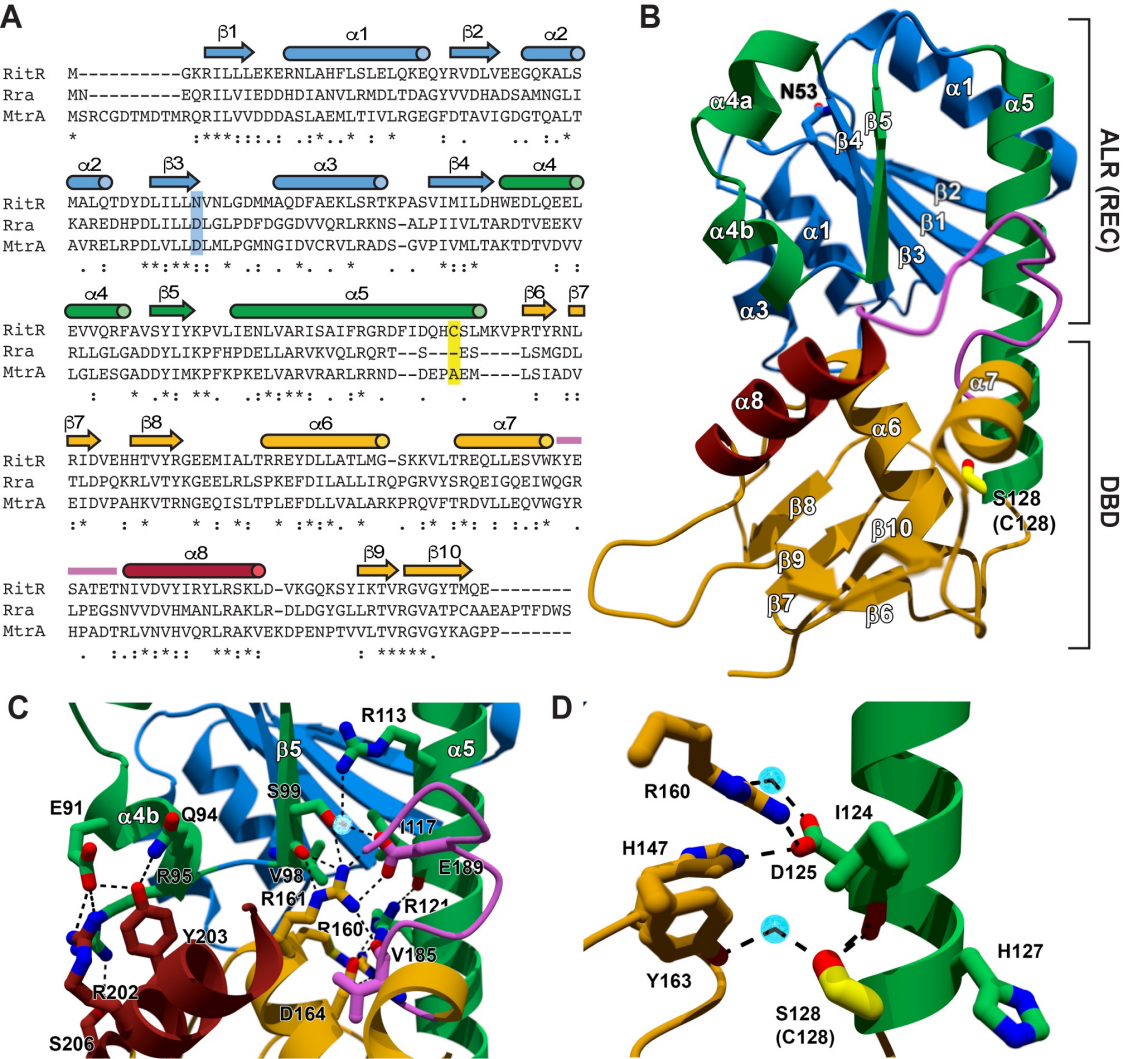
Structure of the ‘reduced’ RitR C128S. (**A**) RitR Clustal Omega annotated alignment of RitR and two other full-length response regulators with available structures (MtrA from *Mycobacterium tuberculosis*, PDB ID 2GWR, and Rra from *Deinococcus radiodurans*, PDB ID 3Q9S). Identity is denoted by an asterisk and similarity by dots/colons. Secondary structure depicted above the sequences is color coordinated with the 3D models presented in *B-D*. The reactive Cys128 position is shaded in yellow, and the position which normally contains the phosphorylated Asp residue shaded in blue (note in RitR it is an Asn instead). (**B**) Ribbon diagram of the full-length, monomeric ('reduced' / inactive) RitR_C128S_. Ser128 (Cys128 coordinate) is labeled, colored yellow and appears in ball-and-stick format. The α4-β5-α5 face of the REC domain used by canonical response regulators for dimerization is shown in green. The remainder of the REC domain is blue. The DNA-binding domain is gold, save for the recognition helix (red) and the trans-activation loop (magenta) that interacts with RNA polymerase to direct transcription [48, 49]. ALR, Aspartate-less receiver domain [30]; DBD, DNA-binding domain. (**C**) Close-up of the RitR DBD-REC interface shown with the same coloring. The residues comprising the interface are shown as ball-and-stick. (**D**) Close-up of the Ser128 (Cys128 coordinate) interactions with neighboring residues and water molecules. Dotted lines denote predicted electrostatic interactions. Oxygen atoms are shown in red, nitrogens in blue and water molecules as light blue circles. Images were created using MOLSCRIPT and POVRay [95].

The structure of the N-terminal Aspartate-Less Receiver (ALR) domain of RitR_C128S_ (hereon referred as its REC domain; [30]) is almost identical to that of our previously reported RitR REC domain-only structure, consisting of the first 124 residues of the protein (PDB accession code 4LZL [30]). In support, the root mean square deviation (RMSD) obtained from least-squares fitting of the RitR REC domain structure to that of full-length RitR_C128S_ REC is only 0.28 Å for all Cα atoms up to residue 106 (the beginning of helix α5). If helix α5 is included in the fitting, the RMSD increases to 0.78 Å, owing to a slight reorientation of this helix relative to the rest of the tertiary structure (**S3A Fig**). The RitR DBD structure is very similar to those of other helix-turn-helix response regulators, including DrrB from *Thermotoga maritima* (PDB accession code 1P2F [44]) and MtrA from *Mycobacterium tuberculosis* (PDB accession code 2GWR [45]) (**Figs 3A** and **S3B**). When superposed onto the RitR atomic structure by least square fitting, the DNA-binding domain of DrrB produced an RMSD of 5.8 Å (23.5% sequence identity), whereas the RMSD of MtrA was 1.2 Å (40.4% sequence identity). The MtrA closer fit was attributed to more similar confirmation of MtrA to RitR, rather than overall topology of the structure.

A notable feature of the RitR_C128S_ structure is that helix 8 of the RitR DBD, which is known to interact with the major groove of DNA in response regulators, is sequestered within its REC-DBD inter-domain region, thereby rendering it inaccessible to DNA binding ([46, 47]; colored brick red in **Fig 3B** and **3C**). This ~850 Å^2^ REC-DBD interface of RitR_C128S_ is stabilized by several salt bridges and hydrogen bonds that include Glu91-Arg202, Glu91/Gln94-Tyr203, and Arg95-Ser206. Indeed, based on previously published structures, in the activated form RitR helix 8 residues Arg202, Tyr203 and possibly Ser206 would be predicted to bind DNA and their α4b-α5 Glu91/Gln94/Arg95 hydrogen binding partners would instead facilitate REC domain dimeric interactions (**Fig 3C**) [30]. To add further support to this hypothesis, we first aligned the DNA binding domains of RitR and the canonical response regulator PhoB from *E*. *coli* (PBD code 1GXP [47]). **S5A Fig** shows a high similarity between residues by which PhoB interacts with its cognate DNA and that of RitR, including (RitR coordinates) Trp186, Arg161, Arg202, Tyr200, Tyr203, Arg222 and Thr219 (**S5A–S5C Fig**). Of these residues Tyr200, Arg202 and Tyr203 of helix 8, and other DNA-binding domain residues (Trp186 and Arg161) comprise a significant portion of the buried DBD elements sandwiched by REC domain electrostatic interactions (**S5D–S5E Fig**). A comparison of electrostatics between PhoB and RitR DNA-binding domains shows parallel positioning of some positively charged residues between structures predicted to interact with DNA (**S5F Fig**). However, more positive charges are visibly present in PhoB, as RitR contains several aromatic amino acids in these same positions, which are also (notably) able to hydrogen bond with DNA (**S5A Fig**). Finally, we observe that the trans-activation loop (colored magenta in **Fig 3**), responsible for the binding and direct recruitment of RNA polymerase [48, 49], would be inaccessible to RNAP in the RitR_C128S_ structure. Combined with the fact that RitR requires oxidation to bind and repress Piu transcription, from these studies we can be fairly confident that the RitR_C128S_ structure is reflective of the inactive, reduced state.

The most unique and striking feature of the RitR_C128S_ structure is the α5 helix linker, which connects the REC and DBD sections of the protein. The RitR linker domain runs almost the entire length of the protein, where it maintains an easily-solvable helical structure (**Figs 3B** and **S3A–S3B**). This feature is emphasized in that, as far as we are aware, no other full-length two-component response regulator structures have exhibited such a continuously well-structured linker extension. For example, if we compare the RitR structure to a more typical homolog (MtrA), the C-terminal portion of the α5 in MtrA exhibits the usually seen, largely unstructured linker extension (**S3B Fig**).

At the end of the RitR linker helix (α5) sits the "HCS" motif (**Fig 3A–3B**) that is conserved across RitR homologs (for a complete alignment see **S4 Fig**). Thiolate anions (RS-) found in cysteines are excellent nucleophiles, and therefore possess enhanced reactivity to H_2_O_2_ [5]. However, some cysteines are more susceptible to oxidation than others depending on their surrounding environment and resultant pKa in the folded 3-dimensional protein structure. Such modulation of a particular Cys pKa from its “free” form possessing a basic pKa of 8.5 determines its oxidation reactivity, where more acidic Cys residues will succumb to oxidation more easily in the presence of extreme levels of H_2_O_2_, as seen, for example, in the case of high pneumococcal SpxB activity. With this in mind, we used a standard DTNB (5,5-dithio-bis-(2-nitrobenzoic acid), or Ellman’s reagent) assay to obtain the pKa of Cys128, which was determined to be more acidic than free cysteine (Cys128 pKa = 6.85) (**S3E Fig**). The 6.85 pKa falls within the upper end of the expected range for a redox-sensing cysteine residue, which can range from a pH of 3–6 and would put the majority of the Cys side chain in its thiolate form at a physiological pH of 7.4 [50]. However, since the pneumococcus is naturally tolerant to its high intracellular peroxide levels [14, 17], it is reasonable to speculate that *S*. *pneumoniae* redox-sensing cysteines such as Cys128 might have a higher threshold of activation (enabled by its more basic pKa in comparison to cysteine-activated sensors form other less peroxide-tolerant species).

The environment of the cysteine thiol group, as inferred from the position of S128 Oγ in our structure, is quite polar (**Fig 3D**), though the structure provides no clear evidence of how the pK_a_ of Cys128 is perturbed to enable its more acidic readout of 6.85. If anything, the location of Cys128 at the C-terminus of α5, and the potential interactions of the thiol group with the main chain carbonyls of Ile124 and Asp125 (**Fig 3D**) would instead be expected to slightly increase the pK_a_ of the thiol group [51]. It is possible that other hydrogen bonding interactions, *e*.*g*. the water-mediated hydrogen bond to Tyr163, as well as the concentration of basic amino acids toward the C-terminus of α5 (His127, His147, Arg160, and Arg135) might mitigate the unfavorable interaction between a cysteine thiolate and the helix dipole (**Fig 3D**).

However, what is evident is that the C-terminus of the α5, although still maintaining a helical structure, is the least stable area of the linker helix, being held in place through an extensive hydrogen bonding network around Cys128 that includes His147, Asp115, Arg160 and Asp164 (**S3C Fig**). In the event of Cys128 oxidation in this delicate region, one could envision it becoming less stable, allowing it to overcome a threshold change in free energy, thereby initiating RitR structural rearrangements that render Cys128 more accessible to form the oxidized dimeric active protein, or sulfinic/sulfonic forms. In support of this model, thermal shift data shown in **S3F Fig** reveals that although the RitR_C128S_ mutant appears to be less stable than the wild-type protein overall, a clear destabilizing effect can be seen following RitR oxidation of the wild-type protein, which is absent altogether in the RitR_C128S_ mutant.

In further support of our activation hypothesis, we have included an oxidized mimetic (RitR_C128D_) structure of RitR (**S3D Fig and Table 1**). The RitR_C128D_ structure was sufficiently similar to RitR_C128S_ to enable its solving via molecular replacement. Of note, the major difference between the RitR_C128S_ and RitR_C128D_ models was that no electron density was observed within the RitR_C128D_ peptide QHDSLMKV, where “D” would be the position of the wild-type C128 residue (magenta colored region in **S3D Fig**). Although the RitR_C128D_ structure might not be reflective of the actual oxidation state of RitR *in vivo*, the lack of electron density around the Cys128 Asp substitution does suggest that changes in oxidation at the Cys128 residue would be sufficient to destabilize the linker region, thereby initiating downstream structural rearrangements.

Aside from its unstructured Cys128 region, the RitR_C128D_ oxidized mimetic mutant maintains virtually identical hydrogen bonding and RD/DBD positioning to the RitR_C128S_ structure, with the two (RitR_C128S_ and RitR_C128D_) structures having an RMSD of 0.35 Å (219 of 221 Calphas; **S3D Fig,** compare colored and grey structures). Finally, the strategic positioning of Gly120, just N-terminal to the helical linker, could provide a vulnerable weakening point of this helix to more easily allow its unraveling during oxidation and activation of the protein. Indeed, in the oxidized form we also observe *in vivo* the absence of the linker peptide beginning with Gly120 (*i*.*e*. Peptide GRDFIDQHCSLMKVPR; **S2C Fig**), indicating in the activated conformation helix 5 was unraveled and thus accessible from Gly120 to Arg135.

Taken together, these data support a mechanism by which oxidation of Cys128 interferes with local hydrogen bonding, ultimately resulting in the release of the DBD from the REC domain, dimerization, and subsequent binding to DNA.

### Atomic structure of wild-type oxidized RitR (RitR_OX_)

**Fig 4:**
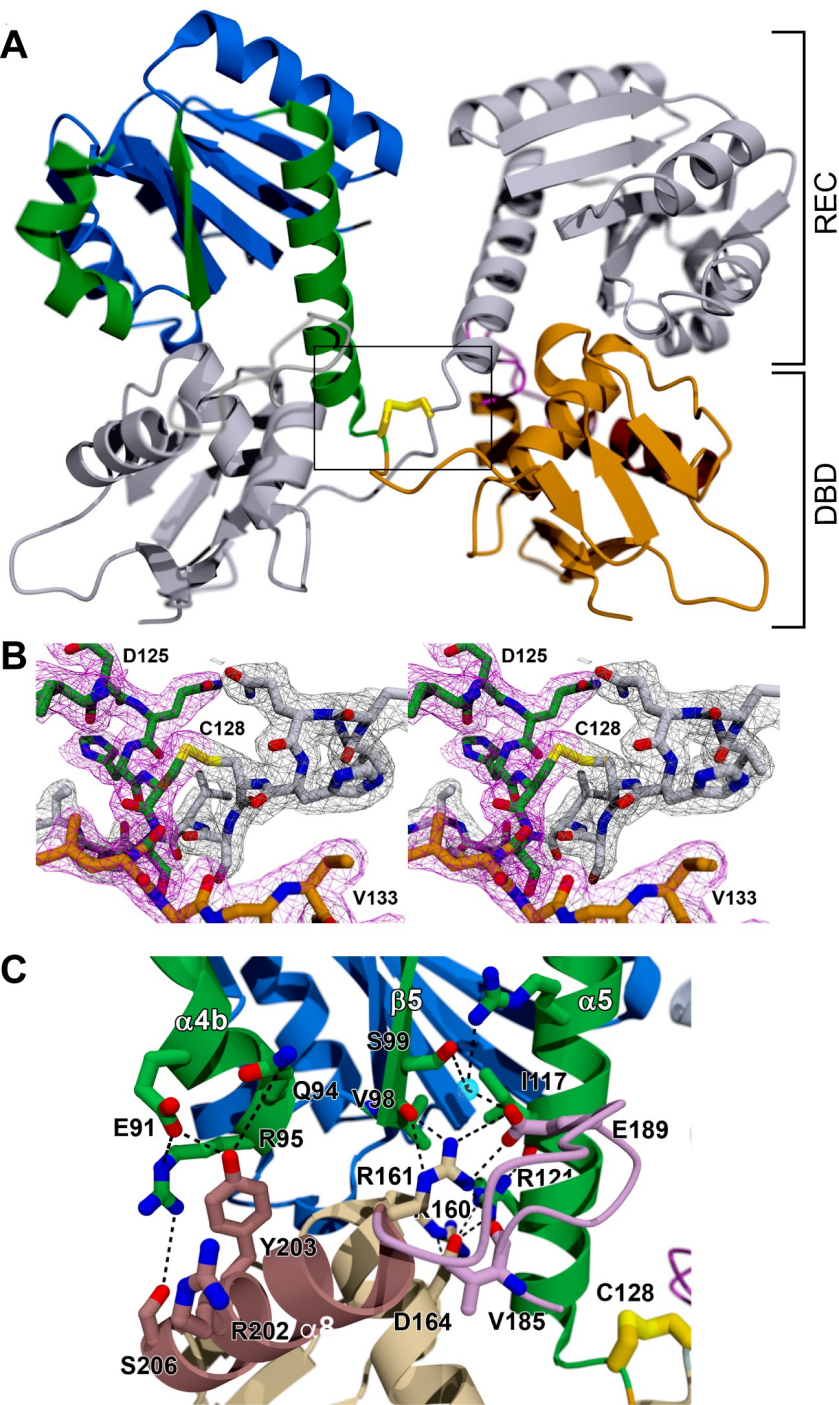
RitR oxidized structure. (**A**) Cartoon representation of the domain-swapped RitR_OX_ structure. One protomer is in color and the other protomer in grey. REC, receiver domain; DBD, DNA-binding domain. (**B**) 2|F_o_|-|F_c_| composite omit electron density for the inter-protomer Cys128:Cys128’ disulfide bond and surrounding residues that pins the C-terminal ends of each α5 helix together. As a consueqence, both DBDs are in close proximity. One protomer is shown in color with a pink density map, and the other protomer is shown in grey with a matching grey density map. (**C**) Image of the interface between the DBD of one protomer of the RitR_OX_ homodimer (bright colors) and the REC domain of the other protomer (muted colors). The interactions are almost identical to those observed for the C128S structure in Fig 3C.

To observe structural rearrangements after RitR oxidation at the atomic level, we purified, crystallized and solved the atomic structure of native wild-type RitR in the peroxide-induced oxidized, free dimeric form. Because the individual protomers of RitR_OX_ were sufficiently close to the RitR_C128S_ structure, the dimeric RitR_OX_ structure could be largely solved using molecular replacement (**Fig 4**; structural statistics can be found in **Table 1**). At least in the absence of its DNA target sequence, the oxidized RitR structure revealed that its individual domains do not change significantly upon oxidation with H_2_O_2_. However, the relationship between protomers changes dramatically, whereupon oxidation, as predicted, the DNA-binding domain is released from the α4-β5-α5 interface of the REC domain. In the absence of its interacting DNA, the final result of the free form of oxidized RitR produces an unusual domain-swapped structure, *i*.*e*. where the DNA binding domain from protomer 1 binds to the RD of protomer 2 and *vice versa* (**Fig 4A**).

Although such a domain-swapped structure at first appears artefactual, the literature tells us otherwise. Indeed, there are several examples of transcription factors which use one form or another of a domain swapping strategy to modulate transcription, in both eukaryotes and prokaryotes [52–54]. Whether or not such a configuration is important for RitR function remains to be deciphered. Nevertheless, we predict that the RitR oxidized structure shown here likely represents the free form of the oxidized protein before its interaction with DNA, or at the very least, one of its regulatory forms (*e*.*g*. alternate conformers brought about by Cys128 SO_2_/SO_3_ differing oxidized states).

Despite these large rearrangements, the RitR DBD helix 8 again binds to the α4-β5-α5 face of the second RitR molecule to form the conserved and inhibitory hydrogen bonding network similar to the inactive RitR_C128S_ structure (compare **Fig 4C** with **S6A Fig**). Indeed, when we examined residues predicted to interact with DNA (**S5 Fig**) in the RitR_OX_ structure we observed an almost identical profile to that of the inactive RitR_C128S_ structure interactions with the REC domain, with the exception of Arg202 (**S6A Fig**). When electrostatics of RitR_OX_ were compared to that of PhoB and the RitR_C128S_ structure a similar pattern also emerged (**S6B Fig**), suggesting that even when swapped the REC-DBD interactions are virtually identical. However, a comparison of average B-factors between the RitR_C128S_ and RitR_OX_ structures revealed that although REC-DBD interactions appear almost identical in their 3-dimensional atomic coordinates, there are major differences in their average residue B-factor values, where the RitR_C128S_ and RitR_OX_ structures exhibit average alpha carbon B-factors of 11.03 and 36.25, respectively.

Importantly, the RitR_OX_ swapped domain structure is possible due to the effective unwinding of the C-terminus of the linker (α5 helix) to free Cys128, enabling it to form the inter-protomer disulfide bridge, and unequivocally showing that Cys128 is responsible for dimer formation (**Fig 4B**). Aside from the Cys128-Cys128' linkage and the adjacent Ser129 forming a main chain nitrogen bond to the hydroxyl side chain of Ser129' in the other protomer, there are few observable electrostatic interactions between swapped dimers to hold the conformer together (**Figs 4A** and **S7C**, left panel). Indeed, the lack of RitR_OX_ inter-protomer hydrogen bonds are in line with the more than 3-fold greater average B-factor score in the oxidized structure in comparison to the (more overall stable) RitR_C128S_ structure.

To further explore this phenomenon, we wanted to compare regional instability within the individual RitR_C128A_ and RitR_OX_ structures, anticipating that any observed localized instability in the structures might indicate an *in vivo* functional consequence. Regional stability was calculated as the fold change in each residue’s alpha carbon B-factor score from the overall mean B-factor score of the structure. A graphical output of these results is displayed in **S7A Fig** revealing that, in general, both RitR_C128S_ and RitR_OX_ structures exhibit very similar regional instability in the REC domain. Interestingly, the loop and ensuing beta sheet (beta strands 6–7), which immediately follow the Cys128 coordinate, exhibit enhanced destabilization in the RitR_C128S_ monomer structure. Conversely, the RitR_OX_ average B-factor value shows surprising stability in this particular region (**S7A Fig**), where we see helix 8 unravel to allow for the REC-DBD domain swapping phenomenon. A more detailed look at the hydrogen bonding of the two structures in this area (residues Leu130-Gly150) reveals a Lys132-Val133-Pro134 motif that exhibits a dramatically different conformation when RitR_C128S_ and RitR_OX_ structures are compared. The alpha carbon of Pro134 demonstrates an approximate 180° rotation, going from largely unstable in the RitR_C128S_ reduced structure, to a far more stable conformer in the RitR_OX_ form. As compared to the rest of the RitR_OX_ protein, the greater stability of this region in RitR_OX_ structure might be attributed to the aforementioned additional hydrogen bonds formed by the Ser129 main chain nitrogen bond to the hydroxyl side chain of Ser129' in the other protomer, and a similar interaction between the main chain oxygen of His127 and main chain nitrogen Met131’ in the RitR_OX_ structure (**S7B**–**S7C Fig**).

Although significant, in terms of RitR mechanism the functional consequences of these observations are currently not understood. To speculate, one could envision the more stable complex in the oxidized state as a way to ensure a successful signaling event during oxidative stress experienced by the pneumococcus. Whether this regional stability and signaling would result in the formation of DNA-binding and *piu* repression, or another as of yet unidentified signaling function requires additional research.

### Conservation of RitR and Cys128

**Fig 5:**
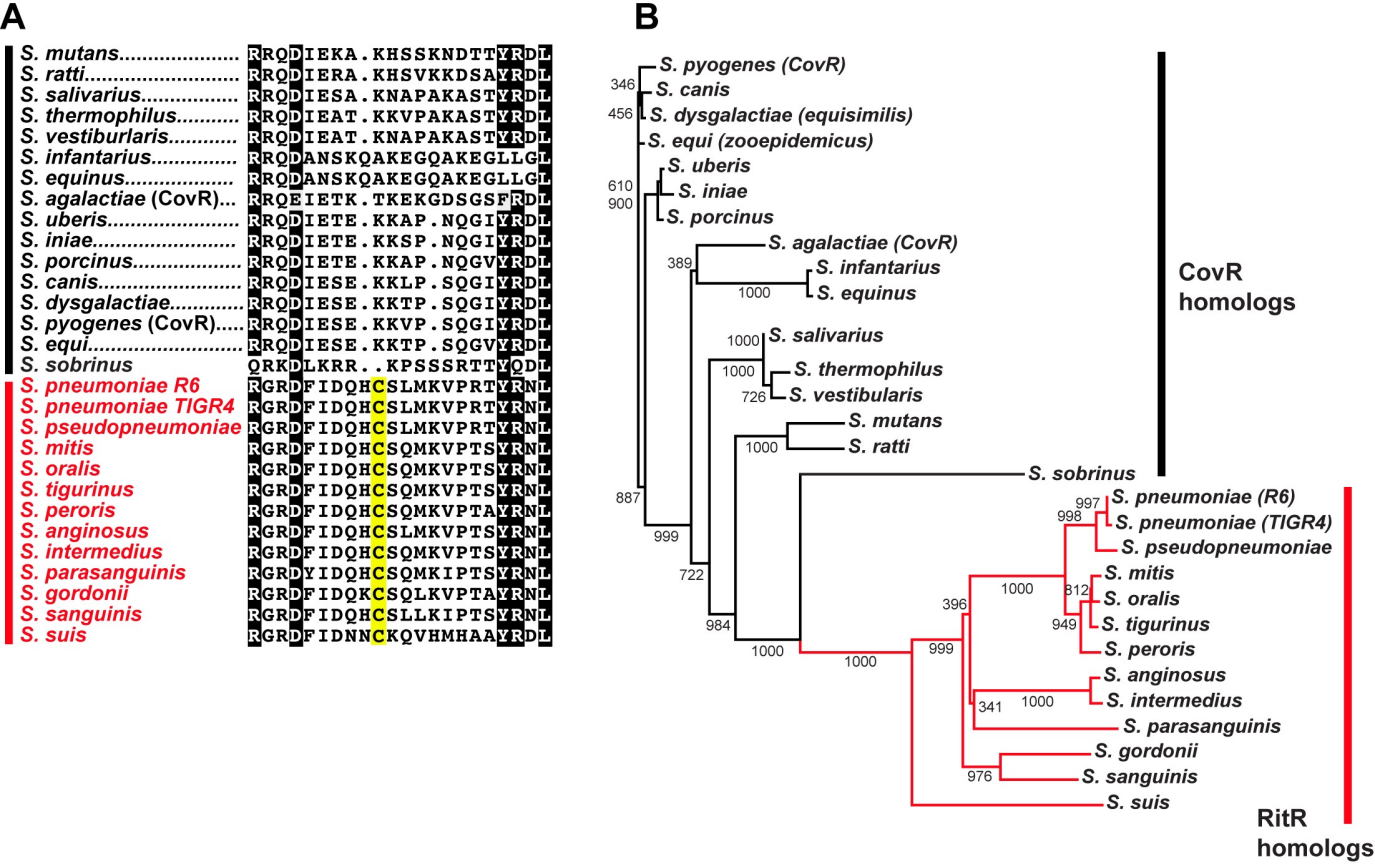
Conservation of RitR in the streptococci. (**A**) Alignment of linker regions of RitR homologs (in red) and CovR homologs (in black) from the streptococci. Notice the degeneracy in the “HCS” motif in the swine zoonotic pathogen *S*. *suis*. Identical residues are colored black and similar residues are colored grey. The conserved cysteine is shaded in yellow. (**B**) Phylogenetic tree of RitR (in red) and CovR (in black) streptococcal homologs. Evolutionary distance is depicted by the length of the horizontal lines. Posterior probabilities are displayed at the branch points.

As RitR is a novel redox sensor derived from two-component systems, we were interested in its broader conservation among the streptococci. Sequences were amassed from several streptococcal species, aligned, and a phylogenetic tree generated (**Fig 5)**. The alignment from the full-length response regulator sequences used in **Fig 5**is shown in **S4 Fig**.

Results clearly indicate a conservation of Cys128 in RitR homologs among a subset of streptococcal species which are largely representative of oral pathogens that are unable to perform aerobic respiration, including the well-described *S*. *mitis* and *S*. *oralis* species (**Fig 5A and 5B**). As a general rule, these species lack a complete tricarboxylic acid (TCA) cycle and produce high levels of H_2_O_2_ [55]. On the other hand, species which lack the conserved cysteine residue and are able to respire, *e*.*g*. *S*. *agalactiae* (Group B streptococci) and *S*. *pyogenes* (Group A streptococci) instead harbor the closest RitR hololog, CovR. Unlike the orphan regulator RitR, the virulence regulatory protein CovR is a typical two-component response regulator that has a histidine kinase cognate partner (CovS) and contains the canonical phosphorylatable aspartate residue [30, 56, 57]. RitR and CovR were found to produce distinct clades within the phylogenetic tree, suggesting a functional divergence that coincides with the lack of a complete TCA cycle and high H_2_O_2_ production [55] (**Fig 5**).

### Cysteine linker statistics

Nature rarely places cysteine residues within a given protein without a finite role in mind. In support, functional selective pressure ensures around a 90% rate of cysteine conservation in related protein sequences, which along with tryptophan distinguishes this reactive amino acid as having the highest conservation in nature [58, 59]. Keeping this in mind, because the RitR linker region length can ultimately determine the functional response of bacterial regulator proteins [60], we wanted to investigate both the general frequency and position with which cysteine residues appeared within linker regions between REC and effector DBDs. To answer this question, we first devised a custom program to define the linker region between all deposited C-terminal REC sequences and their associated N-terminal output DNA-binding domain effectors. In doing so several statistics were gathered, including: (i) linker length, (ii) the number of cysteines per linker, and (iii) the relative location of the cysteines within the defined linker region. As far as we are aware, *en masse* statistics of linker domains across all prokaryotic species (*e*.*g*. between a REC and output domain) have yet to be examined.

**Fig 6:**
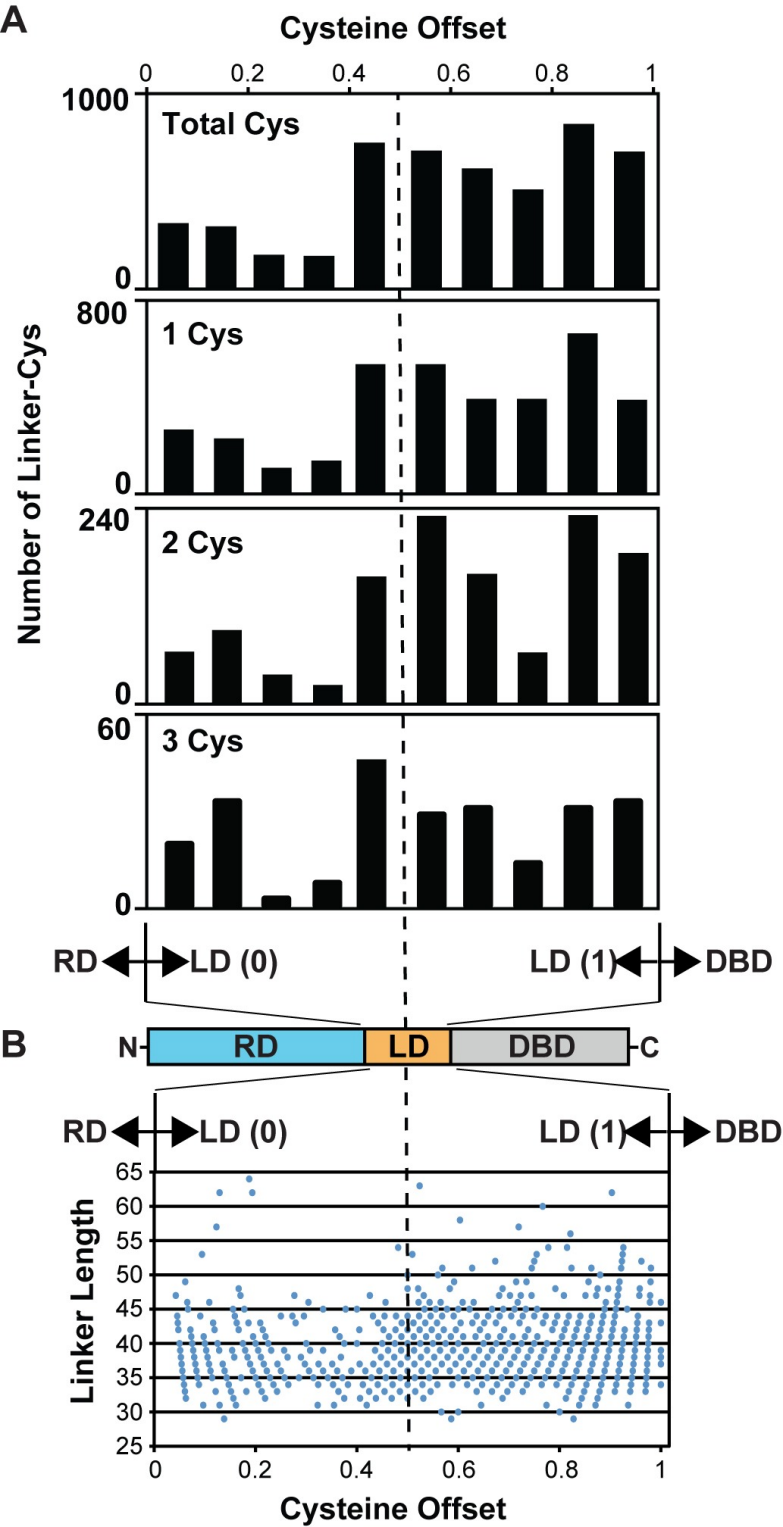
Informatics of linker length and cysteine placement in REC-DBD domain architectures. (**A**) Bar graphs showing the number of single, double, triple and total cysteines found within block regions of the defined linker domain. Notice the obvious weighting to the linker C-terminus, closer to the effector DNA-binding domains. (**B**) Linker length versus cysteine placement plot from sequences where the linker contains only one cysteine. RD, regulatory domain; LD; linker domain; DBD, DNA-binding domain. The x-axis “cysteine offset” refers to cysteine placement from the N-terminus on the predicted linker (designated as “0”) and the C-terminus of the linker (designated as “1”). The center (0.5) of the predicted linker domains are designated by the vertical dotted line.

Predicted cysteine-containing linker regions varied from 29 amino acids in length to as much as 86, however, by far the majority of defined linker sequences fell within the range of 34 to 44 amino acids in length (**S8A Fig**), with a mean length of 38.2 (standard deviation of 4.73). A total of 4,330 cysteine-containing linker domains were extracted, indicating that 28% of all linker domains contain at least one cysteine residue. The maximum number of linker cysteines was six, a number found in only one linker sequence. Of the total (4,330) amassed cysteine-containing linker sequences, 3,621 contain one cysteine, 623 two cysteines, 86 three cysteines and only three sequences four cysteines. From these studies the most surprising finding was that, paralleling RitR Cys128, there is a clear preference favoring cysteine positioning within the C-terminal end of the linker domain. This phenomenon held true for 1, 2, 3, or 4-mer cysteine-containing linker sequences (**Fig 6A**). Linker length with cysteine position (or the calculated “offset” value) relative to the calculated linker “center” are combined in **Fig 6B**. The data again demonstrate overwhelming favoritism for cysteine placement within the C-terminus of the linker domain, and interestingly are generally C-terminal irrespective of linker length.

Next we investigated if there were any commonalities in the amino acids surrounding the linker cysteines. Motifs were generated by compiling a list of total linker cysteine residue sequences, after which a logo output was generated (**S8B Fig**). These data revealed a very strong preference for N-terminal arginines in the -1, -2 or -3 position relative to the Cys, and a C-terminal glycine in the +1, +2 or +4 positions relative to the Cys (**S8B Fig**). An arginine is also preferred in the +4 position. Arginines are positively charged and are thus able to change the pKa of nearby cysteines to a more acidic pH, a hallmark of the “biologically important” cysteine biosensor [8, 61, 62]. Interestingly, glycines are not commonly found within helices unless flexibility is required (*e*.*g*. RitR Gly120).

To then further explore if the placement of the conserved arginines could interact with the Cys to influence its pKa, we constructed a model helix based on the most common residues, or consensus sequence, surrounding Cys-containing linkers (*i*.*e*. motif: RLR**C**GLLR). The resultant helical model shown in **S5C Fig** indicates that due to proximity on the same helical face, the linker Cys can indeed be influenced by Arg residues placed at the -3 and +4 positions (3–4.5 Å distance).

Collectively these results show an evolutionary selection for Arg residues that could influence the pKa of a helical linker cysteine residue [51], and glycines which would give flexibility within the helical linker sequences. Combined with the proximity of the majority of linker cysteines residing within the C-terminus of the helix (close to the effector (output) domain), these results suggest that the RitR mechanism of using oxidation of a linker helix to modulate protein activity could be a common mechanism of signaling regulation and warrants further investigation.

Finally, we wished to identify any phylogenetic trends in bacterial species harboring Cys-containing linker regions (see Methods for details). Results showed that most of the top 13 Classes of bacteria which harbor the highest percentage of Cys-linkers were either anaerobes, such as the Chlorobia or Clostridia species, or photosynthetic bacteria, such as Gloeobacteria and Cyanobacteria (**S8D Fig**). These findings are not surprising, as one would expect organisms that cannot survive under oxygen-rich conditions, or conversely rely on oxygen for energy (photosynthesis), to possess heightened levels of biologically (non-structural) active cysteines. These cysteines could be especially important within signaling proteins, enabling such microorganisms the ability to detect changing environmental oxygen content.

## Discussion

### The pneumococcal iron paradox

Redox-sensing transcription factors have been well described, where they play critical roles in sensing and responding to both internal and external ROS threats. For bacteria, several classes have now been revealed (for review see Faulkner and Helmann [12]). *S*. *pneumoniae* and related microbes produce millimolar quantities of hydrogen peroxide as a metabolic byproduct of SpxB. Despite this function, these high H_2_O_2_-producing streptococci are surprisingly still able to maintain intracellular ferrous iron levels comparable to that of *E*. *coli* [14], and could be the reason why in serotype 4 a *ritR* deletion appears to be lethal [39]. Indeed, by most standards these levels of iron and peroxide should be a lethal combination in *S*. *pneumoniae* from the resultant Fenton chemistry [2]. Recently, a thiol peroxidase (TpxD) and putative glutathione peroxidase (Gpx) have been largely implicated in this H_2_O_2_ related stress adaptation [17], however it has remained a mystery as to how *S*. *pneumoniae* and many related oral and respiratory tract pathogens are able to regulate the production and intake of these hazardous components in the absence of any known peroxide sensing transcription factors [13].

In more recent years there have been several descriptions of transcriptional regulators that influence pneumococcal peroxide resistance, including the MerR family regulator NmlR, which was shown to influence H_2_O_2_ expression in *S*. *pneumoniae* [24]. Yesilkaya and colleagues have identified the transcription factor Rgg that is required for resistance to paraquat but not H_2_O_2_ [22]. However, to date a *bona fide* peroxide-sensing transcription factor in the high H_2_O_2_-producing streptococci has remained at large [13]. Here we describe RitR as the first such redox sensor in *S*. *pneumoniae* and related streptococci, which we predict has evolved from the important (more canonical) streptococcal two-component virulence determinant CovR [57].

**Fig 7:**
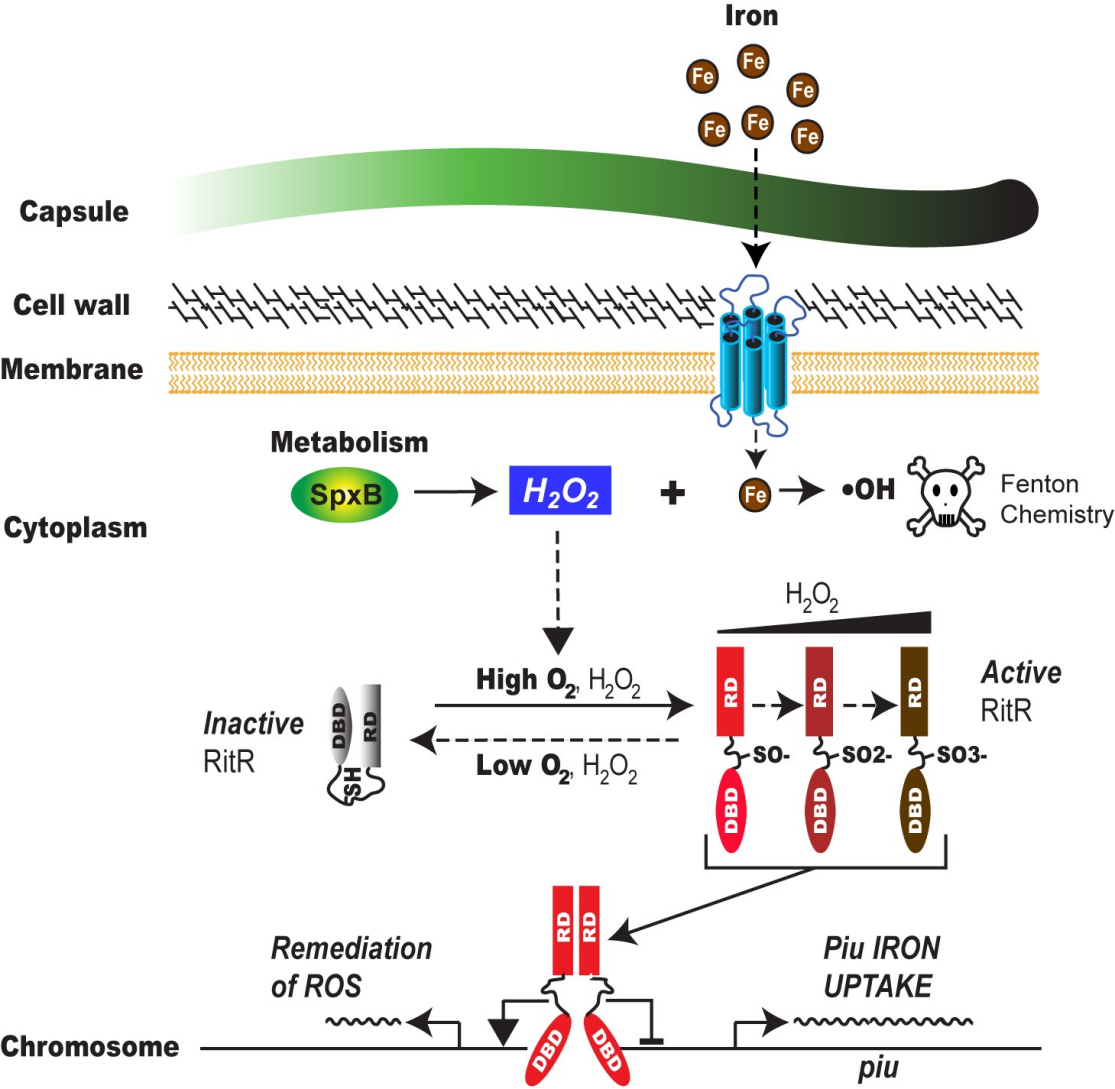
Schematic representation of RitR regulation in *S*. *pneumoniae*. Hydrogen peroxide (H_2_O_2_) is primarily produced from *S*. *pneumoniae* metabolism via pyruvate oxidase (SpxB). When present, iron must be kept out of the cell, or alternatively, stored such that it cannot react to yield Fenton chemistry, thereby causing cellular damage. RitR regulates this process by oxidation though Cys128 in high H_2_O_2_ produced in aerobic environments such as the nasopharynx, which allows its open conformation and release of the DNA-binding domain (DBD) for the Regulatory Domain (RD) to interact with the Piu promoter, repressing iron uptake. Simultaneously, RitR is postulated to remediate iron toxicity through activation of DNA repair and iron sequestration [25]. Conversely, when H_2_O_2_ concentrations are low, RitR stays in its inactive form, where the interaction of the RD with the DBD prevents its binding to the Piu regulatory region, ultimately allowing for more iron to enter the cell. The potential oxidation states of RitR (SO^-^, SO^2-^, SO^3-^) and their regulatory consequences remain enigmatic.

**Fig 7** presents a schematic representation of the proposed mechanism of RitR, where it is activated in the presence of high peroxide concentrations to repress iron uptake and remediate its toxic effects, thereby avoiding lethal Fenton chemistry. This model is supported by data showing that Cys128 is oxidized in aerated cultures and acts to repress Piu-mediated iron uptake. Data from the colonization model further support this model where we observe that the ΔritR, C128A and C128S mutants are severely attenuated in their ability to colonize the upper respiratory tract. Most strikingly, we see the C128D phosphomimetic completely alleviating this effect, lending further support to the notion that keeping intracellular iron concentrations low when the pneumococcus is producing high levels of peroxide in the airways is of benefit to its survival. Interestingly, a study by Weiser and colleagues identified human siderocalin, a siderophore binding protein, as the most highly upregulated transcript during pneumococcal nasopharyngeal colonization. This host strategy was postulated to be a reason why species such as *S*. *pneumoniae* that require little iron and do not produce nor rely on siderophores might be so successful at colonizing respiratory tract mucosal surfaces, which contain extremely low levels of accessible iron [63]. Nevertheless, in this environment *S*. *pneumoniae* still likely requires some iron to colonize, and must therefore maintain strict intracellular levels of this element to avoid Fenton chemistry with its high SpxB activity. In further support of this hypothesis, RitR does not seem to be required for infection in low free oxygen environments like the blood [25, 27].

### Comparison of RitR to other known redox-sensing transcription factors

Several cysteine-utilizing redox regulators have been described in prokaryotes [6]. Some use cofactors, such as the peroxide sensor PerR that incorporates a metal center in its structure [10], and is present in many streptococcal species (*e*.*g*. *S*. *pyogenes*, *S*. *mutans* and *S*. *agalactiae*), but notably absent in RitR-containing species which tend to lack a complete TCA cycle [13]. PerR possesses a unique mechanism whereby a hydroxyl radical is generated via H_2_O_2_ and ferrous iron (Fe^2+^), whose action directly inactivates the protein's molecular switch. Hypothesizing that RitR might have a similar mechanism, we did attempt to add several divalent metal ions and assess RitR dimerization. Only CuCl_2_ appeared to aid in dimerization when H_2_O_2_ was also added, however CuCl_2_ did not assist in binding to DNA, which was initiated easily in the presence of peroxide itself (**Figs 2D** and **S10**)

Although the mechanism of RitR activation is quite unique in its own regard, intermolecular disulfide bridging as a means to dimerization is not a new concept in activation of redox sensors. Examples from bacterial transcription factors that use an intermolecular bridging mechanism include some OhrR homologs that contain two cysteines [64, 65], and CprK from *Desulfitobacterium dehalogenans* [66]. However, as previously discussed, when compared to these and other available oxidized structures, RitR experiences considerable atomic rearrangements, where the N-terminus of the α5 helix becomes unraveled. Although we do not know what the actual oxidized state looks like when bound to DNA, because Cys128 is found buried within the RitR structure in the reduced state, at a minimum the α5 must unravel itself and the DBD disengage from the ALR (REC) domain to facilitate binding and repression of Piu transcription (shown in **Fig 4**). Because our RitR oxidized structure takes on a similar configuration, but domain-swapped, as compared to the reduce state, it is interesting to speculate that such a structure might exist *in vivo* to serve an as-of-yet unknown regulatory function. For example, this reversible state could occur under moderate peroxide stress where RitR would still be unable to bind DNA and repress *piu* transcription. Although this dimeric domain-swapped form might be “one step closer” to activation, under high peroxide stress an over-oxidized SO^2-^ and/or SO^3-^ Cys128, now unable to dimerize through the disulfide bridge, might then shift inactive to active equilibrium such that repression could more easily occur. Future studies exploring the *in vivo* and *in vitro* consequences of Cys128 oxidation would be required to fully answer these interesting questions.

Another parallel of the RitR mechanism of activation with other redox-sensing transcription factors is its more acidic pKa of Cys128 (pKa 6.85) as compared to cysteine in its free form (pKa 8.5) (**S3E Fig**). A pKa of 6.85 is well in line with other redox-sensing cysteines (*e*.*g*. the redox regulator HypR that has a reactive cysteine pKa of 6.3 [62]). Nevertheless, this pKa is on the higher end of the typical redox-sensing spectrum, which would make it more difficult to oxidize and thus require a higher threshold of peroxide concentrations to activate. However, if we contemplate the high oxygen lifestyle of the pneumococcus this thinking makes perfect sense. *S*. *pneumoniae* and related species have the potential to generate extremely high (mM) concentrations H_2_O_2_, largely due to their SpxB activity, and are therefore much more resistant than most bacterial species to this oxidant. It would therefore serve this pathogen well to activate a peroxide stress response at a much higher threshold than other more peroxide susceptible bacteria.

Often the residues surrounding reactive cysteines are positively-charged (*e*.*g*. arginines, lysines and histidines) where they contribute to the regional stability of the thiol (*e*.*g*. in the case of OxyR; [67]), or can also take the form of other residues such as tyrosine (*e*.*g*. in the case of OhrR; [8]). However, in the case of RitR, residues contributing to the more acidic pKa were not as obvious, but could include nearby Tyr163 that forms a bridge to Cys128 through a water molecule (**Fig 3D**). Indeed, although it was the first redox sensor to be described, the mechanism of OxyR peroxide-sensing has now been determined to involve the replacement of water in the active pocket with H_2_O_2_, then triggering the redox chemistry which ultimately results in major protein rearrangements and DNA binding [36]. Future structural and biochemical work will have to determine if RitR experiences a similar detailed mechanism of activation.

One perplexing result of these studies was the complete complementation of the Piu repressive phenotype with the C128D mutant. In this construct, RitR is “activated” but obviously cannot facilitate a disulfide bridge. We therefore propose the following hypothesis as to how RitR could become active in the absence of disulfide formation. First, we know that initially Cys128 must oxidize in the presence of increasing peroxide concentration to the sulfenic (SO^-^) form, where it could then either form a reversible disulfide bridge with the an adjacent oxidized (SO^-^) monomer, or if sufficient oxidant is present further oxidize to the irreversible sulfinic (SO^2-^) and sulfonic (SO^3-^) forms [6]. In the latter two cases, a disulfide bridge would obviously not be possible, yet this appears to be the actual activated state of the protein *in vivo* as depicted by our C128D data. Indeed, with two oxygens in its structure, an aspartate substitution would more mimic the sulfinic state of Cys128 rather than the sulfenic. Therefore, in such a model the proposed active sufinic state of Cys128 would only initiate structural rearrangements to facilitate dimerization and Piu repression, rather than being a requirement to hold the dimeric state together to function properly. So-called “over-oxidized” cysteine states have now been implicated in protein signaling specific to their varying oxidation states, and are thought to be highly underappreciated in the signaling world [6, 68]. In the future, careful biochemical analyses will be required to understand the precise mechanism by which RitR and its varying oxidized states contribute to its function.

### The evolution of a two-component response regulator to a redox sensor

The closest canonical two-component response regulator homolog of RitR is CovR (**Fig 5**). CovR has been previously shown to control virulence determinants in several streptococcal species, including both Type A and Type B streptococci [57]. Differing from the classic phosphorylatable REC domain of CovR that is controlled by its cognate histidine kinase CovS, RitR instead possesses an Aspartate-Less Receiver (ALR) domain, which by definition lacks the phosphorylatable aspartate residue required for histidine kinase phospho-relay [30–32, 69]. Here we show that RitR has instead evolved away from histidine kinase activation to essentially become independently activated through dimerization in the presence of the specific H_2_O_2_ oxidant.

This transition from a canonical phospho-relay protein to cysteine-activated redox sensor is most evident from key residues missing in the RitR sequence that typically aid in REC-mediated dimerization (**Fig 2B**). In support of this theory, we show here that when canonical REC dimerization residues are “re-introduced” into the RitR protein, RitR can then form a significant amount of dimer in the absence of peroxide (**Fig 2C**). The absence of these key residues involved in typical two-component dimerization, especially the two aspartic acid residues within the conserved “GADDY” sequence, are likely responsible for the dominant monomeric form of RitR in solution. This theory is supported by our SEC experiments (**Fig 2**) and full-length RitR_C128S_ monomer crystal structure (**Fig 3**). Conservation of the dimerization residues, Cys128, RitR’s orphan status and its known co-transcription with the pentose phosphate pathway enzyme 6-phosphogluconate dehydrogenase are its signature identifying traits [25].

One RitR homolog with these distinguishing characteristics has been previously studied, but possibly mistaken for a CovR homolog. The study comes from Pan *et al*. where they have published a comprehensive work on an orphan response regulator identified in the swine pathogen *S*. *suis*. This version exemplifies all of the aforementioned hallmarks of *S*. *pneumoniae* RitR, including the conserved cysteine (**Fig 5A**) and shared synteny with the *S*. *pneumoniae gnd-ritR* operon [70]. However, when the *S*. *suis* RitR was deleted several phenotypic and regulatory differences to our pneumococcal studies are evident, including production of a thicker capsule, observation of longer chain lengths suggestive of cell division problems, and enhanced virulence/lethality (*e*.*g*. haemolysis activity). Of note, the authors performed microarrays to determine gene regulation and found that the capsule biosynthesis RNA levels and major virulence determinants were affected, rather than genes which would be suggestive of a RitR-type profile such as iron and oxidative stress related operons [70]. In fact, the *S*. *suis* RitR data more parallels gene regulatory profiles of CovR, thus fitting a profile of a virulence repressor, rather than a RitR-type redox regulator. An examination of the CovR/RitR phylogenetic tree shown in **Fig 5** predicts that the *S*. *suis* RitR homolog was the first to diverge from its CovR ancestor. Combined with the fact that *S*. *suis* is a human zoonotic pathogen [71], it is interesting to speculate that RitR from *S*. *suis* could have been transferred to *S*. *pneumoniae* and other human-specific pathogens during human swine domestication. Emergence of new human disease as a result of animal domestication is not unprecedented. For example, the related pathogen *S*. *agalactiae* was only considered a pathogen of domesticated animals until the first report of human *S*. *agalactiae* sepsis in 1964. This recent emergence within the human population has now largely been attributed to the domestication of animals [72]. Expanded genome sequencing and an examination of *S*. *suis* RitR function will have to be explored to further determine the merits of this theory.

### Cysteine residues in linker regions–a conserved mechanism of redox sensing?

We are not aware of any studies which have thoroughly defined linker regions in prokaryotic signaling proteins, nor any that have identified trends in conserved residues that could be tied to function. As cysteine residues are often strategically placed in proteins to assist in tertiary structure folding, catalysis in proteases and peptidases, or as in the case of Cys128, placed for the purpose of detecting cellular redox states [58], we created a program to identify cysteine residues within linker regions between all deposited REC and DNA-binding domains. It was surprising to find that, similar to RitR Cys128, most cysteine residues reside closer to the output domain (DBD) of REC-containing prokaryotic transcription factors (**Fig 6**). Although it is difficult at this stage to assess the meaning of these data, it is nevertheless clear that the overwhelming majority of REC-DBD signaling proteins have evolved to place cysteine residues in this region, which suggests structural/functional conservation. In further support of our findings that the majority of identified linker cysteines could be redox-sensing, the most common adjacent amino acids sharing the same helical linker face were found to be arginines, which like in the case of OxyR could aid in the stabilization of thiolate anions [67] [73]. Another interesting observation was the weighting of linker-containing cysteines towards microorganisms that prefer or exclusively reside in an anaerobic environment (**S7D Fig**). These data make sense as one could imagine the requirement for more proteins, and especially signaling proteins, having the capability to detect small changes within the cellular redox equilibrium which could result in cell damage or even death for these niche-adapted organisms. However, whether this phenomenon is due to the fact that anaerobes contain sure greater numbers of cysteine residues, or if these linker-cysteines were strategically placed to perform specific biochemical sensory functions, remains to be deciphered.

## Materials and methods

### Strains and growth conditions

Bacterial strains used in these studies are given in **S1 Table**. Cultures of pneumococcus were grown overnight from frozen (-80°C) stocks in CAT medium (0.5% (w/v) tryptone, 1% (w/v) casein digest, 0.1% (w/v) yeast extract, and 5 mg/L choline) containing 0.02% glucose and the appropriate antibiotics at 37°C in a humidified 5% CO_2_ incubator. The following day cultures were then diluted 1:10 in Todd-Hewitt broth medium (Becton Dickinson) supplemented with 0.5% (w/v) yeast extract (THY) containing appropriate antibiotics, and the cell density measured periodically at 600 nm using a Biomate 3 spectrophotometer (Thermo Scientific, Waltham, MA). Standard “microaerophilic” growth was performed under static conditions in a CO_2_ chamber, whereas aerobic growth was performed in a standard air shaker (shaking at 200 rpm). For anaerobic growth an anaerobic chamber was used (Don Whitley Scientific, Shipley, UK) with 10% CO_2_, 10% H_2_ and 80% N_2_ settings.

Pneumococcal cultures were transformed using Competence Stimulating Peptide 1 (CSP-1), a generous gift from Donald Morrison, University of Illinois at Chicago. For transformations, *S*. *pneumoniae* cells were inoculated from frozen stocks into THY broth and cultured to early exponential phase (*i*.*e*. an OD_600_ of 0.01–0.03). At this time, CSP-1 was added to a final concentration of 200 ng/ml and the culture was incubated for 14 minutes before adding 100–200 ng of DNA. The transformation reactions were then placed back into the CO_2_ chamber and incubated between 45 minutes and 2 hours before being plated onto Tryptic Soy Blood Agar (TSBA) plates (Becton Dickinson, Oxford, UK) containing 5% defibrinated sheep blood (Rockland Immunochemicals, Gilbertsville, PA) with appropriate antibiotics and grown at 37°C under 5% CO_2_ until resistant colonies appeared. The following antibiotic concentrations were used for *S. pneumoniae*: 200 μg/ml streptomycin (Sm), 500 μg/ml kanamycin (Kan), 100 μg/ml spectinomycin (Spec), 3 μg/ml tetracycline (Tet). Strains of *E*. *coli* were cultured for plasmid purification overnight with aeration in a 37°C incubator in Luria-Bertani (LB) medium supplemented with appropriate antibiotics: either 50 μg/ml spectinomycin, 34 μg/ml chloramphenicol (Cam), 100 μg/ml ampicillin (Amp) or the more stable Amp substitute, carbenicillin (Carb) at 50 μg/ml.

### Construction of strains and plasmids

For pulldown experiments, the *ritR* mutant described in in Maul *et al*. [30] was used and FLAG-tagged RitR variants were expressed using the replicating plasmid pNE1 [74]. For all other experiments, markerless mutations were introduced into the chromosome of both the unencapsulated (rough) *S*. *pneumoniae* R800 strain [75] and encapsulated D39 (smooth, serotype 2) backgrounds using the Janus system (for details see Sung *et al*. [76]). First, D39 and R800 were transformed with a PCR product consisting of the *rpsL*_L56T_ gene with 724 and 727 base-pairs (bps) up and downstream respectively, followed by selection with streptomycin. The resulting streptomycin-resistant D39 *rpsL* and R800 *rpsL* strains were then used for the construction of the *ritR* variants. PCR constructs for transformation were constructed by overlap-extension PCR. To introduce the *kan-rpsL*^*+*^ cassette into the *ritR* locus, a construct was created consisting of 3 fused products: (1) a region 752 bps immediately upstream of the ATG of *ritR* amplified from D39 genomic DNA using primer pair *ritR lift F/ritR-J up R1*, (2) the Janus cassette, a kind gift from Christophe Grangeasse, Institut de Biologie et Chimie des Protéines, France, amplified from a PCR product using primer pair *ritR-J F/ritR-J R*, and (3) a region 761 bp immediately downstream of the stop codon of *ritR*, amplified from genomic DNA using primer pair *ritR-J down F1/ritR lift* (S1 Table). The final product was transformed into both D39 and R800 to create D39 and R800 *ritR*::*kan-rpsL*^*+*^ strains. To create the *ritR* variants, 2 PCR products were fused. For D39 and R800 *ΔritR* strains, the *kan-rpsL*^*+*^ cassette was replaced with a PCR product consisting of the 752 bp immediately upstream of the ATG of *ritR*, the first and last six codons of *ritR* and the 761 bp immediately downstream of *ritR* using primer pairs *ritR lift F/ritR-J up R2* and *ritR-J down F2/ritR lift R*, respectively (**S1 Table**).

The strategy of creating a *ritR* deletion (*ΔritR*), and then introducing chromosomal corrected mutants of Cys128, whereby ritR variants were re-introduced into the natural chromosomal position, was done to reconstruct the 6-phosphogluconate dehydrogenase *gnd-ritR* operon and eliminate any unwanted metabolic perturbations through *gnd* RNA destabilization. Thus, the only difference between the mutant and wild-type strains were the introduced Cys128 point mutations. The strains were created in two different parent genetic backgrounds: the rough (unencapsulated) *S*. *pneumoniae* strain R800 [75], and its encapsulated parent strain D39 [77]. The six genetic variants used in these studies are as follows: (1) wild-type (R800 or D39) (2) *ritR* chromosome deletion mutant ΔritR, (3) *ritR* wild-type chromosome corrected strain *ΔritR*::*ritR* WT, (4) *ritR* C128A sensory-dead corrected mutant *ΔritR*::*ritR* C128A, (5) *ritR* C128S reduced mimetic corrected mutant *ΔritR*::*ritR* C128S, and (6) *ritR* C128D oxidized mimetic corrected mutant *ΔritR*::*ritR* C128D. These strains are referred to in the text as wild-type (WT), WT complement, C128A, C128S, and C128D, respectively.

To create the *ritR* C128A/S/D variants, the following primer pairs were used to amplify and fuse fragments containing *ritR* with the desired mutation, and up and downstream regions: *ritR lift F*/*ritR-J C128A R* and *ritR-J C128A F*/*ritR lift R* (for C128A), *ritR lift F*/*ritR-J C128S R* and *ritR-J C128AS F*/*ritR lift R* (for C128S), and *ritR lift F*/*ritR-J C128D R* and *ritR-J C128D F*/*ritR lift R* (for C128D) (S1 Table). To replace the *kan-rpsL*^*+*^ cassette with *ritR* WT, the intermediate strain was transformed with a PCR product amplified using primer pair *ritR lift F/ritR lift R*. Correct integration and sequence was confirmed by PCR using primer pair *ritR check F/ritR check R*, followed by DNA sequencing.

Piu LacZ reporter strains were constructed as described in Maul *et al*. [30] using plasmid pPP2 [78]. Briefly an 849 bp fragment representing the upstream region of the Piu operon encompassing RitR binding sites 1–3 (-762 to +87) [25] and the native RBS were inserted into the *EcoR*I an *BamH*I sites of the pPP2 vector to create pPP2 P_*piu1-3*_. The Piu regulatory region was amplified for use in a restriction-less cloning method as previously described [79] using primer combinations *Ppiu-F1*/ *Ppiu-R2* and *Ppiu-F2*/ *Ppiu R1* (**S1 Table**). After confirming inserts by DNA sequencing, the pPP2 P_*piu1-3*_ plasmid was transformed into the *ritR* variants and correct integration (replacement of the native *S*. *pneumoniae bgaA* (β-galactosidase) gene) was confirmed by PCR using primer pairs *pPP2-tet-F*/*pPP2-tet-R* and *pPP2-bga-F*/*pPP2-bga-R* (**S1 Table**).

### β-galactosidase assays

To measure β-galactosidase activity, *S. pneumoniae* LacZ reporter strains were first grown to mid-exponential phase (OD_600_ 0.3–0.5). Briefly, a 20 μl aliquot of liquid culture was added to 80 μl of 100 mM sodium phosphate, 0.33% Triton X-100 (v/v) and then incubated at 37°C for 10 minutes. Reactions were started by the addition of 600 μl of 60 mM disodium phosphate, 40 mM monosodium phosphate, 10 mM potassium chloride, 1 mM magnesium sulfate, 50 mM β-mercaptoethanol, 1 mg/ml O-nitrophenol-β-galactopyranoside and allowed to run for 20 minutes at 37°C. Reactions were terminated by the addition of 700 μl of 1 M sodium carbonate and then clarified at 14,000 × g for 3 minutes. Absorbance of the supernatant was measured at OD_420_ using a Biomate 3 spectrophotometer (Thermo Scientific). Miller units were then calculated using the following equation: 1000 × [(OD_420_)/(OD_600_ of cultured sample) × (volume of sample in milliliters) × (reaction time in minutes)].

### Western blot analysis

For western blot analysis, protein extracts were separated by SDS-PAGE, and then transferred to PVDF membranes (EMD Millipore). Rabbit-raised antisera against PiuA and PiaA were originally prepared by CovalAb Inc. (UK) and were generous gifts from Dr. Jeremy Brown, University College London, UK. Anti-PiuA and anti-PiaA IgG were purified using a Protein A column as described in ref. [80]. Murine anti-NanA IgG was similarly generated and also a kind gift from Dr. Jeremy Brown. The RitR rabbit antisera was prepared by Covance Inc. (Princeton, New Jersey, USA) by immunization of 6–8 week old specific pathogen-free rabbits three times at three weekly intervals with purified recombinant RitR protein. Before use, RitR rabbit antiserum was affinity purified using purified RitR protein conjugated to a 1 ml HiTrap NHS-activated HP column (GE Healthcare Life Sciences) and specificity of the resulting immunoglobulin was verified by western blot of protein extracts from wild-type and ΔritR strains. The above antibodies were used at a dilution factor of 1:5000. To probe for FLAG-tagged proteins, an M2 mouse anti-FLAG monoclonal antibody (Sigma-Aldrich) was used at a dilution of 1:10,000. Proteins of interest were detected using HRP-conjugated goat anti-mouse IgG and goat anti-rabbit IgG (1:5000, Jackson ImmunoResearch) and visualized with Pierce ECL Western Blotting substrate (Thermo Scientific) and Amersham Hyperfilm ECL (GE Healthcare Lifesciences).

### Protein expression strain construction

A His_6_-SUMO fusion expression vector (LifeSensors Inc., Malvern, PA) was used to express large quantities of RitR for Size Exclusion Chromatography (SEC) and crystallographic experiments. RitR DNA from *S*. *pneumoniae* strain R800 [75] was amplified and cloned into the *Bsa*I site of the vector pE-SUMO to generate the N-terminal His_6_-SUMO fusion to the RitR protein by a restriction-less cloning method [79]. Two separate PCR reactions were run to generate *Bsa*I cohesive ends of RitR using primer pairs *RitR REC F1*/*RitR FL R2* and *RitR REC F2*/*RitR FL R1* (**S1 Table**). The two reactions were then combined and purified with QIAQuick PCR purification columns (Qiagen, Valencia, CA), and eluted in 40 μl of ddH_2_O. Using a thermocycler, the combined insert DNA was melted at 98°C and slowly reannealed to generate the *Bsa*I cohesive ends. Next, the RitR DNA products were phosphorylated using polynucleotide kinase (Promega, Madison, WI) in a reaction supplemented 1 mM ATP at 37°C for 4 hours. The phosphorylated products were then cleaned again using QIAQuick PCR purification columns, and then ligated into pE-SUMO cut with *Bsa*I using T4 DNA ligase and LigaFast rapid DNA ligation buffer (Promega, Madison, WI) for 15 minutes at room temperature. Ligation reactions were then transformed into *E*. *coli* DH5α chemically competent cells and plated on selective media LB medium supplemented with 50 μg/ml Kan. After sequence verification of the correct inserts, to generate the final protein expression strains the resultant plasmids were transformed into BL21(DE3), or BL21 Star (DE3) *E*. *coli* cells (Invitrogen, Carlsbad, CA). RitR mutations were constructed using the QuickChange method as described above using the appropriate primers (**S1 Table**).

### Expression and purification of RitR and oxidized RitR (RitR_OX_)

RitR expression and purification for EMSA and SEC experiments was carried out as described by Maule *et al*. [30]. RitR preparations for crystallography were carried out as follows. The His_6_-tagged SUMO-RitR fusion proteins (RitR wild-type, C128S, C128D) were expressed from *E*. *coli* BL21 Star (DE3) cells (Invitrogen Inc, Carlsbad, CA) carrying the pE-SUMO-RitR plasmid. Cultures were grown at 37°C in LB medium supplemented with 50 μg/mL Kan. When the cultures reached an OD_600_ of ~1.0, protein expression was induced with 0.4 mM IPTG. The temperature was then reduced to 20°C and the cultures were grown overnight with shaking at 250 rpm. The next morning, cells were harvested by centrifugation, resuspended in 5 ml per gram of cells in buffer A (25 mM TRIS pH 8.0, 300 mM NaCl, 10 mM imidazole) supplemented with 0.1 mg/ml of DNAse I (Worthington Biochemical Corp., Lakewood, NJ), and then lysed using a Branson Sonifier S-450 cell disruptor (Branson Ultrasonics Corp., Danbury, CT) for a total of 10 minutes of sonication at 50% amplitude with 30 second pulses, separated by 50 second rest periods. The temperature was maintained at or below 4°C by suspending the steel beaker in an ice bath directly over a spinning stir bar. The lysate then was clarified by centrifugation at 39,000 × g for 45 minutes and then applied to a 5 ml HisTrap column (GE Lifesciences, Piscataway, NJ) at a flow rate of 5 ml/min to isolate the His_6_-SUMO-RitR fusion protein. Protein was eluted by a 4-step gradient of 5, 15, 50, and 100% with buffer B (25 mM TRIS pH 8.0, 300 mM NaCl, and 250 mM imidazole). The His_6_-SUMO-RitR fusion protein eluted in the third step and was ~90% pure, as judged by Coomassie-stained SDS-PAGE. Peak fractions were pooled and dialyzed overnight against 3.5 L of 25 mM TRIS pH 8.0, 150 mM NaCl, and ~3 μM SUMO protease (LifeSensors Inc.). The dialysate was then passed through the HisTrap column a second time to remove the cleaved His_6_-SUMO tag as well as the protease. The resulting RitR preparation was > 95% pure. The protein preparation was then desalted using a HiTrap desalting column (GE Healthcare Life Sciences) into pH 6.5, 25 mM TRIS, 150 mM NaCl, 100 mM malic acid buffer and then stored at -80°C. The RitR_C128S_ and RitR_C128D_ proteins were purified using the same protocol as the wild- type protein.

RitR_OX_ protein was prepared by treatment with 1 mM hydrogen peroxide (H_2_O_2_), followed by size exclusion chromatography to obtain the oxidized dimer. The dimerized (oxidized) RitR_OX_ peak was purified from the remaining monomer using a HiPrep 26/60 Sephacryl S100 HR column at a flow rate of 1 ml/min. The running buffer used was 25 mM TRIS, 150 mM NaCl, 100 mM malic acid, pH 6.5, supplemented with 1 mM H_2_O_2_. The remaining procedures paralleled that of the other RitR preparations.

### Size exclusion chromatography (SEC)

SEC experiments were carried out using an Agilent 1220 Compact HPLC equipped with a 250 × 4.6 mm BioBasic SEC-300A column equilibrated with 10 mM TRIS (pH 6.5), 150 mM NaCl and 100 mM malic acid. The column was calibrated with the Gel Filtration Molecular Weight Marker kit from Sigma-Aldrich (Cytochrome C [12.4 kDa], Carbonic Anhydrase [29 kDa], Bovine Serum Albumin [66 kDa] and Sweet Potato Amylase [200 kDa]). Oxidized RitR was prepared by exposing 200 μM RitR to 1 mM H_2_O_2_ for 3 h. Reduced RitR was prepared by adding 1 mM DTT to the protein. The samples were injected (5 μl) onto the SEC-300A column and separated at a flow rate of 0.1 ml/min.

### Streptonigrin killing assays

Streptonigrin survival assays were essentially performed as described in ref. [25]. Iron-depleted medium was prepared by treating THY medium with 2% Chelex-100 (Sigma-Aldrich) overnight at 4°C followed by filter sterilization and addition of 2 mM MgSO_4_ and 100 μM CaCl_2_. Pneumococcal cells were grown in iron-depleted medium until the culture reached an OD_600_ of 0.2, whereupon cells where frozen in 1 ml aliquots at -80°C. Before challenge with streptonigrin, cells were quickly thawed, pelleted at 10,000 × g for 2 minutes and then resuspended in 1 ml of iron-depleted medium supplemented with 50 μM ammonium iron(II) sulfate (Sigma-Aldrich). The treated pneumococcal cells were then incubated at 37°C for 40 minutes prior to challenge with 4 μg/ml streptonigrin (Sigma-Aldirch). Samples were taken at various time-points and CFUs were determined by dilution and plating on TSA + 5% sheep's blood.

### Murine colonization model

Colonization experiments were done principally as described previously (ref. [41]), using approximately 1x10^**5**^ CFU of *S*. *pneumoniae* in 20 μl PBS, given intranasally. Colonization data were analysed by analysis of variance followed by Tukey’s multiple comparisons test. Statistical significance was considered to be a p-value of < 0.05 [40, 41].

### Ethics statement

Mouse colonization experiments were performed at the University of Leicester under appropriate project (P7B01C07A) and personal licenses (I66A9D84D) according to the United Kingdom Home Office guidelines under the Animals Scientific Procedures Act of 1986, and the University of Leicester ethics committee approval. The protocol was approved by both the U.K. Home Office and the University of Leicester ethics committee. The procedures were carried out under anesthetic with isoflurane. Animals were housed in individually ventilated cages in a controlled environment, and were frequently monitored after infection to minimize suffering.

### Cys128 pKa determination

The thiol group of Cys128, the only one in the protein (**S4 Fig**), was labeled with 5,5’-dithiobis-2-nitrobenzoic acid (DTNB) at a pH range from 5 to 8.5 according to the method of Palm *et al*. [62], as at a pH below 5 and above 8.5 the RitR protein was not stable enough to obtain reliable data. To produce a readout, the S-S bond in DTNB is cleaved by a protein thiol group to give 2-nitro-5-thiobenzoate (TNB), which absorbs light at 412 nm (ε_412nm_ = 14,150 M^-1^cm^-1^). RitR (5 μM) was reacted with 20 μM DTNB in 50 mM citric acid/HEPES/Bicine buffer (50 mM citric acid, 50 mM HEPES, 50 mM Bicine, 150 mM NaCl; the pH was adjusted by NaOH or HCl). The reactions were conducted at room temperature using an Evolution 300 UV-Vis spectrophotometer (Thermo Scientific). The absorption at 412 nm was recorded until the reaction was completed. The reaction rate at each pH value, k_(pH)_, was determined by fitting the spectra to the following equation:

$$A_{412}\left(t\right)=A_{412,0}+\Delta A_{412}\left(1-e^{-k\left(pH\right)t}\right)$$

Since the k_(pH)_ is proportional to the concentration of deprotonated thiol groups, the pK_a_ of Cys128 was obtained by fitting the following equation:

$$k_{\left(pH\right)}=\frac{C}{1+10^{pK_{a,thiol}-pH}}$$

where C is the maximum rate of the reaction.

### Non-reducing SDS-PAGE

Purified RitR was incubated in the presence of 10 mM DTT reducing agent and 20 mM oxidizing agent (where stated) for 20 minutes at room temperature. Oxidized/reduced proteins were then incubated for a further 20 minutes in the presence of iodoacetimide (IAA) at room temperature to trap any unreacted Cys128. These samples were then mixed with Laemelli's SDS loading buffer that lacked reducing agent, boiled for 2 minutes and resolved using a 12% SDS-PAGE gel. Proteins were revealed by staining with Coomassie Brilliant Blue for 30 minutes and then destained overnight.

### Electrophoretic mobility shift assay (EMSA)

HEX (Hexachlorofluorocein) labelled oligonucleotides corresponding to Piu promoter RitR binding sites BS1, BS2 and BS3 from three regions within the Piu promoter (**S1 Table**), as well as a control scrambled oligo, were used in EMSA experiments. 20 μl total volume of reaction mix was then used containing 20 mM HEPES, pH 7.2, 5 mM MgCl_2_, 1 mM CaCl_2_, 0.1 mM EDTA, 10% glycerol, 0.5 μM oligo, 800 ng polydeoxyinosinic-deoxycytidylic acid (Poly(dI-dC)), 10 mM DTT, and RitR at concentrations of between 0 to 6.6 μM. Where appropriate, RitR was oxidized by the addition of 20 mM H_2_O_2_ for 20 minutes at room temperature. Reaction mixtures were then resolved using a 4% non-denaturing 1x Tris-Acetate EDTA (TAE) gel. Gels were visualized at 560 nm for the HEX DNA probe using FLA3000 (FujiFilm) imager.

### Thermal shift assay

1.5 μM (final concentration) of purified RitR wild-type or C128S mutant protein was incubated for 20 minutes at room temperature in the presence of 50 mM HEPES pH 7.2, 150 mM NaCl, 1 mM DTT or 10 mM H_2_O_2_ and 1X SYPRO orange (Sigma) fluorescent dye in a total volume of 20 μl. Samples were then heated to 65°C with each reading taken in 0.1°C increments at 570 nm absorbance. Measurements were made using a qRT-PCR machine (RG-3000, Corbett Research).

### Immunoprecipitation of *in vivo* oxidized RitR

The presence of RitR and its cysteine disulphide bridge peptide were detected *in vivo* by culturing 500 ml of *S*. *pneumoniae* cells harboring the replicating pNE1 plasmid [74] in BHI in the presence of 200 μg/ml spectinomycin expressing either wild-type FLAG-RitR, or the FLAG-RitR C128S variant protein. The cells were grown either in the absence of oxygen in an anaerobic chamber, in 5% CO_2_ (statically), or with aeration in an incubating shaker (aerobically) until an OD_600_ of 0.4 was reached (mid-log phase). The cells were then pelleted and resuspended in lysis buffer (50 mM TRIS pH 8.0, 100 mM NaCl, 0.5% Triton X-100, 10 mM NaF, one PhosSTOP and one cComplete protease inhibitor cocktail pellet (Roche), 1 mM EDTA and to trap unreacted cysteines before cell disruption 10 mM iodoacetamide (IAA). Cells were then sonicated for 4 × 30 second cycles using a Branson 450 Watt cell disruptor. After sonication, the cell lysates were clarified by centrifugation for 30 minutes at 19,000 rpm before adding to 50 μl of M2 FLAG-conjugated magnetic beads (Sigma-Aldrich) pre-equilibrated in wash buffer. Samples were then incubated at room temperature for 2 hours, washed for 3 × 5 minutes in wash buffer (50 mM TRIS-HCl pH 8.0, 100 mM NaCl, 0.5% Triton X-100, 10 mM NaF, with PhosSTOP and cComplete protease inhibitor cocktail pellets (Roche)), and eluted by 2 × 20 minute incubations with 0.5 ml of elution buffer (50 mM TRIS pH 8.0, 100 mM NaCl and 200 μg/ml 3 × FLAG peptide from Sigma-Aldrich). After confirming by non-reducing SDS-PAGE coupled to western blot analysis the presence of RitR monomer and higher molecular weight forms, samples from the monomer, dimer, and intermediate molecular weight third band observed (see **Fig 2E**) were then excised and sent for analysis as separate samples to University of Wisconsin (UW-Madison Biotechnology Center) for mass spectrometry analysis.

### Identification of the Cys128 disulfide bridged peptide by mass spectrometry

#### In-gel digestion

In-gel digestion and mass spectrometric analysis was carried out at the UW-Madison Biotechnology Center by dicing excised bands into ~1 mm^3^ pieces and washing with >10 volumes of water. Coomassie Blue R-250 stained gel pieces were destained twice for 5 minutes in 200 μl 50% MeOH, 50% 100 mM NH_4_HCO_3_, dehydrated for 5 minutes in 50% CAN, 50% 25 mM NH_4_HCO_3,_ and then once more for 1 minute in 100% acetonitrile (ACN). Gel pieces were then fully dried by Speed-Vac for 2 minutes. Importantly, the proteins were not subjected to reduction in order to preserve their disulfide state. Free thiols were alkylated with 55 mM iodoacetamide in 25 mM NH_4_HCO_3_ in darkness at room temperature for 30 minutes, and then washed twice in H_2_O for 30 seconds and equilibrated in 25 mM NH_4_HCO_3_ for 1 minute. A second dehydration was then carried out in 50% ACN, 50% 25 mM NH_4_HCO_3_ for 5 minutes, followed by a 30 second incubation in 100% CAN. Gel pieces were then rehydrated with 20 μl of trypsin solution (10 ng/μl trypsin Gold (Promega) in 25 mM NH_4_HCO_3_ / 0.01% ProteaseMAX w/v (Promega)), then overlaid with an additional 30 μl of digestion solution (25 mM NH_4_HCO_3_ / 0.01% w/v ProteaseMAX) to facilitate complete rehydration with a small excess volume needed for peptide extraction. The digestion was conducted for 3 hours at 42°C. Peptides generated from digestion were then transferred to a new tube and acidified with 2.5% Trifluoroacetic Acid (TFA) and brought to a final TFA percentage of 0.3%. Degraded ProteaseMAX was removed via centrifugation at maximum speed for 10 minutes, and the peptides were then cleaned up by solid phase extraction using ZipTip C18 pipette tips (Millipore, Billerica, MA) according to the manufacturer’s instructions.

#### LC-MS/MS

Peptides were analyzed by nano LC-MS/MS using the Agilent 1100 Nanoflow system (Agilent) connected to a hybrid linear ion trap Orbitrap mass spectrometer (LTQ-Orbitrap XL, Thermo Fisher Scientific) equipped with an EASY-Spray electrospray source. Chromatography of peptides prior to mass spectral analysis was accomplished using a capillary column with integrated electrospray emitter (PepMap C18, 3 μM, 100 Å, 150 × 0.075 mm, Thermo Fisher Scientific) onto which extracted peptides were automatically loaded. The NanoHPLC system delivered solvents A: 0.1% (v/v) formic acid in water, and B: 99.9% (v/v) acetonitrile, 0.1% (v/v) formic acid at 0.5 μl/minute during peptide loading (30 minutes) and 0.3 μl/minute during elution. Peptides were gradient eluted over 35 minutes from 0% B to 40% B followed by a 5 minute ramp from 40% B to 100% B, and a return to 0% for re-equilibration. The mass spectrometer was operated in data-dependent mode with centroid survey MS scans acquired in the Orbitrap at 100,000 resolving power over the m/z range 300–2000, and up to 5 of the most intense peptide precursors per survey scan isolated, fragmented, and detected in the LTQ ion trap. Precursor redundancy was limited by dynamic exclusion and only precursors of known charge ≥2 were selected. Raw MS/MS data were converted to mgf file format using MSConvert (ProteoWizard: Open Source Software for Rapid Proteomics Tools Development [81]). The resulting mgf files were submitted for searching by Mascot (Matrix Science) version 2.2 against a *Streptococcus pneumoniae* R6 database containing 3645 sequences, including mutant RitR sequences. Peptide mass tolerances for searching were set at 15 ppm for precursor masses and 0.8 Da for fragment masses.

### Crystallization, structure determination, and model refinement

Crystals of selenomethionine (SeMet)-substituted RitR_C128S_ were grown by the hanging-drop, vapor diffusion method. Drops were comprised of equal parts protein solution (20 mg/ml RitR_C128S_ in 25 mM TRIS, pH 8.0) and crystallization solution (25% (w/v) polyethylene glycol (PEG) 3350, 0.1 M ammonium acetate). Partially hollow rods appeared after several days at 16°C. Crystals were prepared for flash-cooling by sequential soaks in solutions containing 25% PEG (w/v) 3350, 0.2 M ammonium acetate, and 5, 10, or 20% glycerol (v/v). Crystals of RitR_C128D_ were grown using a similar protocol, where drops contained 1 μl of protein solution (15.3 mg/mL RitR_C128D_ in 10 mM ADA pH 7.0) and 1 μl of crystallization solution (0.1 M HEPES pH 7.5, 22.5% Jeffamine ED-2001 pH 7.0). Long, rod-shaped crystals appeared after 3 days at 16°C. Crystals were prepared for flash-cooling by sequential soaks in solutions containing 0.1 M HEPES pH 7.5, 22.5% Jeffamine ED-2001 pH 7.0, and 5, 10, or 20% glycerol (v/v). Crystals of RitR_OX_ were also grown by the hanging-drop, vapor diffusion method with drops formed from 2 μl of protein solution (6 mg/ml RitR_OX_ in 25 mM TRIS, 150 mM NaCl, 100 mM malic acid, 1 mM H_2_O_2_, pH 6.5) and 1 μl of crystallization solution (10% (w/v) polyethylene glycol (PEG) 3350, 0.3 M magnesium sulfate). Prism-shaped crystals appeared after several days at 16°C. Crystals were prepared for flash-cooling by soaking them briefly in a solution containing 10% (w/v) PEG 3350, 0.3 M magnesium sulfate, and 20% glycerol (v/v). X-ray diffraction data for SeMet RitR_C128S_ were collected at beamline 21-ID-D of the Life Science Collaborative Access Team (LS-CAT) at the Advanced Photon Source (APS, Chicago, IL). The RitR_C128D_ data set was collected at LS-CAT beamline 21-ID-G. The RitR_OX_ data set was collected at LS-CAT beamline 21-ID-F. Data were processed with HKL2000 [82].

The structure of RitR_C128S_ was determined by the single-wavelength anomalous diffraction (SAD) method using 1.7 Å-resolution data collected from a crystal of SeMet-substituted RitR_C128S_ at 0.97889 Å, 61.0 eV below the tabulated K-edge wavelength for Se (0.97950 Å). The program autoSHARP [83] was used to solve the Se substructure, which contained 16 of the 18 Se atoms in the asymmetric unit, and calculate density-modified electron density maps. An initial model comprising almost complete asymmetric unit contents was built using PHENIX.Autosol [84]. Chain A of this refined model (with B-factors set to 20.0 Å^2^) was subsequently used as the search model for molecular replacement in PHASER [85] to phase the RitR_C128D_ and RitR_OX_ structures. All three models were subjected to iterative cycles of manual model building in COOT [86] and maximum likelihood-based refinement using the PHENIX package (phenix.refine [87]). Ordered solvent molecules were added automatically in phenix.refine and culled manually in COOT. Hydrogen atoms were added to the model using phenix.reduce [88] and were included in the later stages of refinement to improve the stereochemistry of the model. Positions of H atoms were refined using the riding model with a global B-factor. Regions of the model for translation-libration-screw (TLS) refinement were identified using phenix.find_tls_groups [89] and the TLS parameters were refined in phenix.refine. Once the refinement converged, these models were validated using the tools implemented in COOT and PHENIX [90, 91]. Sections of the backbone with missing or uninterpretable electron density were not included in the final model.

Data collection and model refinement statistics are listed in **Table 1**. Coordinates and structure factors for RitR_C128S,_ RitR_C128D_ and RitR_OX_ models have been deposited in the Protein Data Bank (www.rcsb.org) with accession codes 5U8K, 5VFA, and 5U8M, respectively.

### Generation of alignments and phylogenetic trees

Alignments for phylogenetic trees were done as previously described using Clustal W [92] to align and MacBoxShade for output details [93]. Phylogenetic trees were generated from the Clustal W alignment using Archaeopteryx [94] as previously described [30].

### Computational determination of linker cysteine residues

To the best of our knowledge RitR is the first example of a redox sensor that uses a Cys residue within the linker domain of the protein for its sensory functions. Because it is highly reactive, serving as both a reversible structural component through disulfide bridge formation, and also acting as a nucleophile in enzymatic reactions, cysteine is the third rarest amino acid found in proteins (the first two are tryptophan and methionine). To better predict if the RitR mechanism of signaling through linker region cysteine oxidation might be a more widespread mechanism in nature we used a computational approach.

As a first step we created a fasta file (PF00486.full.unaligned.fasta) containing all full-length, unaligned (gaps removed) protein sequences harboring a Pfam PF00486 domain (*i*.*e*. DNA-binding domain). The sequences were retrieved from the Pfam 26.0 database and also cross-referenced to Swiss-Prot to derive whole protein sequences. A third party tool was then implemented, called pfam_scan.pl, against generated PF00486.full.unaligned.fasta to annotate all Pfam domains and their locations within the full protein sequence. From the pfam_scan.pl output, regions between PF00072 (REC domain) and PF00486 (DNA-binding domain) extracted sequences were then classified as a 'linker’ region. This strategy was implemented because linker regions tend to have little homology compared to *bona fide* domain architectures. Sequences that did not match the N-PF00072-<linker>-PF00486-C architecture were excluded. When building the linker database protein accession numbers with the correct architecture and several additional statistics were gathered, including linker length, number of cysteines per linker, and the relative location of the cysteines within our computationally-defined linker region. Outputs were generated for one Cys per linker, and separate outputs for one or more cysteines. Taxonomic information was derived by performing a protein accession cross-reference from a dataset harboring at least one cysteine per linker to a Swiss-Prot protein database containing the taxonomic IDs. Using the Swiss-Prot protein database, a custom taxonomic hierarchy database was then generated, tracking the number of proteins of PF00486 that fell under any given taxonomic class. This database was then, in turn, used to generate an html chart with the desired statistics for further analyses.

Programs and scripts were written in Python, with supporting tools in Perl for extracting domains and their putative ranges in protein alignments.

### Nuclear magnetic resonance (NMR)

RitR wild-type and RitR_C128S_ protein samples were labeled with ^15^N, purified and 2-dimensional (2D) NMR performed as described by Maule *et al*. [30] at the Medical College of Wisconsin, Department of Biochemistry. 16 scans were acquired per Heteronuclear Single Quantum Coherence (HSQC) spectrum. The final concentration of the samples before data collection were both 360 μM. For the wild-type protein, DTT (1.4 mM and 10 mM final concentrations) was titrated directly into the Shigemi NMR sample tube before data was then again acquired.

### Accession numbers

Structures of RitR_C128S_, RitR_C128D_, and oxidized RitR (RitR_OX_) have been deposited in the Protein Data Bank (www.rcsb.org) under accession codes 5U8K, 5VFA, and 5U8M, respectively.

## Supporting information

S1 Fig Additional D39 data. (**A**) Western blot analysis representative of three independent experiments of PiuA iron transporter lipoprotein levels in the D39 *ritR* genetic background variants grown in 5% CO_2_. RitR and PiaA, another iron transporter lipoprotein, are used as controls. (**B**) qRT-PCR analysis of *piuA* and *ritR* mRNA from cells grown in 5% CO_2_, presented as relative quantities after normalization to *gyrB*. Error bars represent standard deviation of the mean of 4 independent experiments. Statistics were calculated using a one-way ANOVA followed by Tukey’s multiple comparison test. (**C**) Piu promoter activity in the genetic background of D39 *ritR* variants in 5% CO_2_. (**D**) Streptonigrin killing of the D39 *ritR* genetic variants. (**E**) D39 and R800 WT H_2_O_2_ concentration under anaerobic (-O_2_), 5% CO_2_ and aerobic (+O_2_) conditions. Samples of *S*. *pneumoniae* cells were cultured to early exponential phase (0.1–0.2) followed by measurement of H_2_O_2_ concentration in the culture medium with an Amplex Red detection kit (Invitrogen). H_2_O_2_ concentration was normalized to the optical density of the culture. Above the graph bars are the average raw concentrations of H_2_O_2_ for comparison. Unless otherwise noted, graphs are a result of three independent experiments and error bars represent +/- standard deviation. One asterisk indicates a p-value of ≤0.05, two of ≤0.01, three of ≤0.001, and four of ≤ 0.0001 as determined by one-way ANOVA followed by Tukey’s multiple comparison test in *C*. A two-tailed students t-test was used in *D* and *E*. ns, not significant.
(TIF)

S2 Fig Cysteine 128 requirement for *in vivo* dimerization. **(A)** Western blot showing the levels of PiuA and FLAG-RitR expressed in R800 strains used in the experiment in *B*. (**B**) Coomassie and anti-FLAG western blot of R800 wild-type (wt) and C128A mutant *in vivo* immunoprecipitation assay. MW, molecular weight ladder; (+) oxygen = cells were aerated, (+/-) oxygen = cells were grown statically in 5% CO_2_ and (-) oxygen = cells were grown anaerobically before RitR was immunoprecipitated. D1, predicted dimeric RitR; M, predicted monomeric RitR; D2, RitR-containing heterologous protein complex. **(C)** Mass spectrometry coverage of D1, D2 and M bands from *B*. All RitR amino acids/peptides identified by MS are colored red, and ones that were not identified are black. Cys128 is colored green and bolded. Note the absence of the GRDFIDQHCSLMKVPR peptide (boxed) only in the oxidized samples. **(D)** The GRDFIDQHCSLMKVPR peptide (colored red) with Cys128 (colored green spheres) mapped onto the cartoon representation of the RitR C128S structure and **(E)** RitR oxidized structure. The REC/ALR domain is shown in black and the linker and DBD in grey.
(TIF)

S3 Fig RitR Cys128 contribution to protein stability and pKa. (**A**) Overlay of the ribbon diagrams for the full-length RitR_C128S_ (ALR/REC domain is colored blue, the linker and DBD colored orange and Ser128 (Cys128) colored yellow) and the RitR ALR/REC domain structure alone (colored grey, PDB ID: 4LZL). (**B**) Overlay of the C128S RitR structure (in color) and the most similar available full-length structure, MtrA (in grey; PDB ID: 2GWR). The overlay emphasizes the unusual fully structured N-terminal linker helix in RitR (in orange), versus a typical unstructured helix in the MtrA response regulator. The Gly120 hinge is colored green and Ser128 (Cys128) yellow. For clarity the DBD of the RitR C128S structure was omitted from the overlay. (**C**) Close-up cartoon structural representation of helix 5, Cys128 and nearby electrostatic interactions in the C128S structure. Helix 5 is shown as a heat map according to the structure’s calculated B-factors, where dark blue is more stable than green/yellow. Notice that despite the lack of stability around Cys128, the structure can still maintain its helical integrity, likely through the extensive hydrogen bonding (depicted by black dashed lines). **(D)** Overlay of RitR C128S (in color) and C128D (colored grey) structures. The region colored magenta in the C128S structure, including Ser128 (*i*.*e*. Cys128) itself (colored yellow) is missing electron density in C128D, suggesting that the C>D substitution creates local instability in this region of the protein. **(E)** Graph showing the DNTB assay indicating that the pKa of Cys128 (pH 6.85) is more acidic than free cysteine (pH 8.5, shown by the arrow). **(F)** Thermal shift assay comparing wild-type (wt) versus C128S RitR samples. Note that a switch in protein stability (of 2.5°C) only occurs when Cys128 is present in the wild-type sample. K, thousands of fluorescent units.
(TIF)

S4 Fig Alignment of full amino acid sequences from CovR and RitR homologs. RitR-containing species are colored red and CovR-containing species black. The conserved phosphorylatable Asp residue found in canonical REC domains (*i*.*e*. CovR) are shaded in blue, and the conserved Cys128 in RitR homologs shaded in yellow. Identical residues are colored black and similar residues are colored grey.
(TIF)

S5 Fig Comparison of RitR, RitR_OX_ and PhoB DNA binding domains (DBDs). **(A)** A Clustal Omega alignment of RitR and PhoB DBDs. PhoB residues that contact DNA in the structure (PBD code 1GXP; ref. [47]) are colored in yellow, and corresponding RitR residues in cyan. Asterisks represent identical residues. **(B)** Cartoon representation of PhoB DBD structure (1GXP) and residues (in yellow) that contact its cognate DNA. Nitrogens are colored blue and oxygens red. Dotted lines indicate probable electrostatic interactions. **(C)** Cartoon overlay of the PhoB DBD structure from *B* (in grey) and a RitR structural model (in cyan) based on the alignment in *A* (using Swiss Model, ref. [96]). Notice the similarities between PhoB and RitR residues that contact DNA and their relative positions. **(D)** Cartoon representation of the RitR_C128S_ structure showing RitR DBD residues (cyan), which are predicted to bind DNA based on the PhoB 1GXP structure, but instead here form hydrogen bonds to REC domain (blue) residues in the absence of DNA. The DBD is colored grey and the linker domain orange. **(E)** Transparent surface representation of *D* showing how RitR residues predicted to bind DNA are sandwiched within the inactive structure. (**F**) Comparison of RitR DBD and PhoB DBD electrostatics. Negative charges are shown in red and positive charges in blue. Structures are shown as cartoons within a transparent surface representation. All figures were created using MacPyMol.
(TIF)

S6 Fig Comparison of RitR and RitR_OX_ DNA-binding domains (DBDs). **(A)** Overlay of a cartoon representation of the DBD of RitR_C128S_ (cyan) and RitR_OX_ (pink) structures. The other RitR_OX_ protomer (on right side of figure) is colored blue (REC/ALR domain), orange (linker) and grey (DBD). Labeled residues are alleged to make contacts with DNA based on comparisons to the PhoB structure 1GXP [47]. Nitrogens are colored blue and oxygens red. Red dotted lines represent predicted electrostatic interactions between DBD and REC residues. (**B**) Comparison of RitR_C128S_ DBD, RitR_OX_ DBD and PhoB DBD electrostatics. Negative charges are shown in red and positive charges in blue. Structures are shown as cartoons within a transparent surface representation. All figures were created using MacPyMol.
(TIF)

S7 Fig Regional stability comparison of RitR_C128S_ and RitR_OX_ structures. **(A)** Stability of the RitR_C128S_ and RitR_OX_ structures. B-factors of individual alpha carbons were divided by the calculated average B-factor value for the entire structure (y-axis), and graphed in accordance to residue position (x-axis). A value of 1 indicates the average value, and higher and lower numbers indicate less and more stability than the calculated structural average, respectively. The position of Cys128 is indicated by the dotted line. The grey line represents B-factor deviation in the RitR_C128S_ structure, and the orange line the RitR_OX_ structure. Key residues that vary greatly in B-factor values from the average in the RitR_C128S_ structure are labeled and indicated by the arrows **(B)** Cartoon representation (left panel) and B-factor putty representation (right panel) of the β6-β7-β8-α6 region of the RitR_C128S_ structure. Predicted electrostatic interactions are shown as dotted lines. **(C)** Cartoon representation (left panel) and B-factor putty representation (right panel) of the β6-β7-β8-α6 region of the RitR_OX_ structure. In the cartoon the two RitR protomers are colored orange and purple for clarity. Predicted electrostatic interactions are shown as dotted lines. Notice the additional hydrogen bonds provided between Ser129-Ser129’ and Met130-His127’, presumably accounting for enhanced stabilization observed in the domain-swapped structure.
(TIF)

S8 Fig Additional cysteine-linker domain informatics data. **(A)** Linker domain length (y-axis) versus the number of linker sequences with the length in question (x-axis). Data were derived from 3,623 total deposited sequences containing an N-terminal REC sensory domain, linker, and C-terminal DNA-binding domain. **(B)** Logo diagram showing residue frequency (proportional to the letter size; y-axis) versus position from the linker Cys (-4 to +4; x-axis). The cysteine (C) is colored orange, arginines (R) in purple and glycines (G) green. **(C)** Structural model of a helix containing the most frequent Cys-containing linker peptide ("ERLRCGLLR") showing that arginines are in positions to influence Cys pKa directly, and that the glycines are positioned adjacent to the cysteine for predicted increased helical flexibility. **(D)** Graph showing the number of Cys-containing linker domains per class of bacteria. Photosynthetic microbes are colored in green, and anaerobes in purple. An asterisk denotes a higher order phyla, *e*.*g*. Lentisphaerae, Spartobacteria and Opitutae are all under the Verrucomicrobia phyla.
(TIF)

S9 Fig 2-dimensional HSQC spectra of DTT titrated RitR. ^1^H-^15^N HSQC data were acquired with ^15^N-labeled RitR wild-type and RitR_C128S_ samples (360 μM, 16 scans). (**A**) The wild-type sample data was collected first, and then two additional datasets collected on the same (wt) sample after spiking with 1.4 mM DTT and then 10 mM DTT, respectively. All three spectrums are shown in a single overlay. Red spectrum, no DTT added; Green spectrum, 1.4 mM DTT added; Blue spectrum, 10 mM DTT added. Identical representative portions of all three spectrums are shown above the overlay for comparison. Notice the overall chemical shift peaks become less broad as DTT is added, indicating that the structure size is decreasing as the sample becomes reduced (to monomeric form). (**B**) A RitR_C128S_ HSQC spectrum is shown for comparison. Notice that the RitR_C128S_ spectrum more closely resembles the reduced (monomeric) wild-type sample with 10 mM DTT in *A*.
(TIF)

S10 Fig Effects of metal addition on RitR dimerization and DNA binding. (**A**) SEC of RitR (116 μM final concentration) wild-type samples before (blue lines) and then after (magenta lines) a pre-incubation with a 1 mM final concentration of the following metals: copper (Cu), iron (Fe), magnesium (Mg), zinc (Zn), calcium (Ca) and manganese (Mn). For comparison, blue lines represent SEC profiles for wild-type RitR (116 μM) with no addition. Notice only copper (CuCl_2_) is able to cause RitR to dimerize. Conditions of the SEC runs are identical to that of Fig 2. The lower (small) molecular weight peak eluting at 6.6 minutes corresponds to free copper ions. (**B**) EMSA of the HEX-labeled *piu* BS3 double stranded oligo and purified RitR (1 μM) with increasing concentrations of CuCl_2_ (from 0 to 100 μM concentration). No shift is observed, indicating that copper does not influence RitR in binding *piu* BS3 DNA. Unless otherwise noted here, the EMSA experiment was carried out as per Fig 2. mAU; milli Absorbance Units.
(TIF)

S1 Table Plasmids, strains and primers used in this study. (PDF)

## References

[1] Swanner ED, Mloszewska AM, Cirpka OA, Schoenberg R, Konhauser KO, Kappler A. Modulation of oxygen production in Archaean oceans by episodes of Fe(II) toxicity. Nature Geoscience. 2015;8:126–30.

[2] Imlay JA, Chin SM, Linn S. Toxic DNA damage by hydrogen peroxide through the Fenton reaction *in vivo* and *in vitro*. Science. 1988;240(4852):640–2. Epub 1988/04/29. . 2834821 10.1126/science.2834821

[3] Posey JE, Gherardini FC. Lack of a role for iron in the Lyme disease pathogen. Science. 2000;288(5471):1651–3. Epub 2000/06/02. . 10834845 10.1126/science.288.5471.1651

[4] Ezraty B, Gennaris A, Barras F, Collet JF. Oxidative stress, protein damage and repair in bacteria. Nat Rev Microbiol. 2017;15(7):385–96. doi: 10.1038/nrmicro.2017.26 . 28420885 10.1038/nrmicro.2017.26

[5] Paulsen CE, Carroll KS. Orchestrating redox signaling networks through regulatory cysteine switches. ACS Chem Biol. 2010;5(1):47–62. doi: 10.1021/cb900258z ; PubMed Central PMCID: PMCPMC4537063. 19957967 10.1021/cb900258z PMC4537063

[6] Antelmann H, Helmann JD. Thiol-based redox switches and gene regulation. Antioxidants & redox signaling. 2011;14(6):1049–63. Epub 2010/07/16. doi: 10.1089/ars.2010.3400 ; PubMed Central PMCID: PMC3113447. 20626317 10.1089/ars.2010.3400 PMC3113447

[7] Zheng M, Aslund F, Storz G. Activation of the OxyR transcription factor by reversible disulfide bond formation. Science. 1998;279(5357):1718–21. Epub 1998/03/28. . 9497290 10.1126/science.279.5357.1718

[8] Hong M, Fuangthong M, Helmann JD, Brennan RG. Structure of an OhrR-*ohrA* operator complex reveals the DNA binding mechanism of the MarR family. Molecular cell. 2005;20(1):131–41. Epub 2005/10/08. doi: 10.1016/j.molcel.2005.09.013 . 16209951 10.1016/j.molcel.2005.09.013

[9] Newberry KJ, Fuangthong M, Panmanee W, Mongkolsuk S, Brennan RG. Structural mechanism of organic hydroperoxide induction of the transcription regulator OhrR. Molecular cell. 2007;28(4):652–64. Epub 2007/11/29. doi: 10.1016/j.molcel.2007.09.016 . 18042459 10.1016/j.molcel.2007.09.016

[10] Lee JW, Helmann JD. The PerR transcription factor senses H2O2 by metal-catalysed histidine oxidation. Nature. 2006;440(7082):363–7. Epub 2006/03/17. doi: 10.1038/nature04537 . 16541078 10.1038/nature04537

[11] Traore DA, El Ghazouani A, Jacquamet L, Borel F, Ferrer JL, Lascoux D, et al Structural and functional characterization of 2-oxo-histidine in oxidized PerR protein. Nature chemical biology. 2009;5(1):53–9. Epub 2008/12/17. doi: 10.1038/nchembio.133 . 19079268 10.1038/nchembio.133

[12] Faulkner MJ, Helmann JD. Peroxide stress elicits adaptive changes in bacterial metal ion homeostasis. Antioxidants & redox signaling. 2011;15(1):175–89. Epub 2010/10/28. doi: 10.1089/ars.2010.3682 ; PubMed Central PMCID: PMC3110094. 20977351 10.1089/ars.2010.3682 PMC3110094

[13] Yesilkaya H, Andisi VF, Andrew PW, Bijlsma JJ. *Streptococcus pneumoniae* and reactive oxygen species: an unusual approach to living with radicals. Trends in microbiology. 2013;21(4):187–95. Epub 2013/02/19. doi: 10.1016/j.tim.2013.01.004 . 23415028 10.1016/j.tim.2013.01.004

[14] Pericone CD, Park S, Imlay JA, Weiser JN. Factors contributing to hydrogen peroxide resistance in *Streptococcus pneumoniae* include pyruvate oxidase (SpxB) and avoidance of the toxic effects of the fenton reaction. Journal of bacteriology. 2003;185(23):6815–25. Epub 2003/11/18. doi: 10.1128/JB.185.23.6815-6825.2003 ; PubMed Central PMCID: PMC262707. 14617646 10.1128/JB.185.23.6815-6825.2003 PMC262707

[15] Echlin H, Frank MW, Iverson A, Chang TC, Johnson MD, Rock CO, et al Pyruvate Oxidase as a Critical Link between Metabolism and Capsule Biosynthesis in *Streptococcus pneumoniae*. PLoS pathogens. 2016;12(10):e1005951 doi: 10.1371/journal.ppat.1005951 ; PubMed Central PMCID: PMCPMC5070856. 27760231 10.1371/journal.ppat.1005951 PMC5070856

[16] Weiser JN, Bae D, Epino H, Gordon SB, Kapoor M, Zenewicz LA, et al Changes in availability of oxygen accentuate differences in capsular polysaccharide expression by phenotypic variants and clinical isolates of *Streptococcus pneumoniae*. Infect Immun. 2001;69(9):5430–9. doi: 10.1128/IAI.69.9.5430-5439.2001 ; PubMed Central PMCID: PMCPMC98654. 11500414 10.1128/IAI.69.9.5430-5439.2001 PMC98654

[17] Lisher JP, Tsui HT, Ramos-Montanez S, Hentchel KL, Martin JE, Trinidad JC, et al Biological and Chemical Adaptation to Endogenous Hydrogen Peroxide Production in *Streptococcus pneumoniae* D39. mSphere. 2017;2(1). doi: 10.1128/mSphere.00291-16 ; PubMed Central PMCID: PMCPMC5214746. 28070562 10.1128/mSphere.00291-16 PMC5214746

[18] Brissac T, Shenoy AT, Patterson LA, Orihuela CJ. Cell invasion and pyruvate oxidase derived H2O2 are critical for *Streptococcus pneumoniae* mediated cardiomyocyte killing. Infect Immun. 2017 doi: 10.1128/IAI.00569-17 ; PubMed Central PMCID: PMCPMC5736805. 29061707 10.1128/IAI.00569-17 PMC5736805

[19] Orihuela CJ, Gao G, Francis KP, Yu J, Tuomanen EI. Tissue-specific contributions of pneumococcal virulence factors to pathogenesis. J Infect Dis. 2004;190(9):1661–9. doi: 10.1086/424596 . 15478073 10.1086/424596

[20] Andisi VF, Hinojosa CA, de Jong A, Kuipers OP, Orihuela CJ, Bijlsma JJ. Pneumococcal gene complex involved in resistance to extracellular oxidative stress. Infect Immun. 2012;80(3):1037–49. doi: 10.1128/IAI.05563-11 ; PubMed Central PMCID: PMCPMC3294666. 22215735 10.1128/IAI.05563-11 PMC3294666

[21] Saleh M, Bartual SG, Abdullah MR, Jensch I, Asmat TM, Petruschka L, et al Molecular architecture of *Streptococcus pneumoniae* surface thioredoxin-fold lipoproteins crucial for extracellular oxidative stress resistance and maintenance of virulence. EMBO Mol Med. 2013;5(12):1852–70. doi: 10.1002/emmm.201202435 ; PubMed Central PMCID: PMCPMC3914529. 24136784 10.1002/emmm.201202435 PMC3914529

[22] Bortoni ME, Terra VS, Hinds J, Andrew PW, Yesilkaya H. The pneumococcal response to oxidative stress includes a role for Rgg. Microbiology. 2009;155(Pt 12):4123–34. doi: 10.1099/mic.0.028282-0 ; PubMed Central PMCID: PMCPMC2885668. 19762446 10.1099/mic.0.028282-0 PMC2885668

[23] Kidd SP, Potter AJ, Apicella MA, Jennings MP, McEwan AG. NmlR of *Neisseria gonorrhoeae*: a novel redox responsive transcription factor from the MerR family. Molecular microbiology. 2005;57(6):1676–89. doi: 10.1111/j.1365-2958.2005.04773.x . 16135233 10.1111/j.1365-2958.2005.04773.x

[24] Potter AJ, Kidd SP, McEwan AG, Paton JC. The MerR/NmlR family transcription factor of *Streptococcus pneumoniae* responds to carbonyl stress and modulates hydrogen peroxide production. Journal of bacteriology. 2010;192(15):4063–6. doi: 10.1128/JB.00383-10 ; PubMed Central PMCID: PMCPMC2916378. 20525825 10.1128/JB.00383-10 PMC2916378

[25] Ulijasz AT, Andes DR, Glasner JD, Weisblum B. Regulation of iron transport in *Streptococcus pneumoniae* by RitR, an orphan response regulator. Journal of bacteriology. 2004;186(23):8123–36. Epub 2004/11/18. doi: 10.1128/JB.186.23.8123-8136.2004 ; PubMed Central PMCID: PMC529065. 15547286 10.1128/JB.186.23.8123-8136.2004 PMC529065

[26] Ulijasz AT, Falk SP, Weisblum B. Phosphorylation of the RitR DNA-binding domain by a Ser-Thr phosphokinase: implications for global gene regulation in the streptococci. Molecular microbiology. 2009;71(2):382–90. Epub 2008/12/02. doi: 10.1111/j.1365-2958.2008.06532.x . 19040630 10.1111/j.1365-2958.2008.06532.x

[27] Ong CL, Potter AJ, Trappetti C, Walker MJ, Jennings MP, Paton JC, et al Interplay between manganese and iron in pneumococcal pathogenesis: role of the orphan response regulator RitR. Infect Immun. 2013;81(2):421–9. doi: 10.1128/IAI.00805-12 ; PubMed Central PMCID: PMCPMC3553810. 23184523 10.1128/IAI.00805-12 PMC3553810

[28] McCluskey J, Hinds J, Husain S, Witney A, Mitchell TJ. A two-component system that controls the expression of pneumococcal surface antigen A (PsaA) and regulates virulence and resistance to oxidative stress in *Streptococcus pneumoniae*. Molecular microbiology. 2004;51(6):1661–75. . 15009893 10.1111/j.1365-2958.2003.03917.x

[29] Sebert ME, Palmer LM, Rosenberg M, Weiser JN. Microarray-based identification of *htrA*, a *Streptococcus pneumoniae* gene that is regulated by the CiaRH two-component system and contributes to nasopharyngeal colonization. Infect Immun. 2002;70(8):4059–67. doi: 10.1128/IAI.70.8.4059-4067.2002 ; PubMed Central PMCID: PMCPMC128155. 12117912 10.1128/IAI.70.8.4059-4067.2002 PMC128155

[30] Maule AF, Wright DP, Weiner JJ, Han L, Peterson FC, Volkman BF, et al The aspartate-less receiver (ALR) domains: distribution, structure and function. PLoS pathogens. 2015;11(4):e1004795 Epub 2015/04/16. doi: 10.1371/journal.ppat.1004795 ; PubMed Central PMCID: PMC4395418. 25875291 10.1371/journal.ppat.1004795 PMC4395418

[31] Bourret RB. Receiver domain structure and function in response regulator proteins. Current Opinion in Microbiology. 2010;13(2):142–9. doi: 10.1016/j.mib.2010.01.015 20211578 10.1016/j.mib.2010.01.015 PMC2847656

[32] Hoch JA, Silhavy TJ, editors. Two-component signal transduction. Washington, D.C.: American Society for Microbiology; 1995.

[33] Zschiedrich CP, Keidel V, Szurmant H. Molecular Mechanisms of Two-Component Signal Transduction. J Mol Biol. 2016;428(19):3752–75. doi: 10.1016/j.jmb.2016.08.003 ; PubMed Central PMCID: PMCPMC5023499. 27519796 10.1016/j.jmb.2016.08.003 PMC5023499

[34] Kadioglu A, Weiser JN, Paton JC, Andrew PW. The role of *Streptococcus pneumoniae* virulence factors in host respiratory colonization and disease. Nat Rev Microbiol. 2008;6(4):288–301. doi: 10.1038/nrmicro1871 . 18340341 10.1038/nrmicro1871

[35] Bogaert D, De Groot R, Hermans PW. *Streptococcus pneumoniae* colonisation: the key to pneumococcal disease. Lancet Infect Dis. 2004;4(3):144–54. doi: 10.1016/S1473-3099(04)00938-7 . 14998500 10.1016/S1473-3099(04)00938-7

[36] Jo I, Chung IY, Bae HW, Kim JS, Song S, Cho YH, et al Structural details of the OxyR peroxide-sensing mechanism. Proceedings of the National Academy of Sciences of the United States of America. 2015;112(20):6443–8. doi: 10.1073/pnas.1424495112 ; PubMed Central PMCID: PMCPMC4443364. 25931525 10.1073/pnas.1424495112 PMC4443364

[37] Husain M, Jones-Carson J, Song M, McCollister BD, Bourret TJ, Vazquez-Torres A. Redox sensor SsrB Cys203 enhances *Salmonella* fitness against nitric oxide generated in the host immune response to oral infection. Proceedings of the National Academy of Sciences of the United States of America. 2010;107(32):14396–401. doi: 10.1073/pnas.1005299107 ; PubMed Central PMCID: PMCPMC2922535. 20660761 10.1073/pnas.1005299107 PMC2922535

[38] Brown JS, Gilliland SM, Holden DW. A *Streptococcus pneumoniae* pathogenicity island encoding an ABC transporter involved in iron uptake and virulence. Molecular microbiology. 2001;40(3):572–85. . 11359564 10.1046/j.1365-2958.2001.02414.x

[39] van Opijnen T, Camilli A. A fine scale phenotype-genotype virulence map of a bacterial pathogen. Genome Res. 2012;22(12):2541–51. doi: 10.1101/gr.137430.112 ; PubMed Central PMCID: PMCPMC3514683. 22826510 10.1101/gr.137430.112 PMC3514683

[40] Al-Bayati FA, Kahya HF, Damianou A, Shafeeq S, Kuipers OP, Andrew PW, et al Pneumococcal galactose catabolism is controlled by multiple regulators acting on pyruvate formate lyase. Sci Rep. 2017;7:43587 doi: 10.1038/srep43587 ; PubMed Central PMCID: PMCPMC5327383. 28240278 10.1038/srep43587 PMC5327383

[41] Kahya HF, Andrew PW, Yesilkaya H. Deacetylation of sialic acid by esterases potentiates pneumococcal neuraminidase activity for mucin utilization, colonization and virulence. PLoS pathogens. 2017;13(3):e1006263 doi: 10.1371/journal.ppat.1006263 ; PubMed Central PMCID: PMCPMC5352144. 28257499 10.1371/journal.ppat.1006263 PMC5352144

[42] Toro-Roman A, Mack TR, Stock AM. Structural analysis and solution studies of the activated regulatory domain of the response regulator ArcA: A symmetric dimer mediated by the α4-β5-α5 face. Journal of molecular biology. 2005;349(1):11–26. doi: 10.1016/j.jmb.2005.03.059 15876365 10.1016/j.jmb.2005.03.059 PMC3690759

[43] Ramos-Montanez S, Tsui HC, Wayne KJ, Morris JL, Peters LE, Zhang F, et al Polymorphism and regulation of the *spxB* (pyruvate oxidase) virulence factor gene by a CBS-HotDog domain protein (SpxR) in serotype 2 *Streptococcus pneumoniae*. Molecular microbiology. 2008;67(4):729–46. doi: 10.1111/j.1365-2958.2007.06082.x . 18179423 10.1111/j.1365-2958.2007.06082.x

[44] Robinson VL, Wu T, Stock AM. Structural analysis of the domain interface in DrrB, a response regulator of the OmpR/PhoB subfamily. Journal of bacteriology. 2003;185(14):4186–94. doi: 10.1128/JB.185.14.4186-4194.2003 ; PubMed Central PMCID: PMCPMC164896. 12837793 10.1128/JB.185.14.4186-4194.2003 PMC164896

[45] Friedland N, Mack TR, Yu M, Hung LW, Terwilliger TC, Waldo GS, et al Domain orientation in the inactive response regulator *Mycobacterium tuberculosis* MtrA provides a barrier to activation. Biochemistry. 2007;46(23):6733–43. Epub 2007/05/22. doi: 10.1021/bi602546q ; PubMed Central PMCID: PMC2528954. 17511470 10.1021/bi602546q PMC2528954

[46] Gao R, Stock AM. Biological insights from structures of two-component proteins. Annu Rev Microbiol. 2009;63:133–54. Epub 2009/07/07. doi: 10.1146/annurev.micro.091208.073214 . 19575571 10.1146/annurev.micro.091208.073214 PMC3645274

[47] Blanco AG, Sola M, Gomis-Ruth FX, Coll M. Tandem DNA recognition by PhoB, a two-component signal transduction transcriptional activator. Structure. 2002;10(5):701–13. . 12015152 10.1016/s0969-2126(02)00761-x

[48] Makino K, Amemura M, Kawamoto T, Kimura S, Shinagawa H, Nakata A, et al DNA binding of PhoB and its interaction with RNA polymerase. J Mol Biol. 1996;259(1):15–26. Epub 1996/05/31. doi: S0022-2836(96)90298-3 [pii] doi: 10.1006/jmbi.1996.0298 . 8648643 10.1006/jmbi.1996.0298

[49] Blanco AG, Canals A, Bernues J, Sola M, Coll M. The structure of a transcription activation subcomplex reveals how σ70 is recruited to PhoB promoters. EMBO J. 2011;30(18):3776–85. Epub 2011/08/11. doi: emboj2011271 [pii] doi: 10.1038/emboj.2011.271 ; PubMed Central PMCID: PMC3173795. 21829166 10.1038/emboj.2011.271 PMC3173795

[50] Wall SB, Oh JY, Diers AR, Landar A. Oxidative modification of proteins: an emerging mechanism of cell signaling. Front Physiol. 2012;3:369 doi: 10.3389/fphys.2012.00369 ; PubMed Central PMCID: PMCPMC3442266. 23049513 10.3389/fphys.2012.00369 PMC3442266

[51] Kortemme T, Creighton TE. Ionisation of cysteine residues at the termini of model alpha-helical peptides. Relevance to unusual thiol pKa values in proteins of the thioredoxin family. J Mol Biol. 1995;253(5):799–812. doi: 10.1006/jmbi.1995.0592 . 7473753 10.1006/jmbi.1995.0592

[52] Edayathumangalam R, Wu R, Garcia R, Wang Y, Wang W, Kreinbring CA, et al Crystal structure of *Bacillus subtilis* GabR, an autorepressor and transcriptional activator of *gabT*. Proceedings of the National Academy of Sciences of the United States of America. 2013;110(44):17820–5. doi: 10.1073/pnas.1315887110 ; PubMed Central PMCID: PMCPMC3816445. 24127574 10.1073/pnas.1315887110 PMC3816445

[53] Ostrow AZ, Kalhor R, Gan Y, Villwock SK, Linke C, Barberis M, et al Conserved forkhead dimerization motif controls DNA replication timing and spatial organization of chromosomes in *S*. *cerevisiae*. Proceedings of the National Academy of Sciences of the United States of America. 2017;114(12):E2411–E9. doi: 10.1073/pnas.1612422114 ; PubMed Central PMCID: PMCPMC5373409. 28265091 10.1073/pnas.1612422114 PMC5373409

[54] Park CK, Joshi HK, Agrawal A, Ghare MI, Little EJ, Dunten PW, et al Domain swapping in allosteric modulation of DNA specificity. PLoS Biol. 2010;8(12):e1000554 doi: 10.1371/journal.pbio.1000554 ; PubMed Central PMCID: PMCPMC2998434. 21151881 10.1371/journal.pbio.1000554 PMC2998434

[55] Zhu L, Kreth J. The role of hydrogen peroxide in environmental adaptation of oral microbial communities. Oxid Med Cell Longev. 2012;2012:717843 doi: 10.1155/2012/717843 ; PubMed Central PMCID: PMCPMC3405655. 22848782 10.1155/2012/717843 PMC3405655

[56] Churchward G. The two faces of Janus: virulence gene regulation by CovR/S in group A streptococci. Molecular microbiology. 2007;64(1):34–41. doi: 10.1111/j.1365-2958.2007.05649.x . 17376070 10.1111/j.1365-2958.2007.05649.x

[57] Federle MJ, McIver KS, Scott JR. A response regulator that represses transcription of several virulence operons in the group A streptococcus. Journal of bacteriology. 1999;181(12):3649–57. ; PubMed Central PMCID: PMCPMC93840. 10368137 10.1128/jb.181.12.3649-3657.1999 PMC93840

[58] Poole LB. The basics of thiols and cysteines in redox biology and chemistry. Free Radic Biol Med. 2015;80:148–57. doi: 10.1016/j.freeradbiomed.2014.11.013 ; PubMed Central PMCID: PMCPMC4355186. 25433365 10.1016/j.freeradbiomed.2014.11.013 PMC4355186

[59] Marino SM, Gladyshev VN. Cysteine function governs its conservation and degeneration and restricts its utilization on protein surfaces. J Mol Biol. 2010;404(5):902–16. doi: 10.1016/j.jmb.2010.09.027 ; PubMed Central PMCID: PMCPMC3061813. 20950627 10.1016/j.jmb.2010.09.027 PMC3061813

[60] Walthers D, Tran VK, Kenney LJ. Interdomain linkers of homologous response regulators determine their mechanism of action. Journal of bacteriology. 2003;185(1):317–24. doi: 10.1128/JB.185.1.317-324.2003 ; PubMed Central PMCID: PMCPMC141822. 12486069 10.1128/JB.185.1.317-324.2003 PMC141822

[61] Karala AR, Lappi AK, Ruddock LW. Modulation of an active-site cysteine pKa allows PDI to act as a catalyst of both disulfide bond formation and isomerization. J Mol Biol. 2010;396(4):883–92. doi: 10.1016/j.jmb.2009.12.014 . 20026073 10.1016/j.jmb.2009.12.014

[62] Palm GJ, Khanh Chi B, Waack P, Gronau K, Becher D, Albrecht D, et al Structural insights into the redox-switch mechanism of the MarR/DUF24-type regulator HypR. Nucleic Acids Res. 2012;40(9):4178–92. doi: 10.1093/nar/gkr1316 ; PubMed Central PMCID: PMCPMC3351151. 22238377 10.1093/nar/gkr1316 PMC3351151

[63] Nelson AL, Barasch JM, Bunte RM, Weiser JN. Bacterial colonization of nasal mucosa induces expression of siderocalin, an iron-sequestering component of innate immunity. Cell Microbiol. 2005;7(10):1404–17. doi: 10.1111/j.1462-5822.2005.00566.x . 16153241 10.1111/j.1462-5822.2005.00566.x

[64] Panmanee W, Vattanaviboon P, Poole LB, Mongkolsuk S. Novel organic hydroperoxide-sensing and responding mechanisms for OhrR, a major bacterial sensor and regulator of organic hydroperoxide stress. Journal of bacteriology. 2006;188(4):1389–95. doi: 10.1128/JB.188.4.1389-1395.2006 ; PubMed Central PMCID: PMCPMC1367246. 16452421 10.1128/JB.188.4.1389-1395.2006 PMC1367246

[65] Soonsanga S, Lee JW, Helmann JD. Conversion of *Bacillus subtilis* OhrR from a 1-Cys to a 2-Cys peroxide sensor. Journal of bacteriology. 2008;190(17):5738–45. doi: 10.1128/JB.00576-08 ; PubMed Central PMCID: PMCPMC2519526. 18586944 10.1128/JB.00576-08 PMC2519526

[66] Gupta N, Ragsdale SW. Dual roles of an essential cysteine residue in activity of a redox-regulated bacterial transcriptional activator. J Biol Chem. 2008;283(42):28721–8. doi: 10.1074/jbc.M800630200 ; PubMed Central PMCID: PMCPMC2568902. 18687692 10.1074/jbc.M800630200 PMC2568902

[67] Choi H, Kim S, Mukhopadhyay P, Cho S, Woo J, Storz G, et al Structural basis of the redox switch in the OxyR transcription factor. Cell. 2001;105(1):103–13. . 11301006 10.1016/s0092-8674(01)00300-2

[68] Lo Conte M, Carroll KS. The redox biochemistry of protein sulfenylation and sulfinylation. J Biol Chem. 2013;288(37):26480–8. doi: 10.1074/jbc.R113.467738 ; PubMed Central PMCID: PMCPMC3772195. 23861405 10.1074/jbc.R113.467738 PMC3772195

[69] Szurmant H, White RA, Hoch JA. Sensor complexes regulating two-component signal transduction. Curr Opin Struct Biol. 2007;17(6):706–15. doi: 10.1016/j.sbi.2007.08.019 ; PubMed Central PMCID: PMCPMC2175030. 17913492 10.1016/j.sbi.2007.08.019 PMC2175030

[70] Pan X, Ge J, Li M, Wu B, Wang C, Wang J, et al The orphan response regulator CovR: a globally negative modulator of virulence in *Streptococcus suis* serotype 2. Journal of bacteriology. 2009;191(8):2601–12. doi: 10.1128/JB.01309-08 ; PubMed Central PMCID: PMCPMC2668425. 19181815 10.1128/JB.01309-08 PMC2668425

[71] Weinert LA, Chaudhuri RR, Wang J, Peters SE, Corander J, Jombart T, et al Genomic signatures of human and animal disease in the zoonotic pathogen *Streptococcus suis*. Nat Commun. 2015;6:6740 doi: 10.1038/ncomms7740 ; PubMed Central PMCID: PMCPMC4389249. 25824154 10.1038/ncomms7740 PMC4389249

[72] Manning SD, Springman AC, Million AD, Milton NR, McNamara SE, Somsel PA, et al Association of Group B Streptococcus colonization and bovine exposure: a prospective cross-sectional cohort study. PLoS One. 2010;5(1):e8795 doi: 10.1371/journal.pone.0008795 ; PubMed Central PMCID: PMCPMC2808344. 20098699 10.1371/journal.pone.0008795 PMC2808344

[73] Mongkolsuk S, Helmann JD. Regulation of inducible peroxide stress responses. Molecular microbiology. 2002;45(1):9–15. . 12100544 10.1046/j.1365-2958.2002.03015.x

[74] Bartilson M, Marra A, Christine J, Asundi JS, Schneider WP, Hromockyj AE. Differential fluorescence induction reveals *Streptococcus pneumoniae* loci regulated by competence stimulatory peptide. Molecular microbiology. 2001;39(1):126–35. . 11123694 10.1046/j.1365-2958.2001.02218.x

[75] Lefevre JC, Claverys JP, Sicard AM. Donor deoxyribonucleic acid length and marker effect in pneumococcal transformation. Journal of bacteriology. 1979;138(1):80–6. Epub 1979/04/01. ; PubMed Central PMCID: PMC218240. 35523 10.1128/jb.138.1.80-86.1979 PMC218240

[76] Sung CK, Li H, Claverys JP, Morrison DA. An *rpsL* cassette, janus, for gene replacement through negative selection in *Streptococcus pneumoniae*. Appl Environ Microbiol. 2001;67(11):5190–6. doi: 10.1128/AEM.67.11.5190-5196.2001 ; PubMed Central PMCID: PMCPMC93289. 11679344 10.1128/AEM.67.11.5190-5196.2001 PMC93289

[77] Lanie JA, Ng WL, Kazmierczak KM, Andrzejewski TM, Davidsen TM, Wayne KJ, et al Genome sequence of Avery's virulent serotype 2 strain D39 of *Streptococcus pneumoniae* and comparison with that of unencapsulated laboratory strain R6. Journal of bacteriology. 2007;189(1):38–51. doi: 10.1128/JB.01148-06 ; PubMed Central PMCID: PMCPMC1797212. 17041037 10.1128/JB.01148-06 PMC1797212

[78] Halfmann A, Hakenbeck R, Bruckner R. A new integrative reporter plasmid for *Streptococcus pneumoniae*. FEMS Microbiol Lett. 2007;268(2):217–24. doi: 10.1111/j.1574-6968.2006.00584.x . 17328748 10.1111/j.1574-6968.2006.00584.x

[79] Ulijasz AT, Grenader A, Weisblum B. A vancomycin-inducible lacZ reporter system in *Bacillus subtilis*: induction by antibiotics that inhibit cell wall synthesis and by lysozyme. Journal of bacteriology. 1996;178(21):6305–9. Epub 1996/11/01. ; PubMed Central PMCID: PMCPmc178505. 8892834 10.1128/jb.178.21.6305-6309.1996 PMC178505

[80] Jomaa M, Yuste J, Paton JC, Jones C, Dougan G, Brown JS. Antibodies to the iron uptake ABC transporter lipoproteins PiaA and PiuA promote opsonophagocytosis of *Streptococcus pneumoniae*. Infect Immun. 2005;73(10):6852–9. doi: 10.1128/IAI.73.10.6852-6859.2005 ; PubMed Central PMCID: PMCPMC1230898. 16177364 10.1128/IAI.73.10.6852-6859.2005 PMC1230898

[81] Kessner D, Chambers M, Burke R, Agus D, Mallick P. ProteoWizard: open source software for rapid proteomics tools development. Bioinformatics. 2008;24(21):2534–6. doi: 10.1093/bioinformatics/btn323 ; PubMed Central PMCID: PMCPMC2732273. 18606607 10.1093/bioinformatics/btn323 PMC2732273

[82] Otwinowski Z Fau—Minor W, Minor W. Processing of X-ray diffraction data collected in oscillation mode. (1557–7988 (Electronic)). 10.1016/S0076-6879(97)76066-X 27754618

[83] Vonrhein C, Blanc E, Roversi P, Bricogne G. Automated structure solution with autoSHARP. Methods in molecular biology. 2007;364:215–30. Epub 2006/12/19. doi: 10.1385/1-59745-266-1:215 . 17172768 10.1385/1-59745-266-1:215

[84] Terwilliger TC, Adams PD, Read RJ, McCoy AJ, Moriarty NW, Grosse-Kunstleve RW, et al Decision-making in structure solution using Bayesian estimates of map quality: the PHENIX AutoSol wizard. Acta Crystallographica Section D. 2009;65(6):582–601. doi: 10.1107/S0907444909012098 19465773 10.1107/S0907444909012098 PMC2685735

[85] McCoy AJ, Grosse-Kunstleve RW, Adams PD, Winn MD, Storoni LC, Read RJ. Phaser crystallographic software. Journal of applied crystallography. 2007;40(Pt 4):658–74. Epub 2007/08/01. doi: 10.1107/S0021889807021206 ; PubMed Central PMCID: PMC2483472. 19461840 10.1107/S0021889807021206 PMC2483472

[86] Emsley P, Lohkamp B, Scott WG, Cowtan K. Features and development of Coot. Acta Crystallogr Sect D Biol Crystallogr. 2010;66(Pt 4):486–501. Epub 2010/04/13. doi: 10.1107/S0907444910007493 ; PubMed Central PMCID: PMC2852313. 20383002 10.1107/S0907444910007493 PMC2852313

[87] Afonine PV, Mustyakimov M, Grosse-Kunstleve RW, Moriarty NW, Langan P, Adams PD. Joint X-ray and neutron refinement with phenix.refine. Acta Crystallogr Sect D Biol Crystallogr. 2010;66(Pt 11):1153–63. Epub 2010/11/03. doi: 10.1107/S0907444910026582 ; PubMed Central PMCID: PMC2967420. 21041930 10.1107/S0907444910026582 PMC2967420

[88] Word JM, Lovell SC, Richardson JS, Richardson DC. Asparagine and glutamine: using hydrogen atom contacts in the choice of side-chain amide orientation. Journal of molecular biology. 1999;285(4):1735–47. Epub 1999/01/26. doi: 10.1006/jmbi.1998.2401 . 9917408 10.1006/jmbi.1998.2401

[89] Afonine PV, Grosse-Kunstleve RW, Echols N, Headd JJ, Moriarty NW, Mustyakimov M, et al Towards automated crystallographic structure refinement with phenix.refine. Acta Crystallogr D Biol Crystallogr. 2012;68(Pt 4):352–67. doi: 10.1107/S0907444912001308 ; PubMed Central PMCID: PMCPMC3322595. 22505256 10.1107/S0907444912001308 PMC3322595

[90] Chen VB, Arendall WB 3rd, Headd JJ, Keedy DA, Immormino RM, Kapral GJ, et al MolProbity: all-atom structure validation for macromolecular crystallography. Acta Crystallogr Sect D Biol Crystallogr. 2010;66(Pt 1):12–21. Epub 2010/01/09. doi: 10.1107/S0907444909042073 ; PubMed Central PMCID: PMC2803126. 20057044 10.1107/S0907444909042073 PMC2803126

[91] Urzhumtseva L, Afonine PV, Adams PD, Urzhumtsev A. Crystallographic model quality at a glance. Acta Crystallogr Sect D Biol Crystallogr. 2009;65(Pt 3):297–300. Epub 2009/02/25. doi: 10.1107/S0907444908044296 ; PubMed Central PMCID: PMC2651759. 19237753 10.1107/S0907444908044296 PMC2651759

[92] Larkin MA, Blackshields G, Brown NP, Chenna R, McGettigan PA, McWilliam H, et al Clustal W and Clustal X version 2.0. Bioinformatics. 2007;23(21):2947–8. doi: 10.1093/bioinformatics/btm404 . 17846036 10.1093/bioinformatics/btm404

[93] Ulijasz AT, Cornilescu G, von Stetten D, Kaminski S, Mroginski MA, Zhang J, et al Characterization of two thermostable cyanobacterial phytochromes reveals global movements in the chromophore-binding domain during photoconversion. J Biol Chem. 2008;283(30):21251–66. doi: 10.1074/jbc.M801592200 ; PubMed Central PMCID: PMCPMC3258942. 18480055 10.1074/jbc.M801592200 PMC3258942

[94] Han MV, Zmasek CM. phyloXML: XML for evolutionary biology and comparative genomics. BMC Bioinformatics. 2009;10:356 doi: 10.1186/1471-2105-10-356 ; PubMed Central PMCID: PMCPMC2774328. 19860910 10.1186/1471-2105-10-356 PMC2774328

[95] Kraulis P. MOLSCRIPT: a program to produce both detailed and schematic plots of protein structures. J Appl Crystallogr. 1991;24:946–50.

[96] Schwede T, Kopp J, Guex N, Peitsch MC. SWISS-MODEL: An automated protein homology-modeling server. Nucleic Acids Res. 2003;31(13):3381–5. ; PubMed Central PMCID: PMCPMC168927. 12824332 10.1093/nar/gkg520 PMC168927

