# Supplementary figures and images for: RitR is an archetype for a novel family of redox sensors in the streptococci that has evolved from two-component response regulators and is required for pneumococcal colonization
Source: PLoS Pathog. 2018 May 11;14(5):e1007052. doi: 10.1371/journal.ppat.1007052 (PMC5965902; doi:10.1371/journal.ppat.1007052)

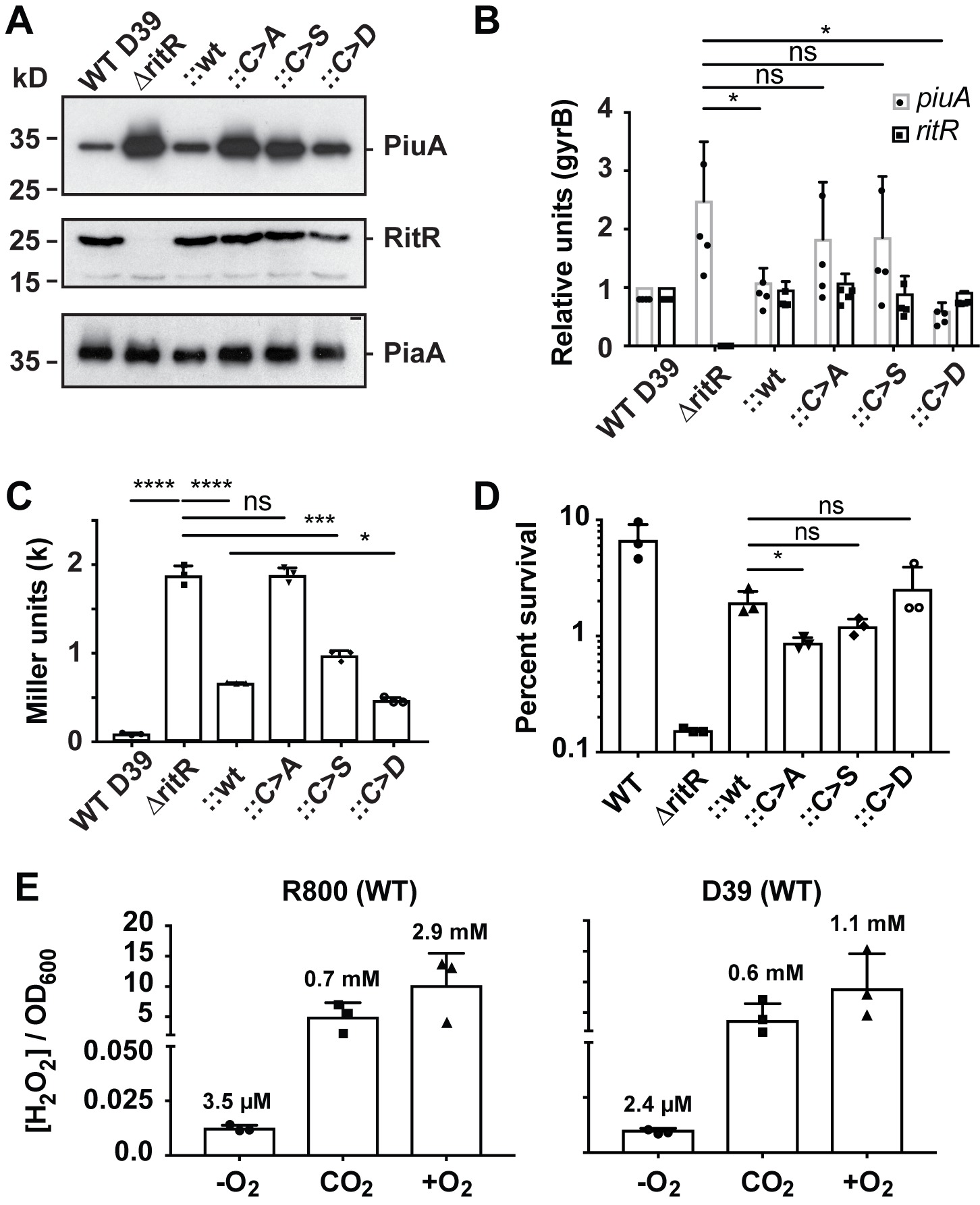

Supplement: S1 Fig — (A) Western blot analysis representative of three independent experiments of PiuA iron transporter lipoprotein levels in the D39 ritR genetic background variants grown in 5% CO2. RitR and PiaA, another iron transporter lipoprotein, are used as controls. (B) qRT-PCR analysis of piuA and ritR mRNA from cells grown in 5% CO2, presented as relative quantities after normalization to gyrB. Error bars represent standard deviation of the mean of 4 independent experiments. Statistics were calculated using a one-way ANOVA followed by Tukey’s multiple comparison test. (C) Piu promoter activity in the genetic background of D39 ritR variants in 5% CO2. (D) Streptonigrin killing of the D39 ritR genetic variants. (E) D39 and R800 WT H2O2 concentration under anaerobic (-O2), 5% CO2 and aerobic (+O2) conditions. Samples of S. pneumoniae cells were cultured to early exponential phase (0.1–0.2) followed by measurement of H2O2 concentration in the culture medium with an Amplex Red detection kit (Invitrogen). H2O2 concentration was normalized to the optical density of the culture. Above the graph bars are the average raw concentrations of H2O2 for comparison. Unless otherwise noted, graphs are a result of three independent experiments and error bars represent +/- standard deviation. One asterisk indicates a p-value of ≤0.05, two of ≤0.01, three of ≤0.001, and four of ≤ 0.0001 as determined by one-way ANOVA followed by Tukey’s multiple comparison test in C. A two-tailed students t-test was used in D and E. ns, not significant. (TIF) [file ppat.1007052.s001.tif]

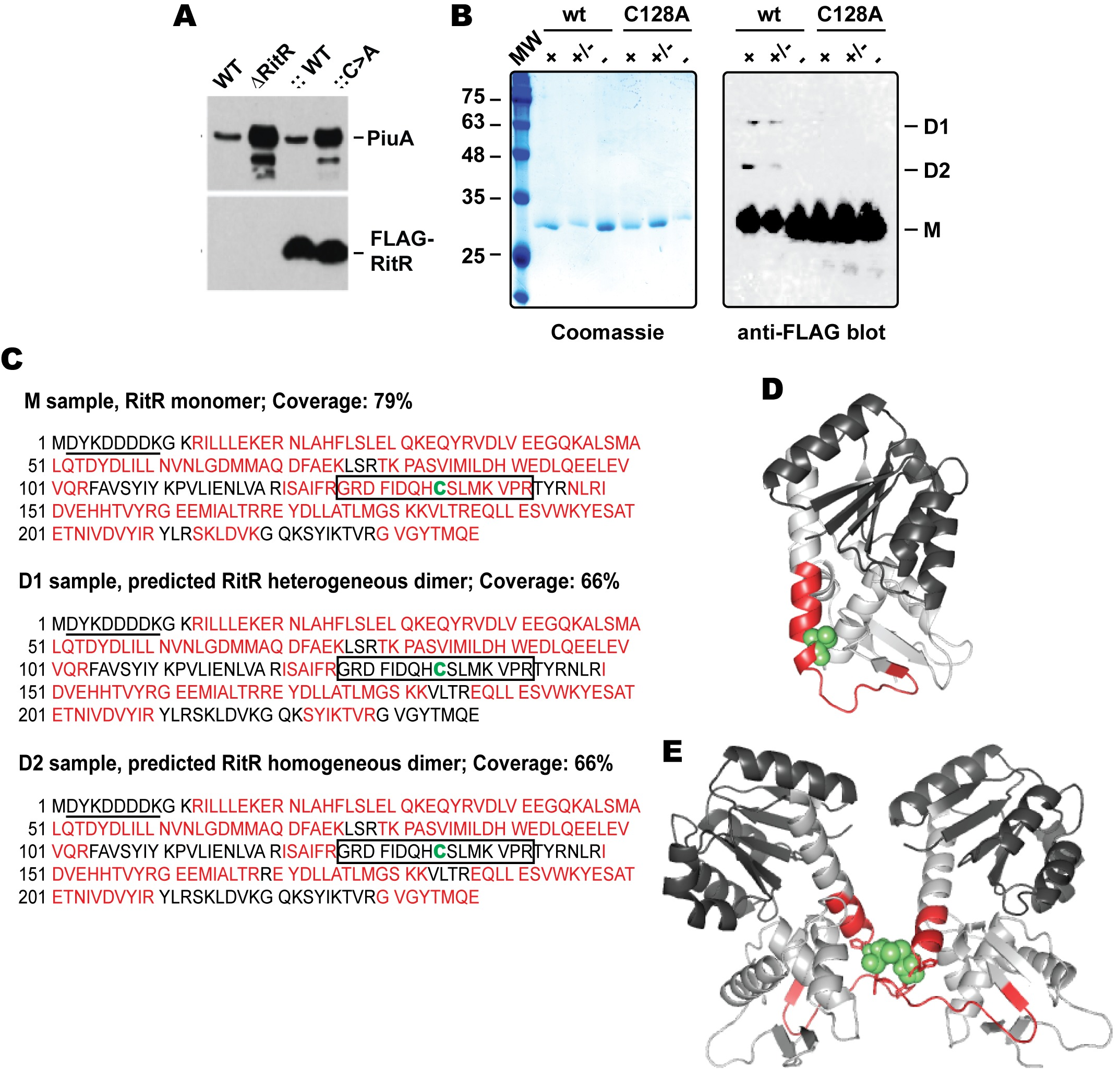

Supplement: S2 Fig — (A) Western blot showing the levels of PiuA and FLAG-RitR expressed in R800 strains used in the experiment in B. (B) Coomassie and anti-FLAG western blot of R800 wild-type (wt) and C128A mutant in vivo immunoprecipitation assay. MW, molecular weight ladder; (+) oxygen = cells were aerated, (+/-) oxygen = cells were grown statically in 5% CO2 and (-) oxygen = cells were grown anaerobically before RitR was immunoprecipitated. D1, predicted dimeric RitR; M, predicted monomeric RitR; D2, RitR-containing heterologous protein complex. (C) Mass spectrometry coverage of D1, D2 and M bands from B. All RitR amino acids/peptides identified by MS are colored red, and ones that were not identified are black. Cys128 is colored green and bolded. Note the absence of the GRDFIDQHCSLMKVPR peptide (boxed) only in the oxidized samples. (D) The GRDFIDQHCSLMKVPR peptide (colored red) with Cys128 (colored green spheres) mapped onto the cartoon representation of the RitR C128S structure and (E) RitR oxidized structure. The REC/ALR domain is shown in black and the linker and DBD in grey. (TIF) [file ppat.1007052.s002.tif]

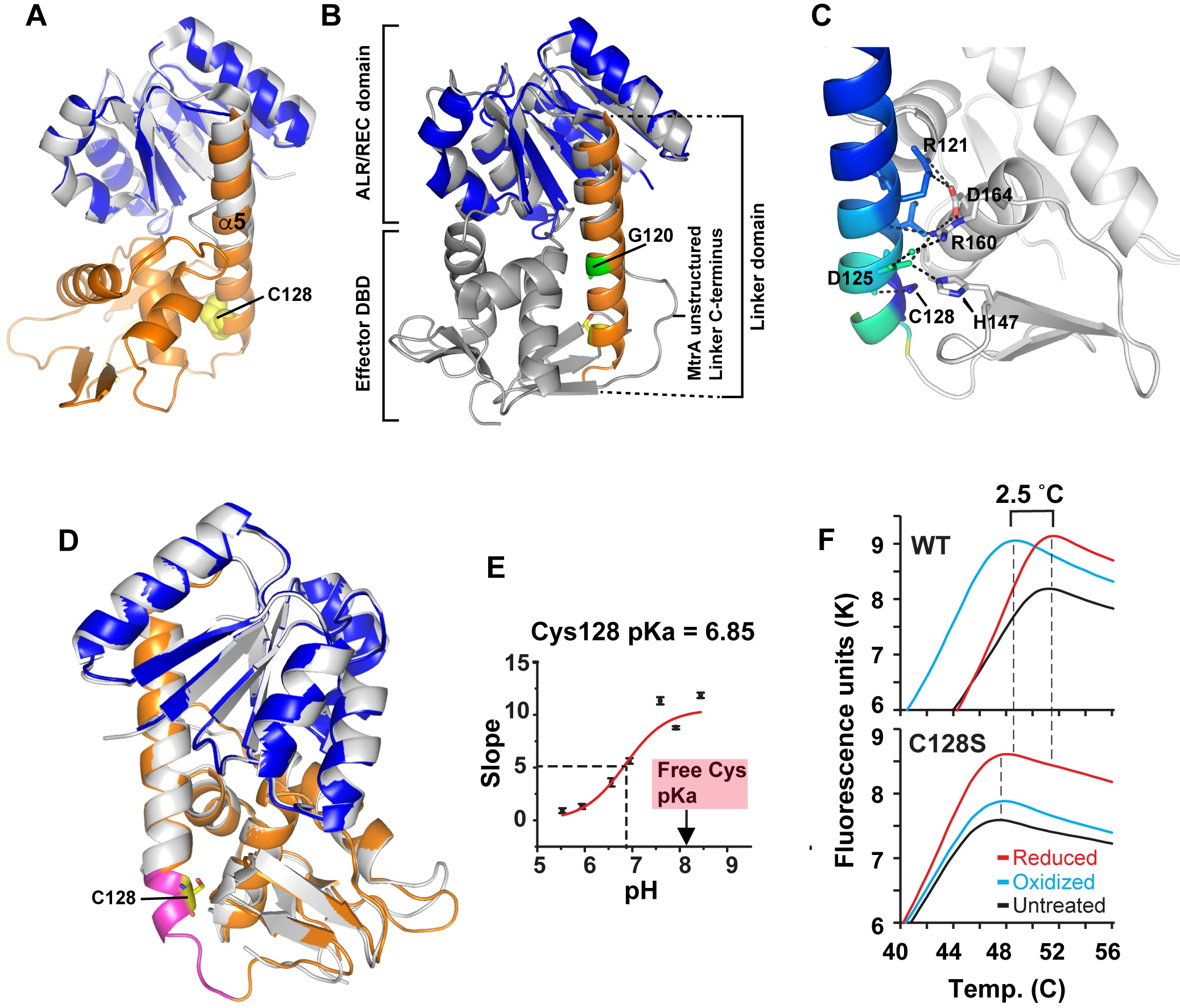

Supplement: S3 Fig — (A) Overlay of the ribbon diagrams for the full-length RitRC128S (ALR/REC domain is colored blue, the linker and DBD colored orange and Ser128 (Cys128) colored yellow) and the RitR ALR/REC domain structure alone (colored grey, PDB ID: 4LZL). (B) Overlay of the C128S RitR structure (in color) and the most similar available full-length structure, MtrA (in grey; PDB ID: 2GWR). The overlay emphasizes the unusual fully structured N-terminal linker helix in RitR (in orange), versus a typical unstructured helix in the MtrA response regulator. The Gly120 hinge is colored green and Ser128 (Cys128) yellow. For clarity the DBD of the RitR C128S structure was omitted from the overlay. (C) Close-up cartoon structural representation of helix 5, Cys128 and nearby electrostatic interactions in the C128S structure. Helix 5 is shown as a heat map according to the structure’s calculated B-factors, where dark blue is more stable than green/yellow. Notice that despite the lack of stability around Cys128, the structure can still maintain its helical integrity, likely through the extensive hydrogen bonding (depicted by black dashed lines). (D) Overlay of RitR C128S (in color) and C128D (colored grey) structures. The region colored magenta in the C128S structure, including Ser128 (i.e. Cys128) itself (colored yellow) is missing electron density in C128D, suggesting that the C>D substitution creates local instability in this region of the protein. (E) Graph showing the DNTB assay indicating that the pKa of Cys128 (pH 6.85) is more acidic than free cysteine (pH 8.5, shown by the arrow). (F) Thermal shift assay comparing wild-type (wt) versus C128S RitR samples. Note that a switch in protein stability (of 2.5°C) only occurs when Cys128 is present in the wild-type sample. K, thousands of fluorescent units. (TIF) [file ppat.1007052.s003.tif]

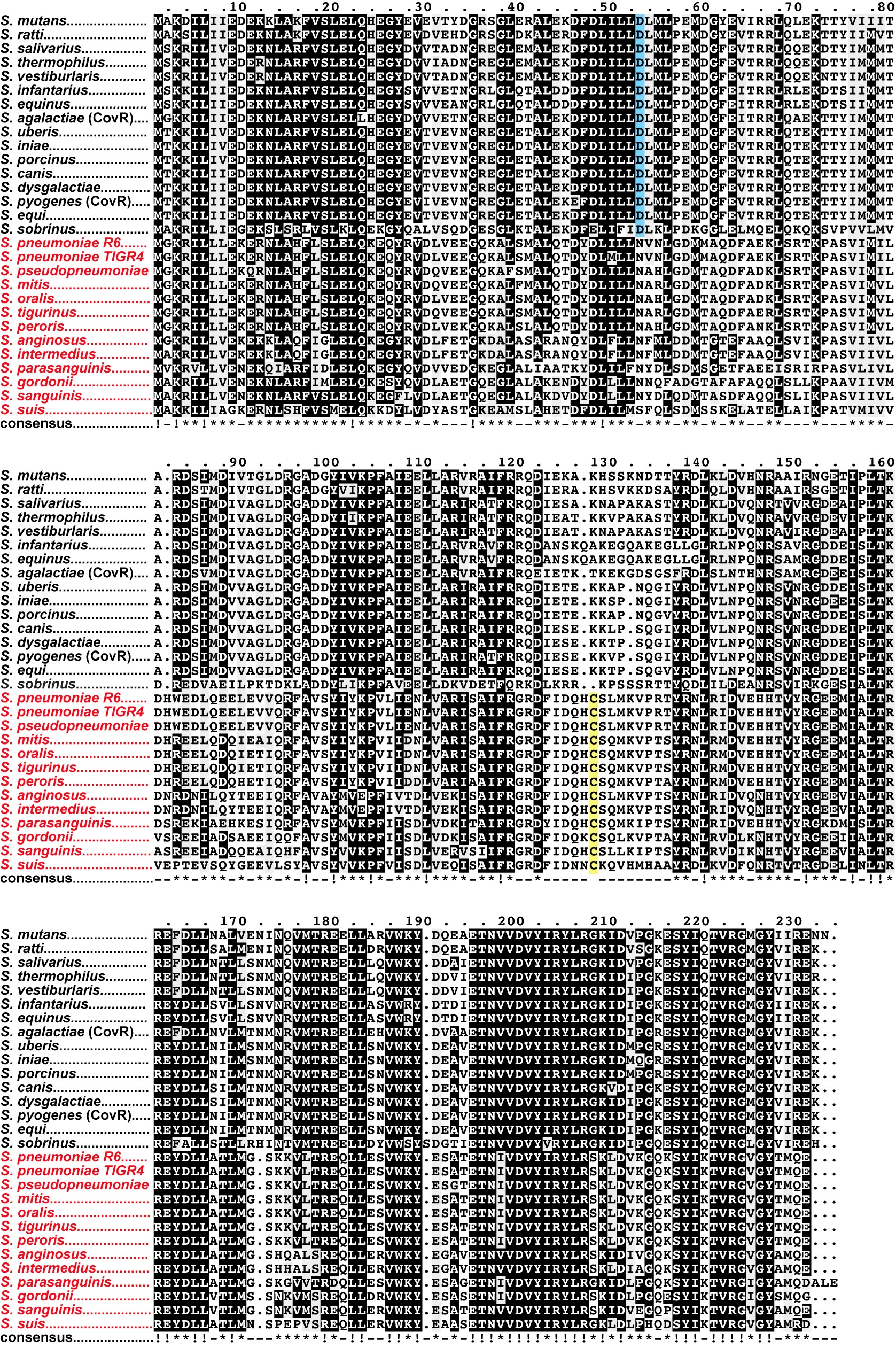

Supplement: S4 Fig — RitR-containing species are colored red and CovR-containing species black. The conserved phosphorylatable Asp residue found in canonical REC domains (i.e. CovR) are shaded in blue, and the conserved Cys128 in RitR homologs shaded in yellow. Identical residues are colored black and similar residues are colored grey. (TIF) [file ppat.1007052.s004.tif]

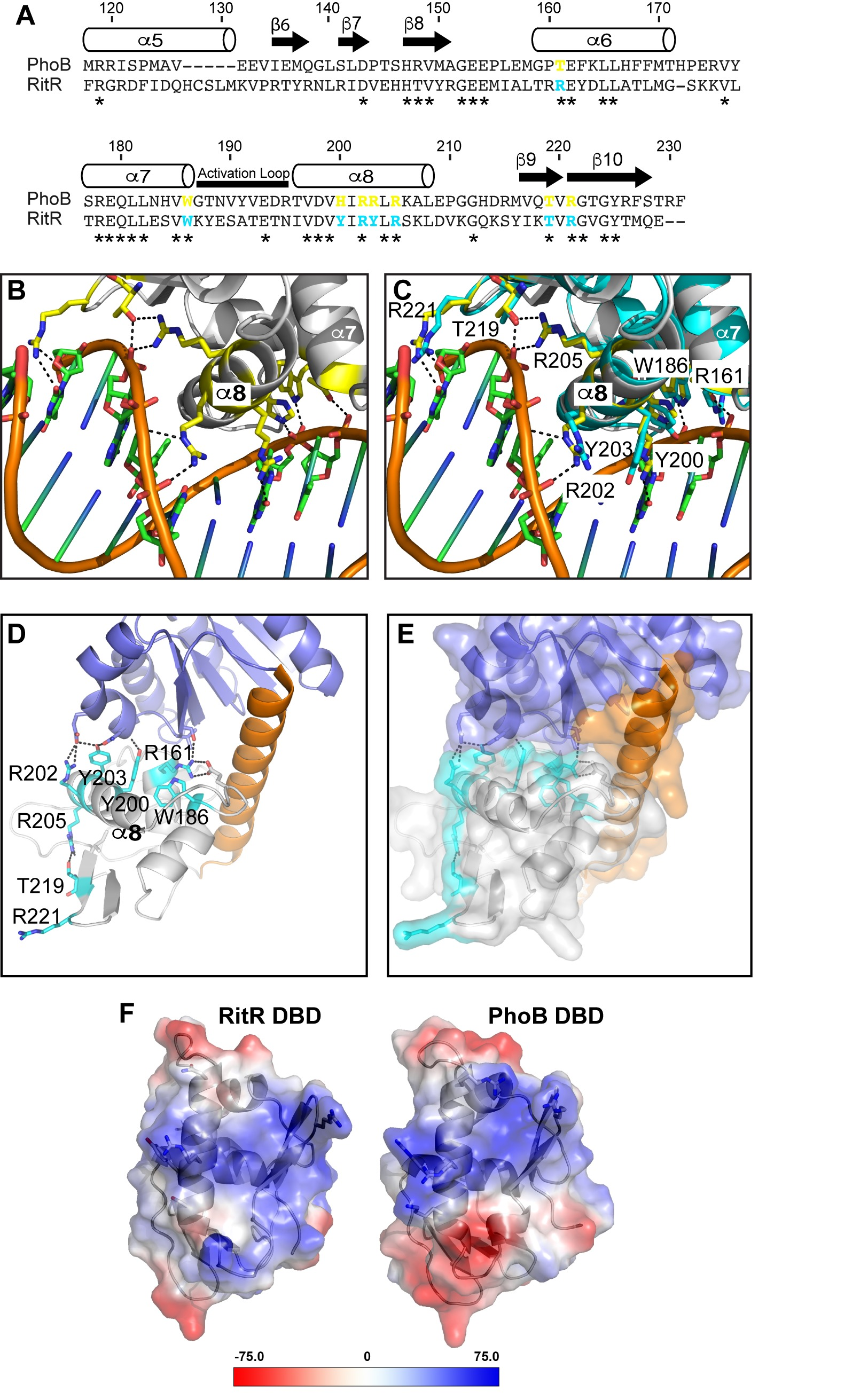

Supplement: S5 Fig — (A) A Clustal Omega alignment of RitR and PhoB DBDs. PhoB residues that contact DNA in the structure (PBD code 1GXP; ref. [47]) are colored in yellow, and corresponding RitR residues in cyan. Asterisks represent identical residues. (B) Cartoon representation of PhoB DBD structure (1GXP) and residues (in yellow) that contact its cognate DNA. Nitrogens are colored blue and oxygens red. Dotted lines indicate probable electrostatic interactions. (C) Cartoon overlay of the PhoB DBD structure from B (in grey) and a RitR structural model (in cyan) based on the alignment in A (using Swiss Model, ref. [96]). Notice the similarities between PhoB and RitR residues that contact DNA and their relative positions. (D) Cartoon representation of the RitRC128S structure showing RitR DBD residues (cyan), which are predicted to bind DNA based on the PhoB 1GXP structure, but instead here form hydrogen bonds to REC domain (blue) residues in the absence of DNA. The DBD is colored grey and the linker domain orange. (E) Transparent surface representation of D showing how RitR residues predicted to bind DNA are sandwiched within the inactive structure. (F) Comparison of RitR DBD and PhoB DBD electrostatics. Negative charges are shown in red and positive charges in blue. Structures are shown as cartoons within a transparent surface representation. All figures were created using MacPyMol. (TIF) [file ppat.1007052.s005.tif]

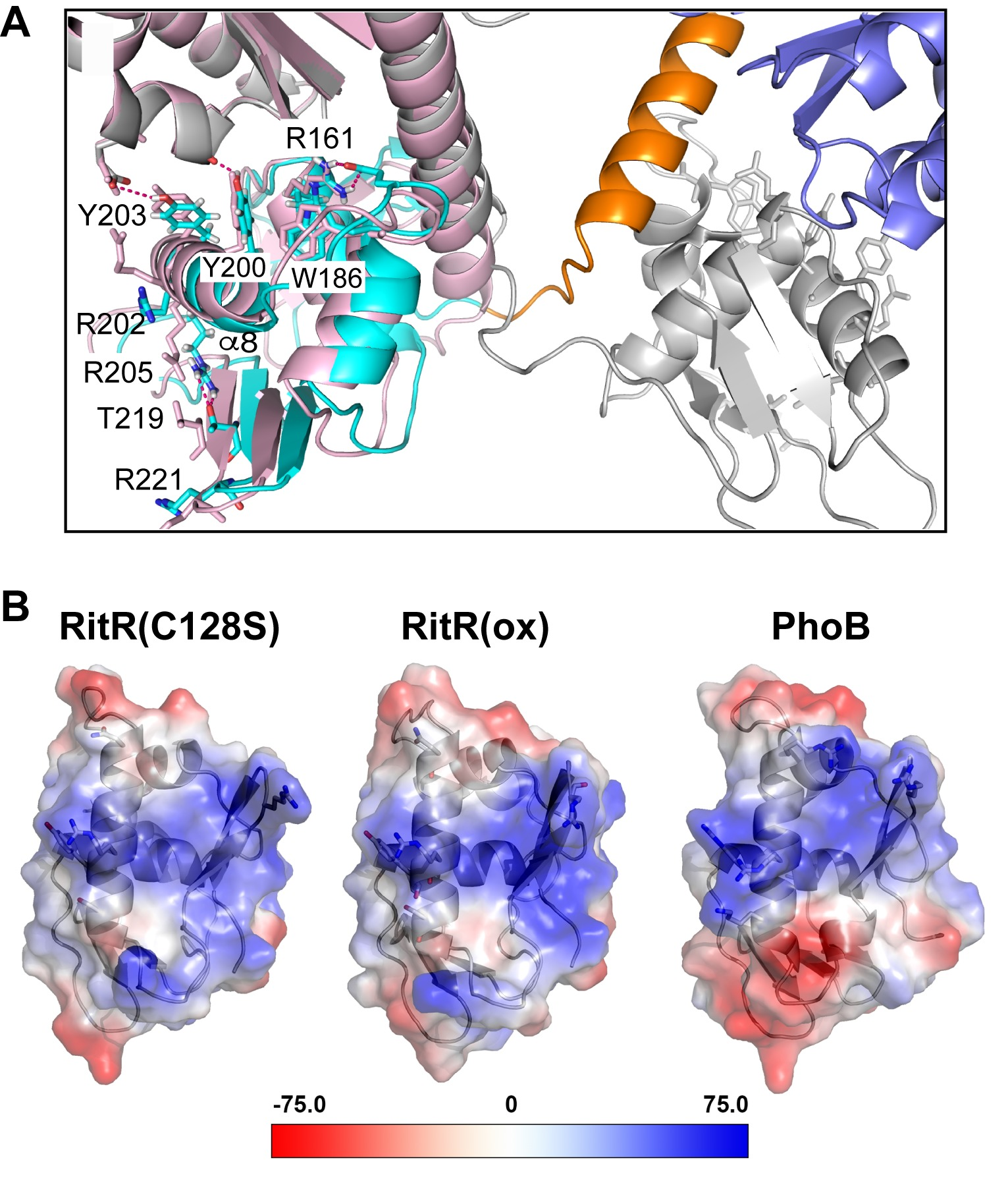

Supplement: S6 Fig — (A) Overlay of a cartoon representation of the DBD of RitRC128S (cyan) and RitROX (pink) structures. The other RitROX protomer (on right side of figure) is colored blue (REC/ALR domain), orange (linker) and grey (DBD). Labeled residues are alleged to make contacts with DNA based on comparisons to the PhoB structure 1GXP [47]. Nitrogens are colored blue and oxygens red. Red dotted lines represent predicted electrostatic interactions between DBD and REC residues. (B) Comparison of RitRC128S DBD, RitROX DBD and PhoB DBD electrostatics. Negative charges are shown in red and positive charges in blue. Structures are shown as cartoons within a transparent surface representation. All figures were created using MacPyMol. (TIF) [file ppat.1007052.s006.tif]

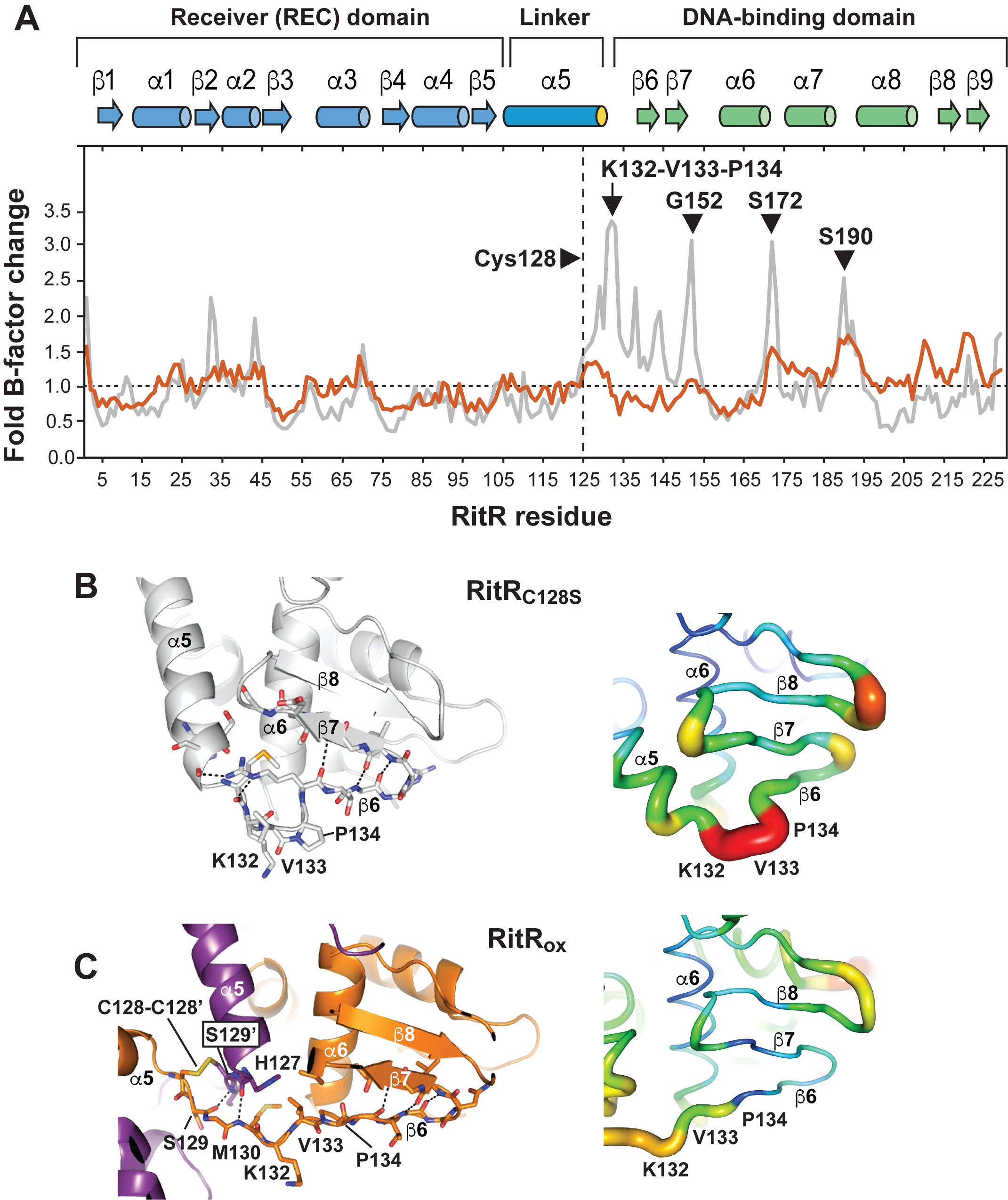

Supplement: S7 Fig — (A) Stability of the RitRC128S and RitROX structures. B-factors of individual alpha carbons were divided by the calculated average B-factor value for the entire structure (y-axis), and graphed in accordance to residue position (x-axis). A value of 1 indicates the average value, and higher and lower numbers indicate less and more stability than the calculated structural average, respectively. The position of Cys128 is indicated by the dotted line. The grey line represents B-factor deviation in the RitRC128S structure, and the orange line the RitROX structure. Key residues that vary greatly in B-factor values from the average in the RitRC128S structure are labeled and indicated by the arrows (B) Cartoon representation (left panel) and B-factor putty representation (right panel) of the β6-β7-β8-α6 region of the RitRC128S structure. Predicted electrostatic interactions are shown as dotted lines. (C) Cartoon representation (left panel) and B-factor putty representation (right panel) of the β6-β7-β8-α6 region of the RitROX structure. In the cartoon the two RitR protomers are colored orange and purple for clarity. Predicted electrostatic interactions are shown as dotted lines. Notice the additional hydrogen bonds provided between Ser129-Ser129’ and Met130-His127’, presumably accounting for enhanced stabilization observed in the domain-swapped structure. (TIF) [file ppat.1007052.s007.tif]

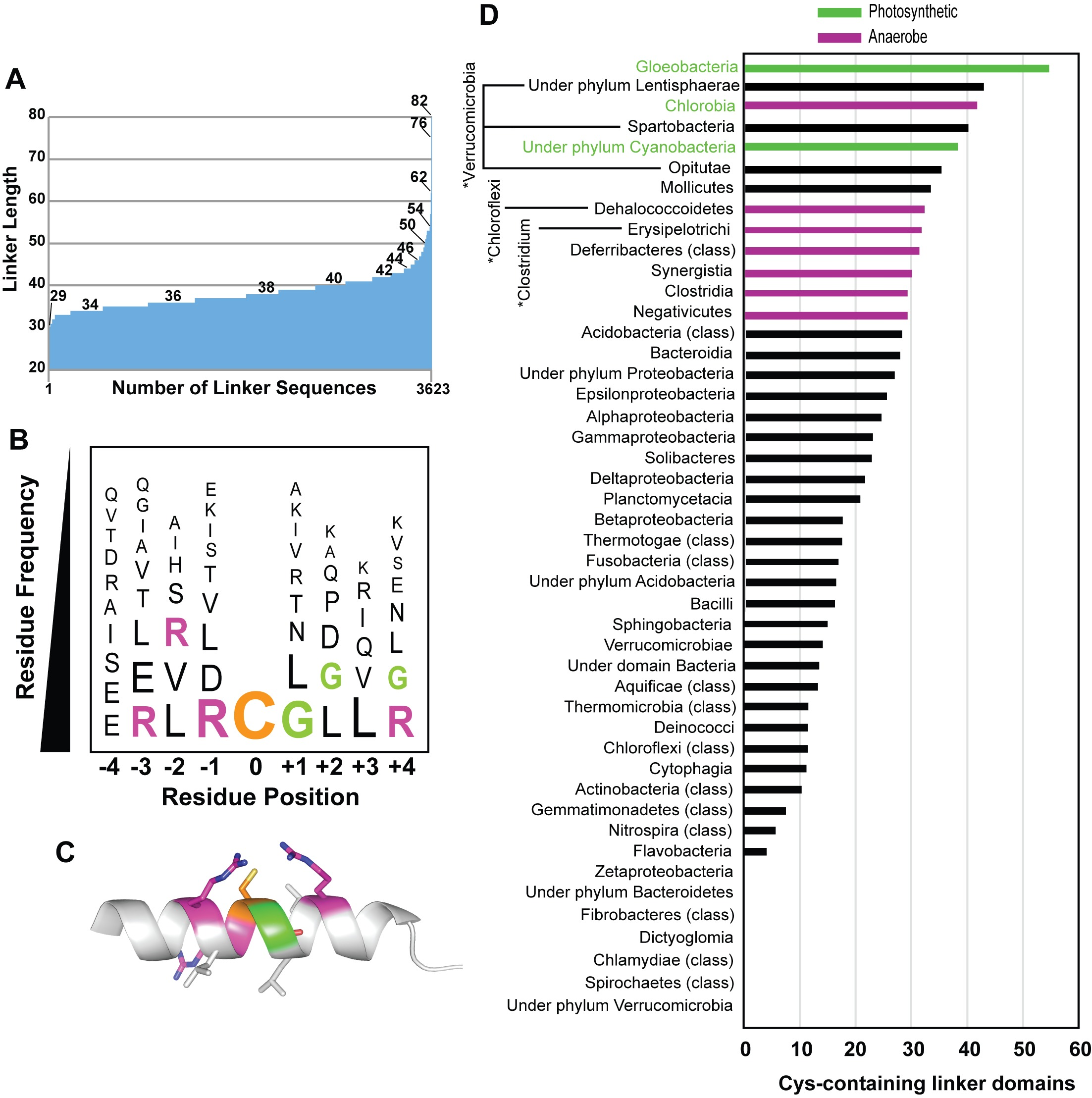

Supplement: S8 Fig — (A) Linker domain length (y-axis) versus the number of linker sequences with the length in question (x-axis). Data were derived from 3,623 total deposited sequences containing an N-terminal REC sensory domain, linker, and C-terminal DNA-binding domain. (B) Logo diagram showing residue frequency (proportional to the letter size; y-axis) versus position from the linker Cys (-4 to +4; x-axis). The cysteine (C) is colored orange, arginines (R) in purple and glycines (G) green. (C) Structural model of a helix containing the most frequent Cys-containing linker peptide ("ERLRCGLLR") showing that arginines are in positions to influence Cys pKa directly, and that the glycines are positioned adjacent to the cysteine for predicted increased helical flexibility. (D) Graph showing the number of Cys-containing linker domains per class of bacteria. Photosynthetic microbes are colored in green, and anaerobes in purple. An asterisk denotes a higher order phyla, e.g. Lentisphaerae, Spartobacteria and Opitutae are all under the Verrucomicrobia phyla. (TIF) [file ppat.1007052.s008.tif]

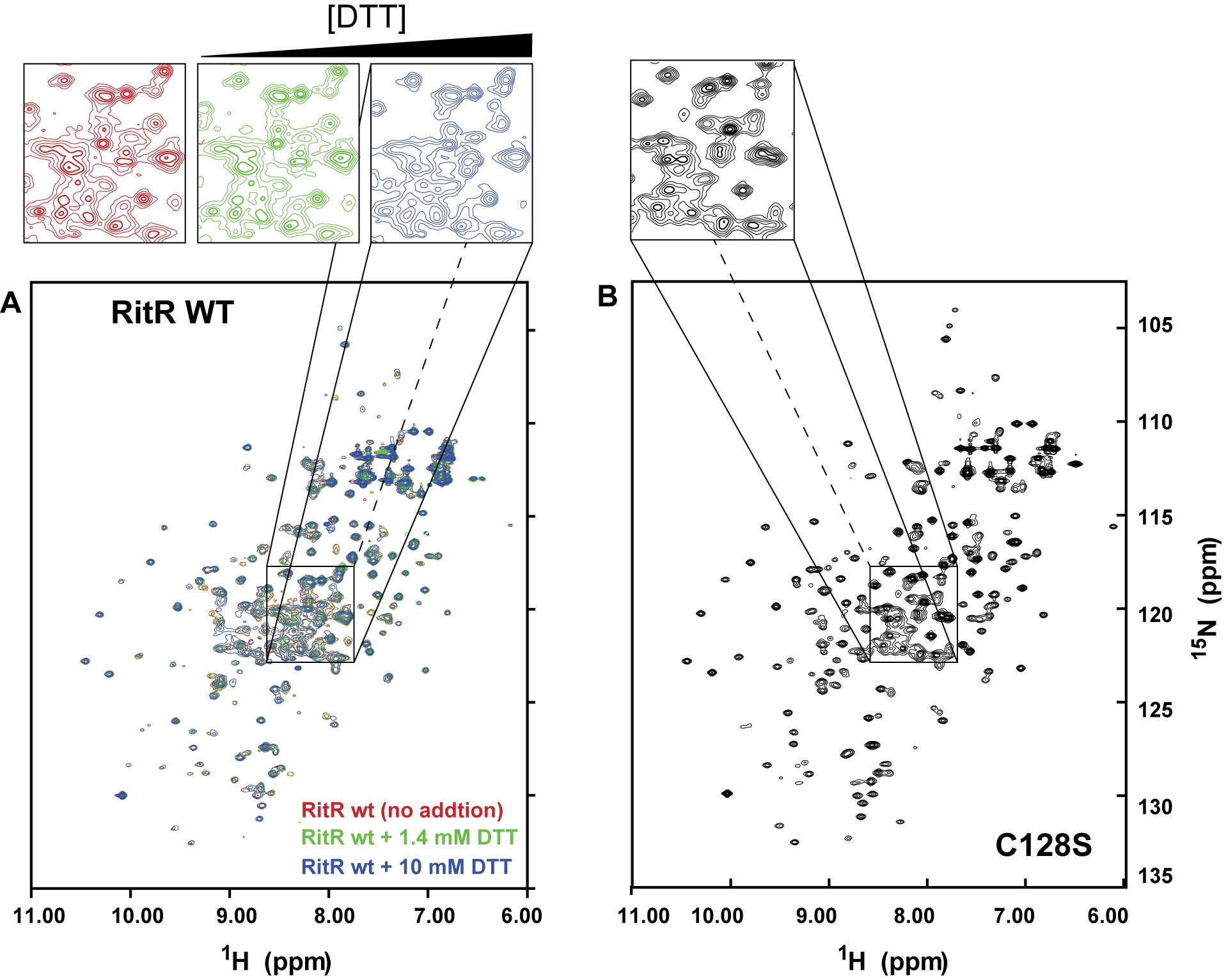

Supplement: S9 Fig — 1H-15N HSQC data were acquired with 15N-labeled RitR wild-type and RitRC128S samples (360 μM, 16 scans). (A) The wild-type sample data was collected first, and then two additional datasets collected on the same (wt) sample after spiking with 1.4 mM DTT and then 10 mM DTT, respectively. All three spectrums are shown in a single overlay. Red spectrum, no DTT added; Green spectrum, 1.4 mM DTT added; Blue spectrum, 10 mM DTT added. Identical representative portions of all three spectrums are shown above the overlay for comparison. Notice the overall chemical shift peaks become less broad as DTT is added, indicating that the structure size is decreasing as the sample becomes reduced (to monomeric form). (B) A RitRC128S HSQC spectrum is shown for comparison. Notice that the RitRC128S spectrum more closely resembles the reduced (monomeric) wild-type sample with 10 mM DTT in A. (TIF) [file ppat.1007052.s009.tif]

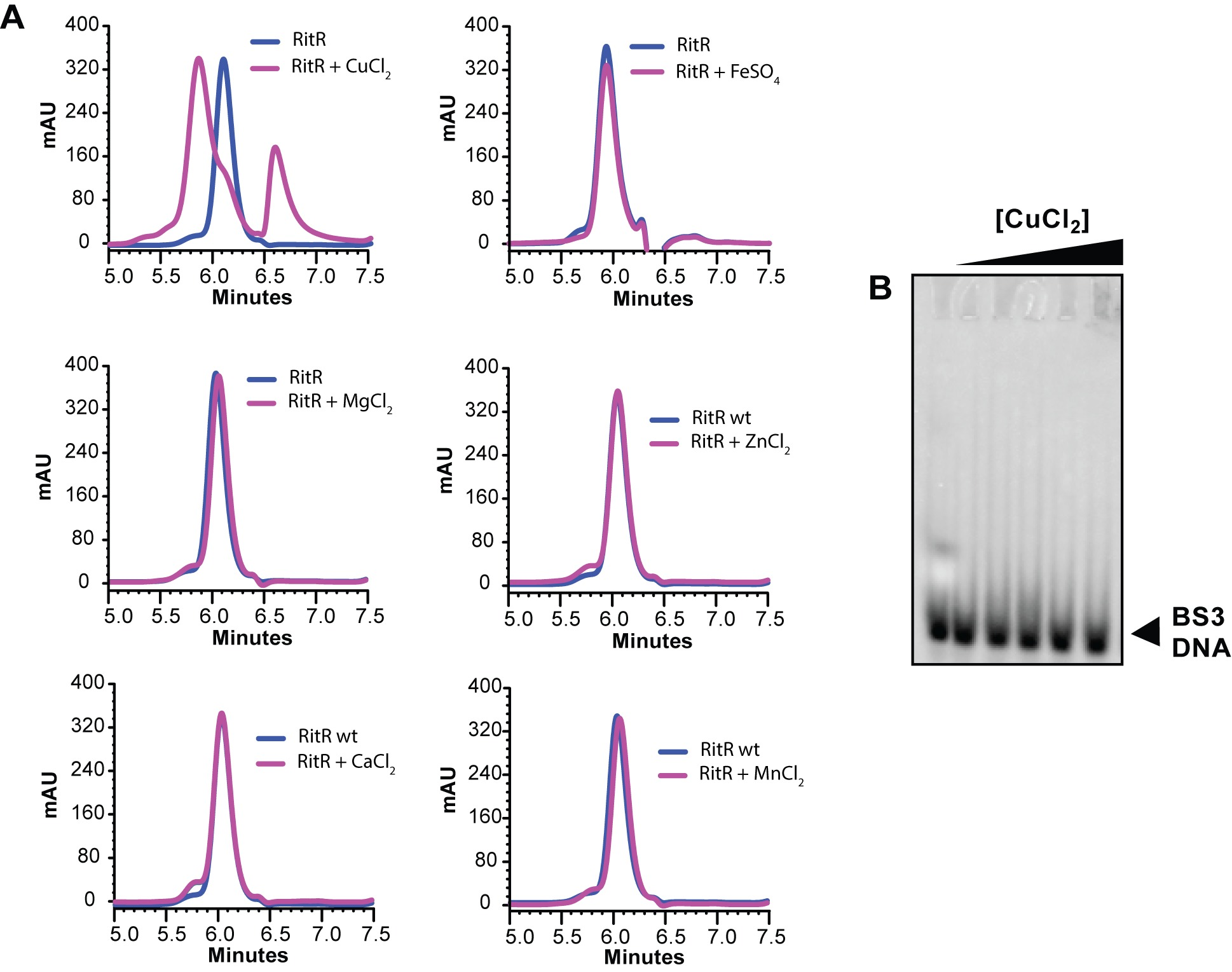

Supplement: S10 Fig — (A) SEC of RitR (116 μM final concentration) wild-type samples before (blue lines) and then after (magenta lines) a pre-incubation with a 1 mM final concentration of the following metals: copper (Cu), iron (Fe), magnesium (Mg), zinc (Zn), calcium (Ca) and manganese (Mn). For comparison, blue lines represent SEC profiles for wild-type RitR (116 μM) with no addition. Notice only copper (CuCl2) is able to cause RitR to dimerize. Conditions of the SEC runs are identical to that of Fig 2. The lower (small) molecular weight peak eluting at 6.6 minutes corresponds to free copper ions. (B) EMSA of the HEX-labeled piu BS3 double stranded oligo and purified RitR (1 μM) with increasing concentrations of CuCl2 (from 0 to 100 μM concentration). No shift is observed, indicating that copper does not influence RitR in binding piu BS3 DNA. Unless otherwise noted here, the EMSA experiment was carried out as per Fig 2. mAU; milli Absorbance Units. (TIF) [file ppat.1007052.s010.tif]
