# Supplementary material for: RitR is an archetype for a novel family of redox sensors in the streptococci that has evolved from two-component response regulators and is required for pneumococcal colonization
Source: PLoS Pathog. 2018 May 11;14(5):e1007052. doi: 10.1371/journal.ppat.1007052 (PMC5965902; doi:10.1371/journal.ppat.1007052)
Supplement: S1 Table — (PDF) [file ppat.1007052.s011.pdf]

Table S1. Plasmids, strains and primers used in this study

**Plasmid**	**Description**	**Source or reference**
pNE1	*E. coli-S. pneumoniae* shuttle vector	Bartilson *et al.* 2001
pNE1-FLAG-RitR (WT)	pNE1 P_{ermAM}-FLAG-RitR (WT)	Maule *et.al.* 2015
pNE1-FLAG-RitR (C128A)	pNE1 P_{ermAM}-FLAG-RitR (C128A)	Maule *et.al.* 2015
pE-SUMO	N-terminal His_6-SUMO fusion protein expression vector	Life Sensors Inc.
pE-SUMO-$RitR_{WT}$	pE-SUMO RitR WT	Maule *et.al.* 2015
pE-SUMO-$RitR_{C128S}$	pE-SUMO RitR C128S	Maule *et.al.* 2015
pE-SUMO-$RitR_{GADDY}$	pE-SUMO RitR GADDY	This work
pE-SUMO-$RitR_{L86A/V96A}$	pE-SUMO RitR L86A/V96A	Maule *et.al.* 2015
pPP2	*S. pneumoniae bgaA* integrative plasmid with promoter-less *E. coli lacZ*	Halfmann *et al.* 2007
pPP2 $P_{piu1\text{-}3}$	pPP2 with *piu* promoter containing RitR binding sites 1-3	This work
**Strain**	**Genotype and or description**	**Source or reference**
E. coli strains		
DH5α	F– Φ80*lacZ*ΔM15 Δ(*lacZYA-argF*) U169 *recA1 endA1* *hsd*R17 (rK–, mK+) *phoA sup*E44 λ– *thi*-1 *gyrA*96 *relA1*	Sambrook *et al.* 1989
BL21(DE3)	F– *ompT hsdSB* (rB–, mB–) *gal dcm* (DE3)	Studier & Moffatt 1986
SUMO-$RitR_{WT}$	BL21(DE3) pE-SUMO His_6-SUMO-RitR WT expression strain	Maule *et.al.* 2015
SUMO-$RitR_{C128S}$	BL21(DE3) pE-SUMO His_6-SUMO-RitR C128S expression strain	Maule *et.al.* 2015

SUMO-$RitR_{C128D}$	BL21(DE3) pE-SUMO His_6-SUMO-RitR C128D expression strain	This work
SUMO-$RitR_{WT}$	BL21(DE3) pE-SUMO His_6-SUMO-RitR WT expression strain	Maule *et.al.* 2015
SUMO-$RitR_{C128S}$	BL21(DE3) pE-SUMO His_6-SUMO-RitR C128S expression strain	Maule *et.al.* 2015
SUMO-$RitR_{C128D}$	BL21(DE3) pE-SUMO His_6-SUMO-RitR C128D expression strain	This work
SUMO-$RitR_{GADDY}$	BL21(DE3) pE-SUMO His_6-SUMO-RitR GADDY expression strain	This work
SUMO-$RitR_{L86A/V96A}$	BL21(DE3) pE-SUMO His_6-SUMO-RitR L86A/V96A expression strain	Maule *et.al.* 2015
***S. pneumoniae* strains**		
R800	R6 derivative, unencapsulated derivative of D39 Hex^+ Kan^s $RecA^+$ Sm^s	Lefevre *et al.* 1979
R800 *rpsL*	R800 carrying L56T mutation in *rpsL* Sm^r	This work
R800 *ritR::kan-rpsL*$^+$	R800 *rpsL* with *kan-rpsL*$^+$ in *ritR* locus Kan^r Sm^s	This work
R800 Δ*ritR*	R800 *rpsL* with marker-less deletion of *ritR* Sm^r	This work
R800 Δ*ritR::ritR* WT	R800 *rpsL* with *ritR* WT reintroduced into *ritR* locus Sm^r	This work
R800 Δ*ritR::ritR* C128A	R800 *rpsL* with *ritR* C128A reintroduced into *ritR* locus Sm^r	This work
R800 Δ*ritR::ritR* C128S	R800 *rpsL* with *ritR* C128S reintroduced into *ritR* locus Sm^r	This work
R800 Δ*ritR::ritR* C128D	R800 *rpsL* with *ritR* C128D reintroduced into *ritR* locus Sm^r	This work
D39	Strain NTCC 7466, encapsulated serotype 2 strain	NTCC, UK
D39 *rpsL*	D39 carrying L56T mutation in *rpsL* Sm^r	This work
D39 *ritR::kan-rpsL*$^+$	D39 *rpsL* with *kan-rpsL*$^+$ cassette in *ritR* locus Kan^r Sm^s	This work
D39 Δ*ritR*	D39 *rpsL* with marker-less deletion of *ritR*	This work
D39 Δ*ritR::ritR* WT	D39 *rpsL* with *ritR* WT reintroduced into *ritR* l ocus Sm^r	This work

D39 Δ*ritR::ritR* C128A	D39 *rpsL* with *ritR* C128A reintroduced into *ritR* locus Sm^r	This work
D39 Δ*ritR::ritR* C128S	D39 *rpsL* with *ritR* C128S reintroduced into *ritR* locus Sm^r	This work
D39 Δ*ritR::ritR* C128D	D39 *rpsL* with *ritR* C128D reintroduced into *ritR* locus Sm^r	This work
R800 *rpsL* P$_{piu1-3}$::*lacZ*	R800 *rpsL* but *bgaA::tetM-P*$_{piu1-3}$*::lacZ* Sm^r Tet^r	This work
R800 Δ*ritR* P$_{piu1-3}$::*lacZ*	R800 Δ*ritR* but *bgaA::tetM-P*$_{piu1-3}$*::lacZ* Sm^r Tet^r	This work
R800 Δ*ritR::ritR* WT P$_{piu1-3}$::*lacZ*	R800 Δ*ritR::ritR* WT but *bgaA::tetM-P*$_{piu1-3}$*:: lacZ* Sm^r Tet^r	This work
R800 Δ*ritR::ritR* C128A P$_{piu1-3}$::*lacZ*	R800 Δ*ritR::ritR* C128A but *bgaA::tetM-P*$_{piu1-3}$*:: lacZ* Sm^r Tet^r	This work
R800 Δ*ritR::ritR* C128S P$_{piu1-3}$::*lacZ*	R800 Δ*ritR::ritR* C128S but *bgaA::tetM-P*$_{piu1-3}$*:: lacZ* Sm^r Tet^r	This work
R800 Δ*ritR::ritR* C128D P$_{piu1-3}$::*lacZ*	R800 Δ*ritR::ritR* C128D but *bgaA::tetM-P*$_{piu1-3}$*:: lacZ* Sm^r Tet^r	This work
D39 *rpsL* P$_{piu1-3}$::*lacZ*	D38 *rpsL* but *bgaA::tetM-P*$_{piu1-3}$*::lacZ* Sm^r Tet^r	This work
D39 Δ*ritR* P$_{piu1-3}$::*lacZ*	D39 Δ*ritR* but *bgaA::tetM-P*$_{piu1-3}$*::lacZ* Sm^r Tet^r	This work
D39 Δ*ritR::ritR* WT P$_{piu1-3}$::*lacZ*	D39 Δ*ritR::ritR* WT but *bgaA::tetM-P*$_{piu1-3}$*:: lacZ* Sm^r Tet^r	This work
D39 Δ*ritR::ritR* C128A P$_{piu1-3}$::*lacZ*	D39 Δ*ritR::ritR* C128A but *bgaA::tetM-P*$_{piu1-3}$*:: lacZ* Sm^r Tet^r	This work
D39 Δ*ritR::ritR* C128S P$_{piu1-3}$::*lacZ*	D39 Δ*ritR::ritR* C128S but *bgaA::tetM-P*$_{piu1-3}$*:: lacZ* Sm^r Tet^r	This work
D39 Δ*ritR::ritR* C128D P$_{piu1-3}$::*lacZ*	D39 Δ*ritR::ritR* C128D but *bgaA::tetM-P*$_{piu1-3}$*:: lacZ* Sm^r Tet^r	This work
R800 *ritR::kan* FLAG-RitR (WT)	R800 Δ*ritR* transformed pNE1-FLAG-RitR (WT) Kan^r Spec^r	Maule *et.al.* 2015
R800 *ritR::kan* FLAG-RitR (C128A)	R800 Δ*ritR* transformed with pNE1-FLAG-RitR (C128A) Kan^r Spec^r	Maule *et.al.* 2015

**Primer name**	**5'- 3' Sequence** [b]	**Gene target**
rpsL lift F	5'-GTAGTTGGCTCTGTTGCAGG-3'	*Upstream of rpsL*
rpsL lift R	5'-GTTAGTTAGCCGATATGAGCTGG-3'	*downstream of rpsL*
rpsL check F	5'-CAACATCGTCCCAAGTCCATAG-3'	*Upstream of rpsL lift F*
rpsL check R	5'-GTTCTCAATGTTTTCATGCTATTGTTGG-3'	*Downstream of rpsL lift R*
ritR lift F	5'-GACAGCTACTTGATTGAAATCACAGC-3'	*Upstream of ritR*
ritR-J up R1	5'-GATTATATCACATTATCCATTAAAAATCAAACGGC	*ritR*

	ATGGCT GACCTACTTATTTTTCGTCATA -3’	
ritR-J F	5’-TATGACGAAAAATAAGTAGGTCAGCCATGCCGTTT GATTTTT AATGGATAATGTGATATAATC -3’	*Janus kan-rpsL*$^{+}$ *cassette*
ritR-J R	5’-CACCACGCACAGTTTTAATGTAGCAGAGACCTGGG CCCCTTTC -3’	*Janus kan-rpsL*$^{+}$ *cassette*
ritR-J down F1	5’-GAAAGGGGCCCAGGTCTCTGCTACATTAAAACTGT GCGTGGTG -3’	*ritR*
ritR lift R	5’-GCCAATCTGTAAGCATCTTACCTG-3’	*Downstream of ritR*
ritR-J up R2	5’- CAACTGCAACTGCTTTTCTATTCTTGCATGGTATAT AA AATCCGTTTCCCCATGGCTG-3’	*ritR*
ritR-J down F2	5’-CAGCCATGGGGAAACGGATTTTATATACCATGCAAG AATAGAAAAGCAGTTGCAGTTG-3’	*ritR*
ritR-J C128A R	5’-GGTCCTTGGAACTTTCATCAGACT***GGC***GTGTTGATC AATGAAGTCCCGAC-3’	*ritR*
ritR-J C128A F	5'-GT CGGGACTTCATTGATCAACAC***GCC***AGTCTGATGAAAG TTCCAAGGACC-3’	*ritR*
ritR-J C128S R	5’-GGTCCTTGGAACTTTCATCAGACT***GCT***GTGTTGATC AATGAAGTCCCGAC -3'	*ritR*
ritR-J C128S F	5'-GT CGGGACTTCATTGATCAACAC***AGC***AGTCTGATGAAAG TTCCAAGGACC-3’	*ritR*
ritR-J C128D R	5’- GGTCCTTGGAACTTTCATCAGACT***GTC***GTGTTGAT CAATGAAGTCCCGAC -3'	*ritR*
ritR-J C128D F	5’-GT CGGGACTTCATTGATCAACAC***GAC***AGTCTGATGAAA GTTCCAAGGACC-3’	*ritR*
ritR check F	5’-GTTCACAATGGTATTGAGTACGGTGATATG -3’	*5' of ritR lift F*
ritR check R	5’-CAGTTACCATAGCTCCTGATGAACGG -3’	*3' of ritR lift R*
Ppiu-F1	5’-*AATT*CTGAAGTTGGCATTCAAAAAATCATGTTC-3’	*Ppiu*
Ppiu-F2	5’-CTGAAGTTGGCATTCAAAAAATCATGTTC-3’	*Ppiu*
Ppiu-R1	5’-*GATCC*GACTAGGAGTAGAAGTAAGCC-3’	*Ppiu BS1-3*
Ppiu-R2	5’-*C*GACTAGGAGTAGAAGTAAGCC-3’	*Ppiu BS1-3*
pPP2-tet-F	5’-CCTAATCGGAAAGGTTTTCAATCCC-3’	*5' of pPP2 integration site*
pPP2-tet-R	5’-CCGATAACGATACCAAGGATGAAAC-3’	*pPP2 tet gene*
pPP2-bga-F	5’-GTCTTGGTTGGAACCAAACCAG-3’	*pPP2 bga gene*

pPP2-bga-R	5’-GTTTCAATCTACTATACAATAAGAGAACG-3’	*3' of pPP2 integration site*
pPP2-seq-F	5’-GCATGCATCGGTACCTGCG-3’	*pPP2 plasmid*
pPP2-seq-R	5’-GTAAAACGACGGGATCAAGATGTTTC -3’	*pPP2 plasmid*
RitR(FL)-F1	5’-<u>*AGGT*</u>ATGGGGAAACGGATTTTATTACTGAG-3’	*ritR*
RitR(FL)-F2	5’-ATGGGGAAACGGATTTTATTACTGAG-3’	*ritR*
RitR(FL)-R1	<u>*CTAG*</u>CCTTCTTGCATGGTATATCCAACACCAC	*ritR*
RitR(FL)-R2	5'-CCTTCTTGCATGGTATATCCAACACCAC	*ritR*
RitR-C128S(FL) F	5'-CGGGACTTCATTGATCAACAC***AGC***-3'	*ritR pE-SUMO*
RitR-C128S(FL) R	5'-***GCT***GTGTTGATCAATGAAGTCCCG-3'	*ritR pE-SUMO*
RitR-C128D(FL) F	5'- CGGGACTTCATTGATCAACAC***GAC*** -3'	*ritR pE-SUMO*
RitR-C128D(FL) R	5'- ***GTC***GTGTTGATCAATGAAGTCCCG -3'	*ritR pE-SUMO*
RitR-GADDY F	5'- GCTGGAAGTTGTTCAGCGT***GGT***GCA***GATGAT***TACAT CTATAAGCCAGTCCTTATC -3'	*ritR pE-SUMO*
RitR-GADDY R	5'- GATAAGGACTGGCTTATAGATGTA***ATCATC***TGC***ACC***AC GCTGAACAACTTCCAGC -3'	*ritR pE-SUMO*
T7 F	5'-TAATACGACTCACTATAGGG-3'	*pE-SUMO*
T7 R	5'GCTAGTTATTGCTCAGCGG-3'	*pE-SUMO*

[a]WT, wild-type; Kan, kanamycin; Sm, streptomycin; Spec, spectinomycin; Tet, tetracycline; *tetM*, tetracycline resistance gene; *bgaA*, pneumococcal endogenous *β-galactosidase* gene; *rpsL,* S12 ribosomal gene
[b]Restriction enzyme sites are underlined and italicized. Mutated sites are in color, bold and italicized.
